# Modeling drug-resistant tuberculosis amplification rates and intervention strategies in Bangladesh

**DOI:** 10.1371/journal.pone.0236112

**Published:** 2020-07-23

**Authors:** Md Abdul Kuddus, Michael T. Meehan, Lisa J. White, Emma S. McBryde, Adeshina I. Adekunle

**Affiliations:** 1 Australian Institute of Tropical Health and Medicine, James Cook University, Townsville, QLD, Australia; 2 College of Medicine and Dentistry, James Cook University, Townsville, QLD, Australia; 3 Department of Mathematics, University of Rajshahi, Rajshahi, Bangladesh; 4 Nuffield Department of Medicine, Big Data Institute, Li Ka Shing Centre for Health Information and Discovery, University of Oxford, Oxford, United Kingdom; 5 Decision Sciences Program, Victoria University, Melbourne, Australia; Jamia Hamdard, INDIA

## Abstract

Tuberculosis (TB) is the seventh leading cause of morbidity and mortality in Bangladesh. Although the National TB control program (NTP) of Bangladesh is implementing its nationwide TB control strategies, more specific and effective single or combination interventions are needed to control drug-susceptible (DS) and multi-drug resistant (MDR) TB. In this study, we developed a two strain TB mathematical model with amplification and fit it to the Bangladesh TB data to understand the transmission dynamics of DS and MDR TB. Sensitivity analysis was used to identify important parameters. We evaluated the cost-effectiveness of varying combinations of four basic control strategies including distancing, latent case finding, case holding and active case finding, all within the optimal control framework. From our fitting, the model with different transmission rates between DS and MDR TB best captured the Bangladesh TB reported case counts. The estimated basic reproduction number for DS TB was 1.14 and for MDR TB was 0.54, with an amplification rate of 0.011 per year. The sensitivity analysis also indicated that the transmission rates for both DS and MDR TB had the largest influence on prevalence. To reduce the burden of TB (both DS and MDR), our finding suggested that a quadruple control strategy that combines distancing control, latent case finding, case holding and active case finding is the most cost-effective. Alternative strategies can be adopted to curb TB depending on availability of resources and policy makers’ decisions.

## 1. Introduction

TB is an airborne bacterial infection that causes millions of deaths worldwide each year [1]. The TB bacteria (*Mycobacterium tuberculosis* (Mtb)) generally enter the body through the lungs, spreading to other parts of the body through the bloodstream, the lymphatic system, or through direct extension to additional organs (extra-pulmonary TB) [2, 3]. Following an infectious person coughing, sneezing, speaking or singing thousands or tens of thousands of droplet nuclei are created [4]. These minute droplet nuclei can remain suspended in the air for several minutes to an hour, allowing spread to other persons through inhalation [4, 5].

Once infected, the individual will first undergo a period without visible clinical symptoms, called latent TB infection (LTBI). The latent period is the timespan from the point of infection to the beginning of the state of infectiousness, and may last for weeks, months or the entire life of the infected individual. In fact, the lifetime risk of progression to active TB for a person with LTBI is around 5–15%, depending on the age at infection. For those who do progress from LTBI to active TB, the majority will do so within the first two years of initial infection [6].

In Bangladesh, TB is one of the most important public health problems. Globally, Bangladesh has the 7^th^ largest TB incidence in the world and it is estimated that 70,000 people die of TB and 300,000 new cases are generated each year [7]. Moreover, Bangladesh is ranked 10^th^ among the 27 high MDR TB burden countries. Thus, there is a great need to reduce TB incidence, prevalence, and mortality in Bangladesh [8].

In Bangladesh, under the Ministry of Health and Family Welfare, the National TB Control Program (NTP) of the Directorate General of Health Services (DGHS) provides nationwide TB control services. These services include screening, case detection through diagnosis, treatment following appropriate regimen, follow up and evaluation in all areas [9]. The goals of this program are to reduce illness, death and transmission of TB, and to achieve universal high quality service for all people with active and latent TB [10]. More than 44 partner organizations (NGOs) also support the NTP in all areas, including advocacy, communication, and social mobilization (ACSM) activities. The NTP adopted the recent WHO recommended strategies -namely the DOTS Strategy-1993, the Stop TB Strategy-2006, and the End TB Strategy-2015 for its TB control [9, 11].

Mathematical modelling is one of the most important tools for understanding TB transmission dynamics and for predicting the epidemic trajectories [12–18]. In the last few decades, mathematicians and public health professionals have developed different types of mathematical models to investigate TB disease dynamics in different endemic regions. For example, Kim *et al*. [13] constructed a mathematical model for TB with exogenous reinfection and examined the current situation of active TB incidence in Korea, and found that case detection was the most important intervention for decreasing active TB cases. Yang *et al*. [14] developed another TB model with seasonality and determined that seasonality has a high impact on TB related incidence, prevalence and mortality, especially in the winter season. Brooks *et al*. [15] developed a TB mathematical model with survivorship to discover the impact of age structure on the prevalence of TB, the basic reproduction number, and the effect of control interventions. Mishra and Srivastava [16] constructed a transmission dynamic mathematical model to simulate the spread of TB disease in the human population of Jharkhand, India, for DS and MDR TB cases with vaccination [16]. A 10-compartmental TB model constructed by Trauer *et al*. [17] modelled limited vaccine effectiveness, reinfection, MDR TB, and de novo resistance through treatment [17].

In this study, we develop a two strain TB model to describe the transmission dynamics of DS and MDR TB in Bangladesh. We perform a sensitivity analysis to explore the impact of model parameters. The model is calibrated to the TB Bangladesh data to estimate amplification rate and other key transmission parameters such as infection and treatment rates. Based on the calibration, four different control strategies or policies are considered. Several scenarios are examined to explore the optimal control policy for reducing the spread of DS and MDR TB. The purpose of optimal control is to decrease the prevalence of DS and MDR TB as well as to minimize the cost incurred in the implementation of control procedures. To the author’s best knowledge, this study is the first TB model to characterise the TB amplification rate in Bangladesh and use the result to identify optimal control strategies.

## 2. Material and methods

### 2.1 Bangladesh TB epidemiological data

Bangladesh is a TB disease endemic country in South-East Asia [1]. Control of TB in such a resource-scare country should be informed by an in-depth mathematical and epidemiological understanding of the disease. This study is based on the yearly reported Bangladesh DS and MDR TB incidence data that was obtained from the WHO report from 2000 to 2018 [19–21]. For this data, TB incidence is separated into patients who had DS or MDR TB and does not include prevalence rates for the years 2000, 2001 and 2002. We estimated prevalence rates for these years by fitting a linear model to our prevalence data in GraphPad Prism [22].

The Bangladesh TB data that were made available has a single distinction between DS TB and MDR TB. The DS TB are all patients with TB strains that are fully-susceptible to all the first-line anti-TB drugs or have resistance to first line anti-mycobacterial agents other than rifampicin, while MDR TB is regarded as at least rifampicin-resistant (on GeneXpert) and in the case of cultured isolates, is rifampicin and isoniazid resistant. The MDR TB tests were conducted using GeneXpert as first line testing for rifampicin followed by culturing and sensitivity testing for other drugs. Extensive drug resistant (XDR) is a subset of MDR TB and is not reported separately in Bangladesh.

### 2.2 Model description

We developed a deterministic transmission dynamics mathematical model of DS and MDR TB strains between the following mutually exclusive compartments: susceptible individuals, S(t); those exposed to TB or latently infected, L(t), representing those that are infected and have not yet developed active TB; the infectious I(t), containing individuals with active TB that are infectious; the recovered R(t) who were previously infected and successfully recovered either naturally or through treatment. The subscripts s and m denote variables associated with the DS strain and MDR strain respectively. We assume that MDR strains were initially generated through inadequate treatment of DS TB, i.e. amplification, and that these strains could subsequently be transmitted to other individuals.

The total population size N(t) is assumed to be constant and well mixed:

$$N(t)=S(t)+L_{s}(t)+L_{m}(t)+I_{s}(t)+I_{m}(t)+R(t).$$ (1)

To ensure the population size remains constant, we replace all deaths as newborns in the susceptible compartment. This includes death through natural causes, which occurs in all states at the constant per-capita rate μ, and TB-related deaths, which occur at the constant per-capita rate ϕ_i_ (i = s,m). Individuals may also return to the susceptible compartment following recovery at the constant per-capita rate γ.

**Fig 1:**
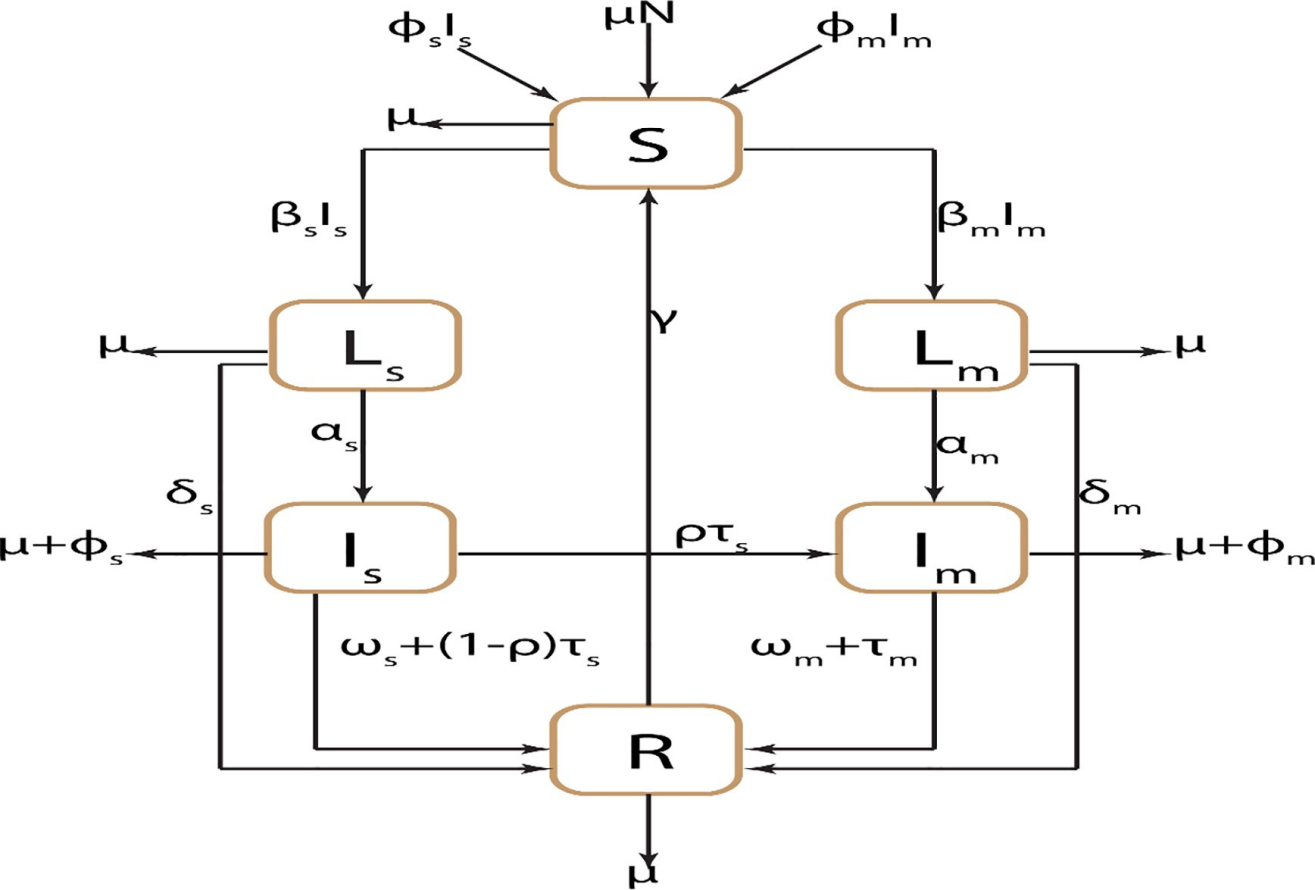
Schematic diagram of two-strain TB transmission model for Bangladesh TB setting.

Susceptible individuals may be infected with a circulating strain of TB at the rate λ_i_ = β_i_I_i_(t) and move to the corresponding latently infected compartment L_i_(t). Here, β_i_ is the probability a susceptible individual contracts infection after contact with an infectious individual with TB strain i (i = s,m). Those with latent infection progress to active tuberculosis as a result of endogenous reactivation of the latent bacilli at the rate α_i_; however, some latent individuals do not progress to the infectious class I_i_(t) but instead undergo endogenous recovery and move directly to the recovery class R(t) at a per capital rate δ_i_ (i = s,m). Individuals with DS and MDR active TB, I_i_(t) may eventually be detected and treated at rates τ_s_ and τ_m_ respectively. A proportion (1−ρ)τ_s_ of the treated DS active TB individuals fully recover to move into the recovered compartment R(t); whilst the complementary ρτ_s_ (amplification rate) develop MDR TB due to incomplete treatment or lack of strict compliance in the use of first-line drugs (drugs used to treat the DS forms of tuberculosis)–and move into the compartment I_m_(t). Furthermore, individuals in the compartment I_i_(t) recover naturally and move into the recovered class R(t) at the rate ω_i_. Active tuberculosis cases in I_i_ (i = s,m) experience disease related death at a rate φ_i_(i = s,m). The model flow diagram is presented in Fig 1.

From the aforementioned, the DS and MDR TB model is given by the following deterministic system of nonlinear ordinary differential equations:

$$\frac{dS}{dt}=\mu N-\beta_{s}I_{s}S-\beta_{m}I_{m}S-\mu S+\gamma R+\varphi_{s}I_{s}+\varphi_{m}I_{m},$$

$$\frac{dL_{s}}{dt}=\beta_{s}I_{s}S-\alpha_{s}L_{s}-\delta_{s}L_{s}-\mu L_{s},$$

$$\frac{dI_{s}}{dt}=\alpha_{s}L_{s}-(1-\rho )\tau_{s}I_{s}-\rho \tau_{s}I_{s}-\varphi_{s}I_{s}-\omega_{s}I_{s}-\mu I_{s},$$

$$\frac{dL_{m}}{dt}=\beta_{m}I_{m}S-\alpha_{m}L_{m}-\delta_{m}L_{m}-\mu L_{m},$$

$$\frac{dI_{m}}{dt}=\alpha_{m}L_{m}+\rho \tau_{s}I_{s}-\omega_{m}I_{m}-\varphi_{m}I_{m}-\tau_{m}I_{m}-\mu I_{m},$$

$$\frac{dR}{dt}=(1-\rho )\tau_{s}I_{s}+\tau_{m}I_{m}+\omega_{s}I_{s}+\omega_{m}I_{m}+\delta_{s}L_{s}+\delta_{m}L_{m}-\gamma R-\mu R.$$ (2)

### 2.3 Model calibration and control strategies

We estimated the TB model parameters from fitting different combinations of parameters in Eq (2) to the actual DS and MDR TB incidence data from Bangladesh [1, 10]. In order to parameterize the TB model (2), we obtained some of the parameter values from the literature (see Table 1), whilst others were estimated from fitting to the data. The aim is to determine the rate of amplification of MDR TB and its dynamics. Note that in quantifying the amplification rate, both drug-susceptible and drug-resistant treatment rates are also determined.

**Table 1:** Parameter description and estimates for Bangladesh TB model (2).

Parameter	Description	Estimated value (range)	References
N	Population at year 2000	137,439,261	[26]
μ	Birth/Death rate	$\frac{1}{70}yr^{-1}$	[27]
β_s_	Transmission rate for DS TB	1.54×10^−8^ yr^−1^	data fitted
Βm	Transmission rate for MDR TB	5.9×10^−9^ yr^−1^	data fitted
α_s_	Activation rate from L_s_ to I_s_	0.116 yr^−1^	[17, 28]
α_m_	Activation rate from L_m_ to I_m_	0.116 yr^−1^	[17, 28]
ω_s_	Recovery rate for DS TB	0.2873 yr^−1^	[29]
ω_m_	Recovery rate for MDR TB	0.12 yr^−1^	assumed
δ_s_	Recovery rate from L_s_ to R	0.108 yr^−1^	estimated
δ_m_	Recovery rate from L_m_ to R	0.108 yr^−1^	estimated
ρ	Proportion of amplification	[0–0.34]	data fitted
φ_s_	Disease-related death rate for DS TB	0.37 over 3 years	[17]
φ_m_	Disease-related death rate for MDR TB	0.37 over 3 years	[17]
τ_s_	Treatment rate for DS TB	0.470 yr^−1^	data fitted
τ_m_	Treatment rate for MDR TB	0.470 yr^−1^	data fitted
γ	Rate of waning immunity	0.10 yr^−1^	[30]
τ_1_	Treatment rate for latent DS TB	0.2 yr^−1^	[31]
τ_2_	Treatment rate for latent MDR TB	0.2 yr^−1^	[31]

Hence, the models we consider are:

- Model 1 (Drug-failure only): β_s_, β_m_ = 0, τ_s_, τ_m_ = 0 and ρ (3 parameter model). Here, we assumed the dynamics of MDR TB are purely driven by drug-failure from DS TB.
- Model 2 (Drug-failure and equal transmission and treatment rates between DS and MDR TB): β_s_ = β_m_, τ_s_ = τ_m_ and ρ (3 parameter model). Here, we assumed the fitness cost of MDR TB is negligible and the treatment outcome is the same for both DS and MDR TB.
- Model 3 (Drug-failure and unequal transmission rates between DS and MDR TB): β_s_ ≠ β_m_, τ_s_ = τ_m_ and ρ (4 parameter model). Here, we assumed the fitness cost to MDR TB is significant and the treatment outcome is the same for both DS and MDR TB.
- Model 4 (Drug-failure and unequal treatment rates between DS and MDR TB): β_s_ = β_m_, τ_s_ ≠ τ_m_ and ρ (4 parameter model). Here, we assumed the fitness cost to MDR TB is negligible and the treatment outcome differs between DS and MDR TB.
- Model 5 (Drug failure and unequal transmission and treatment rates between DS and MDR TB): β_s_ ≠ β_m_, τ_s_ ≠ τ_m_ and ρ (5 parameter model). Here, we assumed the fitness cost to MDR TB is significant and the treatment outcome differs between both DS and MDR TB.

With other parameters derived from the literature (see Table 1), the models were fitted in MATLAB using the multi-start algorithm with 1000 starting points [23]. The convergent results are kept and the confidence intervals are constructed assuming that the estimates are approximately normally distributed. The best model selection is done by using the associated Akaike Information Criterion (AIC) of the model fit [24]. Further we performed both global and local sensitivity analyses of the model parameters to determine the parameters that have the most influence on the equilibrium prevalence and basic reproduction number of TB.

We further use the parameters from the best model fit from above to mitigate the spread of TB in Bangladesh by developing four combination strategies using these four control strategies:

- u_1_(t) (the distancing control strategy)–that is the effort of preventing susceptible individuals from getting exposed to TB bacilli. This includes personal respiratory protection, environmental controls, diagnosis campaigns, and educational programs for public health.
- u_2_(t) (latent case finding)–which includes chemoprophylaxis treatment, high-risk exposure screening and other forms of latent TB treatment. WHO estimated that treatment for LTBI can decrease the risk of developing active TB by at least 60% [1].
- u_3_(t) (case holding)–this refers to activities that ensure treatment completion to reduce relapse following treatment. Patients receiving treatment for either DS or MDR TB should be monitored to ensure the completion of the whole course of treatment. Otherwise, TB infection may become resistant to existing antibiotics.
- u_4_(t) (active case finding)–this represents the prevention of disease development with effective treatment for exposed persons or identification of active TB cases.

The resulting optimal control problems are solved using the forward-backward sweep method [25] and implemented in MATLAB [23]. The outputs from this simulation are subjected to cost-effective analysis using the incremental cost-effectiveness ratio (ICER) to determine the intervention strategy that is the best value for money.

#### Ethical approval

This study is based on aggregated TB surveillance data in Bangladesh provided by the National TB Control Program (NTP) and the World Health Organization (WHO). No confidential information was included because mathematical analyses were performed at the aggregate level. All of the methods were conducted under the approved research protocol. The research protocol was approved by the James Cook University human ethics approval board, H7300.

## 3. Results

### 3.1 Bangladesh TB prevalence rates

**Fig 2:**
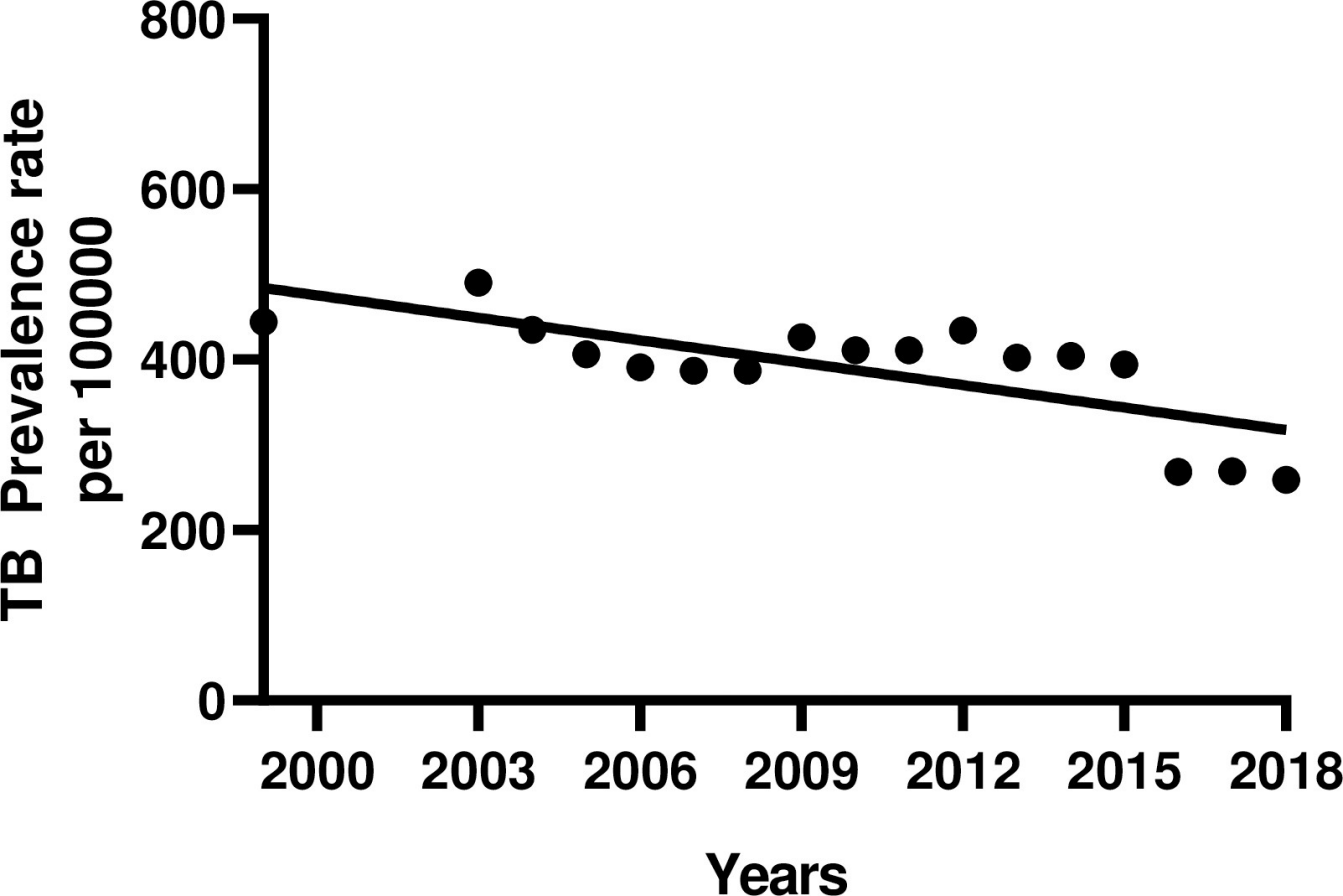
Linear fit to Bangladesh TB prevalence data.

There were four TB surveillance studies conducted in Bangladesh between 1964 and 2015 [32]. Hence the TB prevalence rates are not available for years 2000, 2001 and 2002. To determine the TB prevalence rates for the missing years, we first fitted a linear model to the available TB prevalence rates (Fig 2). The slope of the linear fit was -8.51 (95% CI: -10.40, -6.67) and the y-intercept is 481 at the year 1999. Using this model to predict the TB prevalence for the missing years 2000, 2001 and 2002, we found the estimated prevalence rates per 100,000 people were 473, 464, 456. The decline in prevalence rate is justifiable considering that DOTS was introduced in 1993 and 100% DOTs coverage was reached in 2003. By 2000, the treatment success rate of the DOTs program had reached the targeted 85% and has been maintained above 90% since 2005. In 2006, the program successfully treated 94% of notified new smear-positive cases and the case detection rate was about 70%.

## 3.2 TB dynamical process in Bangladesh

**Fig 3:**
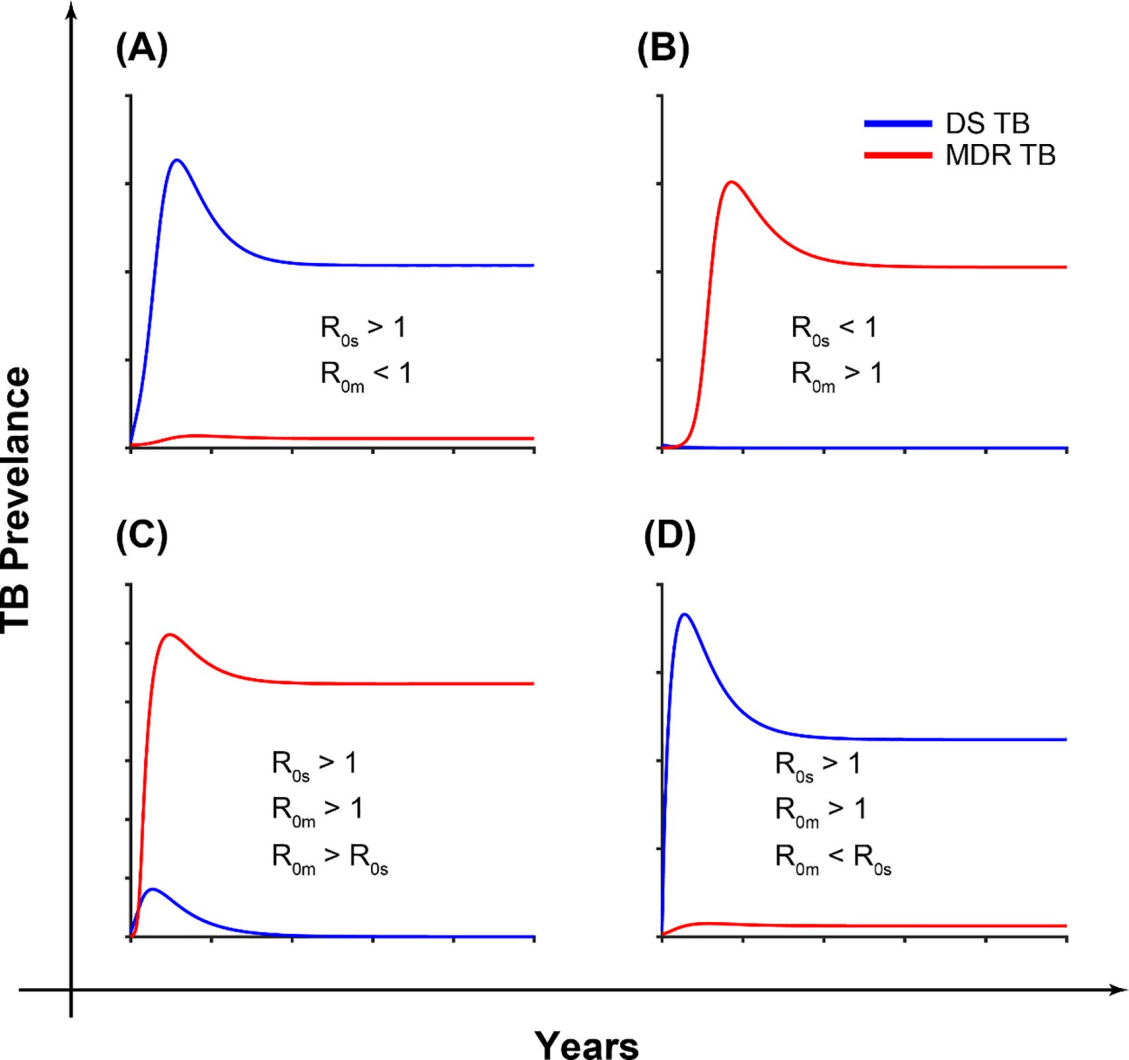
The impacts of the strain specific basic reproduction number on the long-time dynamics of the TB model (2).

For the two strain TB model (2), the basic reproduction number is

$$R_{0}=max(R_{0s},R_{0m}),$$ (3)

where $R_{0s}=\frac{N\alpha_{s}\beta_{s}}{\left(\alpha_{s}+\delta_{s}+\mu )(\omega_{s}+\phi_{s}+\tau_{s}+\mu \right)}$ and $R_{0m}=\frac{N\alpha_{m}\beta_{m}}{\left(\alpha_{m}+\delta_{m}+\mu )(\omega_{m}+\phi_{m}+\tau_{m}+\mu \right)}$. The strain-specific reproduction numbers R_0s_ and R_0m_ determine whether a specific strain will persist or die out in relation to the other strain. Both strains die out when both R_0m_<1 and R_0s_<1. However, the MDR strain will persist in the community even if R_0m_<1 and R_0s_>1 as the resistant strain is fuelled in two ways: transmission and amplification of the DS strain (Fig 3A). If R_0m_>1 and R_0s_<1, the DS strain dies out whilst the MDR strain persists in the community (Fig 3B). Similarly, the DS strain dies out if both strain specific basic reproduction number are greater than one and the R_0m_>R_0s_ (Fig 3C). Otherwise, both are sustained (Fig 3D).

With clear understanding of the dynamics of model (2), we fitted all the five models described in the model calibration section to the DS and MDR TB incidence data in Bangladesh to estimate the rate of amplification and the strain specific basic reproduction numbers. According to the AIC metric, we found that Model 2 captures the drug-sensitive TB data better and Model 3 captures the drug-resistant TB data better. See Table 2 for the fitting results of Model 2 and 3. While Model 1 is a good fit for the DS TB incidence data, it was worse for the MDR TB incidence. Model 4 did well for drug-sensitive TB but failed to capture the drug-resistant TB model well, while Model 5 gave the same fitting results as Model 3. See the section S.1 in S1 File for the fitting results of Model 1 and 4.

**Table 2:** Fitting results.

Parameter	Est.	95% CI
**Model 2**		
β_s_ = β_m_	1.54×10^−8^	(1.44×10^−8^, 1.64×10^−8^)
τ_s_ = τ_m_	0.513	(0.491, 0.535)
ρ	0.0005	(0, 0.008)
ρτ_s_	0.0003	(0, 0.0043)
	**DS**	**MDR**
AIC	369.11	361.10
**Model 3 and Model 5**		
β_s_	1.54×10^−8^	(1.5×10^−8^, 1.67×10^−8^)
β_m_	5.9×10^−9^	(4.2×10^−9^, 7.6×10^−9^)
τ_s_	0.470	(0.229,0.711)
τ_m_	0.470	(0.179,0.761)
P	0.024	(0.017,0.03)
	**DS**	**MDR**
AIC	421.74	353.38

**Fig 4:**
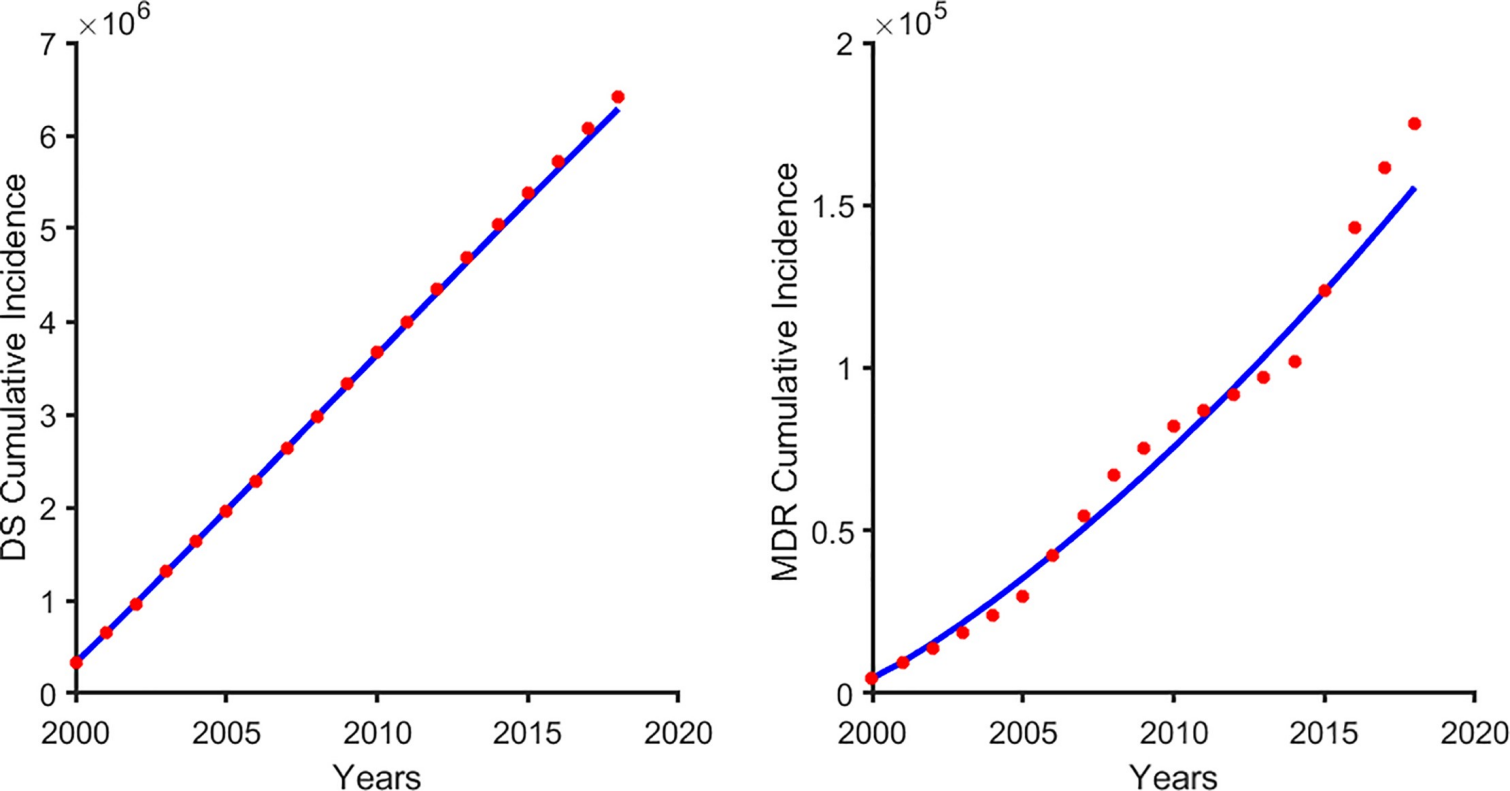
A fit of model 3 to the Bangladesh TB cumulative incidence: (A) drug- susceptible (DS) TB and (B) multi-drug resistant (MDR) TB.

Both Models 2 and 3 can explain the dynamics of DS and MDR TB in Bangladesh. For instance, we expect the same treatment rate for DS and MDR TB and, assuming the same average number of contacts between DS and MDR TB, then we will expect β_s_>β_m_ due to the fitness cost associated with drug-resistance (as postulated by Model 3) [33–35]. Similar conclusions can be reached from the estimates from Model 2. Model 2 (Drug-failure and equal transmission rates between DS and MDR TB) captures the DS TB better than Model 3 (Drug-failure and unequal transmission rates between DS and MDR TB) but since our focus is on MDR TB, we chose model 3 which captures MDR TB better and did not do worse for DS TB (see Fig 4) for our further analysis. For Model 2, the estimated strain specific basic reproduction numbers are R_0s_ = 1.1 and R_0m_ = 1.34 suggesting MDR TB will take over in the long run (Fig 3B), and for Model 3, R_0s_ = 1.14 and R_0m_ = 0.54, which means that DS TB will dominate and MDR TB will persist (Fig 3A). For Model 3, the amplification rate was 0.011, which is an average of 90 days between conversions from DS TB to MDR TB.

## 3.3 Sensitivity analysis of model parameters

We found β_i_ (the transmission rate), τ_i_ (the treatment rate) and ρ (the amplification rate) are important for the epidemiology of TB in Bangladesh. However, there are other key parameters that influence the transmission of DS and MDR TB. The understanding of these key parameters may also provide alternative intervention paradigms for TB control. As demonstrated in the previous sections, the scale and severity of TB transmission are directly associated with the basic reproduction numbers R_0s_ and R_0m_. Here, we estimated the sensitivity indices of the reproduction numbers R_0s_ and R_0m_ to the model parameters.

The indices express how vital each parameter is to R_0s_ and R_0m_, and in turn, to TB transmission dynamics, and allow us to identify which areas should be targeted by intervention policies. Here, we further computed partial rank correlation coefficients (PRCCs) which is a global sensitivity analysis technique using Latin Hypercube Sampling (LHS) to study the effects of other parameters that are not present in the basic reproduction number on the TB dynamics. Specifically, each parameter is assumed to be uniformly distributed and we performed 1,000,000 simulations of the TB model. Here, the model outputs are both basic reproduction numbers namely R_0s_ and R_0m_ as well as the equilibrium DS TB prevalence (I_s_), MDR TB prevalence (I_m_) and total TB prevalence (I_s_+I_m_). Positive (negative) PRCC values refer to a positive (negative) correlation of the model parameter and model outcome. A positive (negative) correlation suggests that a positive (negative) variation in the parameter will increase (decrease) the model outcome. The bigger (smaller) the absolute value of the PRCC, the greater (lesser) the correlation of the parameter with the model outcome.

**Fig 5:**
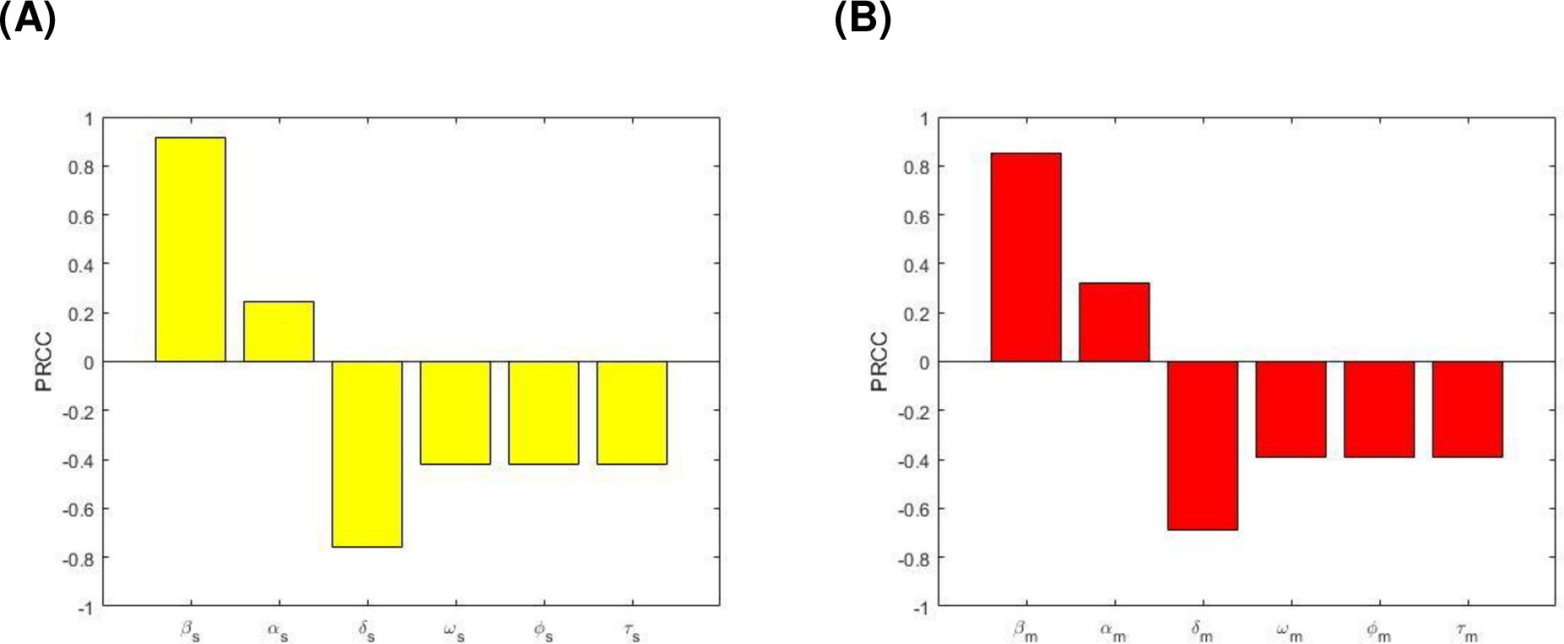
PRCC values depicting the sensitivities of the model output: (A) R_0s_ with respect to the estimated parameters β_s_, α_s_, δ_s_, ω_s_, ϕ_s_, and τ_s_, and (B) R_0m_ with respect to the estimated parameters β_m_, α_m_, δ_m_, ω_m_, ϕ_m_, and τ_m_.

Fig 5 shows the correlation between R_0s_ and R_0m_, and the corresponding model parameters. Parameters β_s_ and β_m_ have positive PRCC values, implying that a positive change of these parameters will increase the basic reproduction numbers R_0s_ and R_0m_ respectively. In contrast, parameters ω_s_, ϕ_s_, and τ_s_ as well as ω_m_, ϕ_m_, and τ_m_ have negative PRCC values, which implies that raising these parameters will consequently decrease R_0s_ and R_0m_.

**Fig 6:**
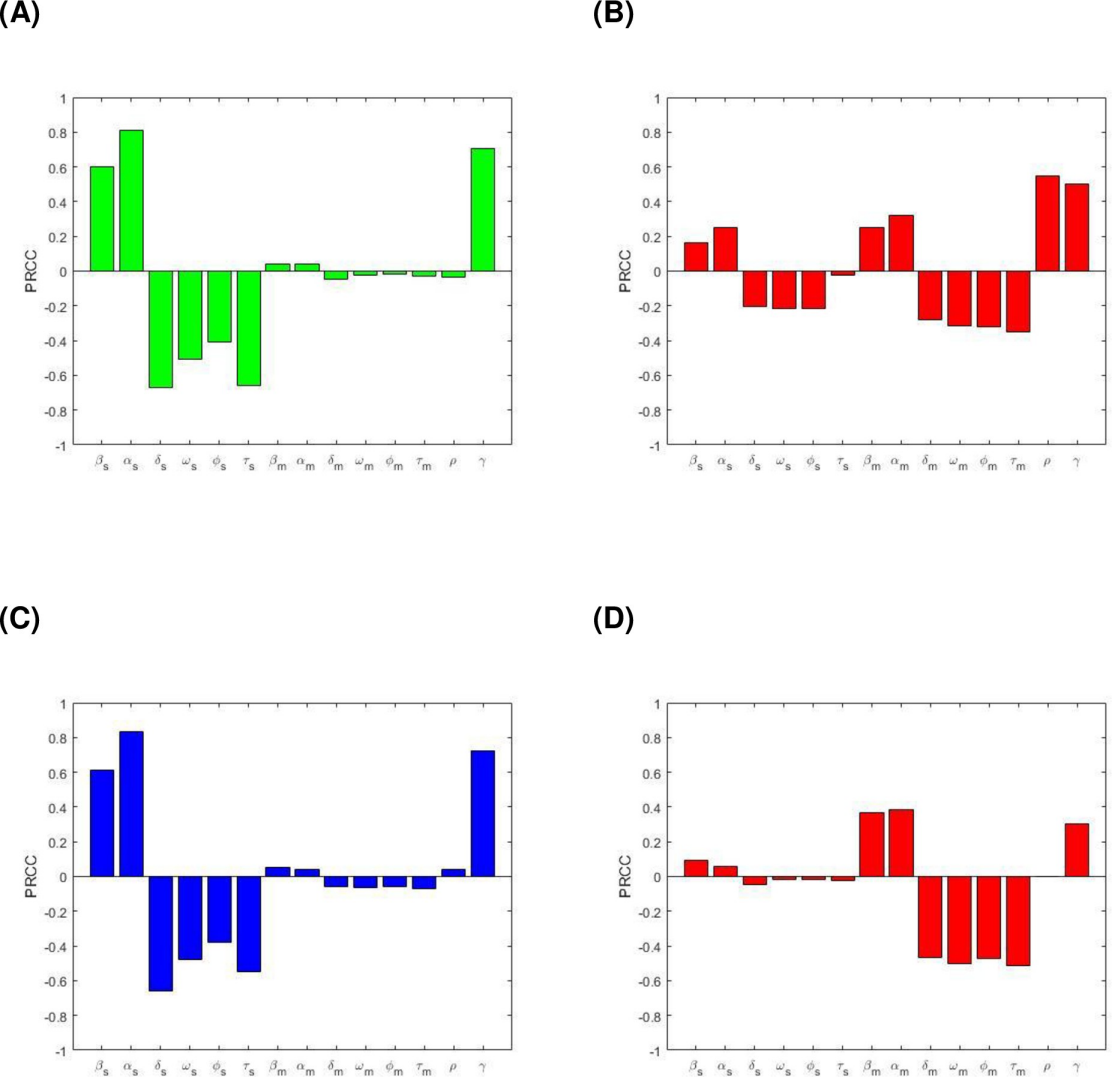
PRCC values depicting the sensitivities of the model output: (A) I_s_ with respect to the estimated parameters β_s_, α_s_, ω_s_, δ_s_, ϕ_s_, τ_s_, β_m_, α_m_, ω_m_, δ_m_, ϕ_m_, τ_m_ and ρ, (B) I_m_ with respect to the estimated parameters β_s_, α_s_, ω_s_, δ_s_, ϕ_s_, τ_s_, β_m_, α_m_, ω_m_, δ_m_, ϕ_m_, τ_m_ and ρ, (C) (I_s_+I_m_) with respect to the estimated parameters β_s_, α_s_, ω_s_, δ_s_, ϕ_s_, τ_s_, β_m_, α_m_, ω_m_, δ_m_, ϕ_m_, τ_m_ and ρ, when R_0s_>max[R_0m_, 1], and (D) I_m_ with respect to the estimated parameters β_s_, α_s_, ω_s_, δ_s_, ϕ_s_, τ_s_, β_m_, α_m_, ω_m_, δ_m_, ϕ_m_, τ_m_ and ρ, when R_0m_>R_0s_ and R_0m_>1.

Fig 6(A) shows a positive correlation between DS active TB prevalence (I_s_) and the model parameters β_s_, α_s_, ω_s_, ϕ_s_, τ_s_, β_m_, α_m_, ω_m_, ϕ_m_, τ_m_, ρ and γ, for R_0s_>max [R_0m_,1]. This implies that a positive change of these parameters will increase the number of people with DS active TB. In contract, parameters δ_s_, ω_s_, ϕ_s_, τ_s_, δ_m_, ω_m_, ϕ_m_,τ_m_ and ρ have a negative correlation with I_s_. Fig 6(B) represents the correlation between the MDR TB prevalence (I_m_) and corresponding model parameters β_s_, α_s_, ω_s_, ϕ_s_, τ_s_, β_m_, α_m_, ω_m_, ϕ_m_, τ_m_, ρ and γ for R_0s_>max [R_0m_,1]. The parameters β_s_, α_s_, β_m_, α_m_, ρ and γ have positive PRCC values, while the following parameters δ_s_, ω_s_, ϕ_s_, τ_s_, δ_m_, ω_m_, ϕ_m_ and τ_m_ have negative PRCC values. Fig 6(C) represents the correlation between total TB prevalence (I_s_+I_m_) and the corresponding model parameters when R_0s_>max [R_0m_,1]. We observed positive correlation with β_s_, α_s_, β_m_, α_m_, ρ and γ, implying an increase in the total TB prevalence with an increase in these parameter values. However, the parameters δ_s_, ω_s_, ϕ_s_, τ_s_, δ_m_, ω_m_, ϕ_m_ and τ_m_ have a negative correlation with total TB prevalence, which means increasing these parameters values will consequently decrease the total TB prevalence. Fig 6(D) represents the correlation between the MDR TB prevalence and corresponding model parameters β_s_, α_s_, ω_s_, ϕ_s_, τ_s_, β_m_, α_m_, ω_m_, ϕ_m_, τ_m_, ρ and γ when R_0m_>R_0s_ and R_0m_>1. Parameters β_s_, α_s_, β_m_, α_m_, ρ (small values not shown) and γ have positive PRCC values and parameters δ_s_, ω_s_, ϕ_s_, τ_s_, δ_m_, ω_m_, ϕ_m_ and τ_m_ have negative PRCC values.

From the explicit formula for R_0s_ and R_0m_, the analytical expression for the sensitivity indices can be derived applying the method in [36] to each of the parameters, e.g.

$$\Upsilon_{\beta_{s}}^{R_{0s}}=\frac{{\partial R}_{0s}}{\partial \beta_{s}}\times \frac{\beta_{s}}{R_{0s}}.$$ (4)

Now using the parameter values in Tables 1 and 2, we have the following results (Table 3).

**Table 3:** Sensitivity indices to parameters for the model (2).

Parameter	Sensitivity index (R_0s_)	Parameter	Sensitivity index (R_0m_)
β_s_	+ 1.000	β_m_	+1.000
α_s_	+0. 513	α_m_	+0.513
δ_s_	-0.453	δ_m_	-0.453
ω_s_	- 0.251	ω_m_	- 0.123
ϕ_s_	- 0.324	ϕ_m_	- 0.379
τ_s_	-0.413	τ_m_	- 0.484

In the sensitivity indices of R_0s_ and R_0m_, the most sensitive parameter is the effective contact rate of DS TB, β_s_ and MDR TB, β_m_. Other significant parameters are activation rates (α_s_ and α_m_). The least sensitive parameters are the recovery rates ω_s_ and ω_m_. Hence, increasing (or decreasing) the effective contact rates, β_s_ and β_m_ of DS TB and MDR TB by 100%, increases (or decreases) the reproduction numbers R_0s_ and R_0m_ by 100%. Similarly, increasing (or decreasing) the recovery rates ω_s_ and ω_m_ by 100% decreases (or increases) R_0s_ and R_0m_, by 25.1% and 12.3% respectively.

### 3.4 Optimal control strategies and cost-effectiveness analysis

We incorporated the earlier defined control strategies in the Bangladesh TB model (see Eq (4) below) and from it derived alternative measures to reduce the burden of TB. Our goal is to maximize the total number of active TB cases averted (TATBA)

$$\frac{dS}{dt}=\mu N-(1-u_{1}(t))\beta_{s}I_{s}S-{(1-u_{1}(t))\beta }_{m}I_{m}S-\mu S+\gamma R+\varphi_{s}I_{s}+\varphi_{m}I_{m},$$

$$\frac{dL_{s}}{dt}={(1-u_{1}(t))\beta }_{s}I_{s}S-(\alpha_{s}+{(\delta }_{s}+u_{2}(t)\tau_{1})+\mu )L_{s},$$

$$\frac{dI_{s}}{dt}=\alpha_{s}L_{s}-(\omega_{s}+(1+u_{4}(t))\tau_{s}+\phi_{s}+\mu )I_{s},$$

$$\frac{dL_{m}}{dt}=(1-u_{1}(t))\beta_{m}I_{m}S-(\alpha_{m}+{(\delta }_{m}+u_{2}(t)\tau_{2})+\mu )L_{m},$$

$$\begin{array}{l} \frac{dI_{m}}{dt}=\alpha_{m}L_{m}+\left(\rho (1-u_{3}(t))\right)(1+u_{4}(t))\tau_{s}I_{s}-(\omega_{m}+\tau_{m}(1+u_{4}(t))+\phi_{m}+\mu )I_{m},\frac{dR}{dt}\\ =(1-\rho (1-u_{3}(t)))(1+u_{4}(t))\tau_{s}I_{s}+\tau_{m}(1+u_{4}(t))I_{m}+\omega_{s}I_{s}+\omega_{m}I_{m}+\\ {(\delta }_{s}+u_{2}(t)\tau_{1})L_{s}+(\delta_{m}+u_{2}(t)\tau_{2})L_{m}-\gamma R-\mu R. \end{array}$$ (5)

From Eq (5), τ_1_ and τ_2_ are the treatment rates of the DS and MDR latent TB. The “do-nothing-more” control is the baseline control with zero additional cost and is used as the reference to calculate the total number of active TB infections averted. The objective of the optimal control strategy is to minimize the cost of reducing the number of latent DS and MDR TB (L_s_ and L_m_), and infectious individuals (I_s_ and I_m_). The controls range between 0 and 1, and when u_1_(t), u_2_(t), u_3_(t), u_4_(t) = 0, that is the “do-nothing-more” control, while u_1_(t), u_2_(t), u_3_(t), u_4_(t) = 1 refers to maximum effort of the control policy being implemented. We formulated four different control strategies with different alternatives and determined the cost-effectiveness of these strategies.

#### 3.4.1 Single control strategy

For this control strategy, we have four alternatives:

- u_1_(t), only distancing control
- u_2_(t), only latent case finding
- u_3_(t), only case holding
- u_4_(t), only active case finding.

For each of these alternatives, the objective functional is of the form:

$$minimize:\;J(u_{i})=\int_{t_{0}}^{t_{f}}\left(A_{1}L_{s}+{A_{2}I}_{s}{+A_{3}L_{m}+A_{4}I}_{m}+\frac{B_{i}}{2}u_{i}^{2}\right)dt,$$ (6)

Here, the total cost on a finite time horizon [t_0_, t_f_] (where initial time t_0_ = 0, final time t_f_ = 20 year period) consists of the cost induced by the DS and MDR TB cases themselves and the cost induced by the efforts of the four different types of control [37, 38] strategies including distancing, latent case finding, case holding, and active case finding. We split the cost induced by latent DS and MDR TB, $\int_{t_{0}}^{t_{f}}A_{1}L_{s}(t)dt$ and $\int_{t_{0}}^{t_{f}}A_{3}L_{m}(t)dt$, proportional to the number of latently infected individuals of DS and MDR TB respectively. Further, the cost induced by active DS and MDR TB cases, $\int_{t_{0}}^{t_{f}}A_{2}I_{s}(t)dt$ and $\int_{t_{0}}^{t_{f}}A_{4}I_{m}(t)dt$, proportional to the number of actively infected individuals of DS and MDR TB respectively. Here, we consider the biquadratic form in the four control strategies to represent increased expense of these strategies. The cost involved in the distancing, latent case finding, case holding, and active case finding strategies is taken as $\int_{t_{0}}^{t_{f}}\frac{B_{1}}{2}u_{1}^{2}(t)dt,\int_{t_{0}}^{t_{f}}\frac{B_{2}}{2}u_{2}^{2}(t)dt,\int_{t_{0}}^{t_{f}}\frac{B_{3}}{2}u_{3}^{2}(t)dt,$ and $\int_{t_{0}}^{t_{f}}\frac{B_{4}}{2}u_{4}^{2}(t)dt$, respectively. It is assumed that the cost of each control strategy is nonlinear and takes a quadratic form, which is found to be consistent with previous works [37].

The coefficients A_i_ are the cost of diagnosing and treating latently infected and infectious individuals. Here, we consider A_1_ = US$18.40 per latent DS TB case and A_2_ = US$119.58 per active DS TB case per year. Conversely, since MDR TB treatment is far more expensive, we take A_3_ = US$4055 per latent MDR TB case and A_4_ = US$3955 per active MDR TB case per year [39].

The coefficients B_i_ (i = 1,2,3,4) represent the weight constants associated with the relative costs of implementing the respective control strategies. The distancing programme involves education, media coverage, and encouraging reduction of contacts with infectious TB patients. In Bangladesh, the cost of telecast for 90 minutes is US$2916 and the cost per hospital bed per day is around US$19.29 [40–42]. There are approximately 97,800 hospital beds in Bangladesh [43] and we assume that around 432 beds are involved for TB transmission control protecting susceptible individuals from infected individuals. Thus for a year run of the distancing control programme the total cost is around B_1_ = US$4,103,583. The unit cost per diagnosis of TB is US$3804 [40] and there is an average of 1744 health workers in Bangladesh [44]. Hence, we have B_2_ = B_4_ = US$6,634,176. For the case holding, we assume 1000 health workers are recruited for this purpose. The unit cost per health worker is US$3607 [40] and hence B_3_ = $3,607,000.

**Fig 7:**
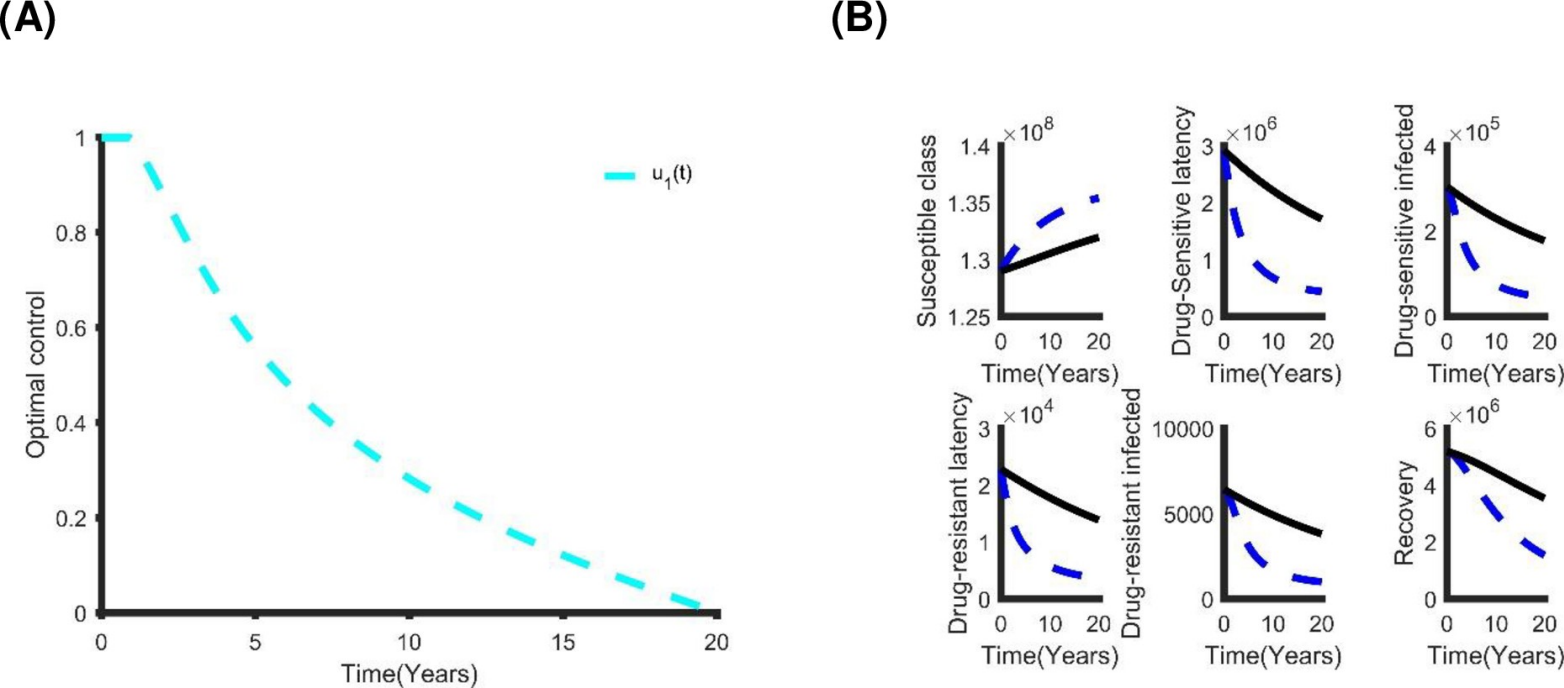
**5**The single optimal control strategy: (A) The optimal distancing control strategy. (B) The benefits of using only distancing control strategy.

The section S.2 in S1 File shows the optimal characterization of the control problems. Fig 7 and section S.2 and S4 Fig in S1 File show the optimal solutions of the single control strategy. For each of these alternatives, the application of each control leads to a reduction in TB prevalence. However, we used cost-effectiveness analysis to determine the most cost-effective strategy to use in the control of TB in Bangladesh. This is performed by associating the differences among the costs and outcomes of each intervention; obtained by estimating the incremental cost-effective ratio (ICER) which is defined as the extra cost per additional intervention outcome. Incrementally, when analyzing two or more competing intervention policies, one intervention is associated with the next less effective option. The ICER numerator is given by the total difference in intervention costs, active TB cases averted costs and averted productivity losses if applicable, between each scenario and baseline. The ICER denominator is the total number of active TB cases averted. The ICER is obtained by the following formula:

$$ICER_{i}=\frac{{TC}_{i}}{{TATBA}_{i}}$$ (7)

where, i = list of control strategies

From Table 4, the distancing strategy is less expensive and more effective than other alternatives. Hence, it is the preferable single control strategy. Alternatively, latent case finding is another good choice.

**Table 4:** Incremental cost-effective ratio of single control strategy.

Single Control	Total Cost (TC)	TATBA	ICER
Distancing	US$1.40×10^9^	3.09×10^6^	4.52×10^2^
Latent case finding	US$1.92×10^9^	2.25×10^6^	8.56×10^2^
Case holding	US$3.22×10^9^	1.47×10^1^	2.19×10^8^
Active case finding	US$2.86×10^9^	6.77×10^5^	4.22×10^3^

#### 3.4.2 Dual control strategy

In the dual implementation scenario we have six alternative strategies:

- u_1_(t) and u_2_(t): distancing and latent case finding
- u_1_(t) and u_3_(t): distancing and case holding
- u_1_(t) and u_4_(t): distancing and active case finding
- u_2_(t) and u_3_(t), latent case finding and case holding
- u_2_(t) and u_4_(t), latent case finding and active case finding
- u_3_(t) and u_4_(t), case holding and active case finding

**Fig 8:**
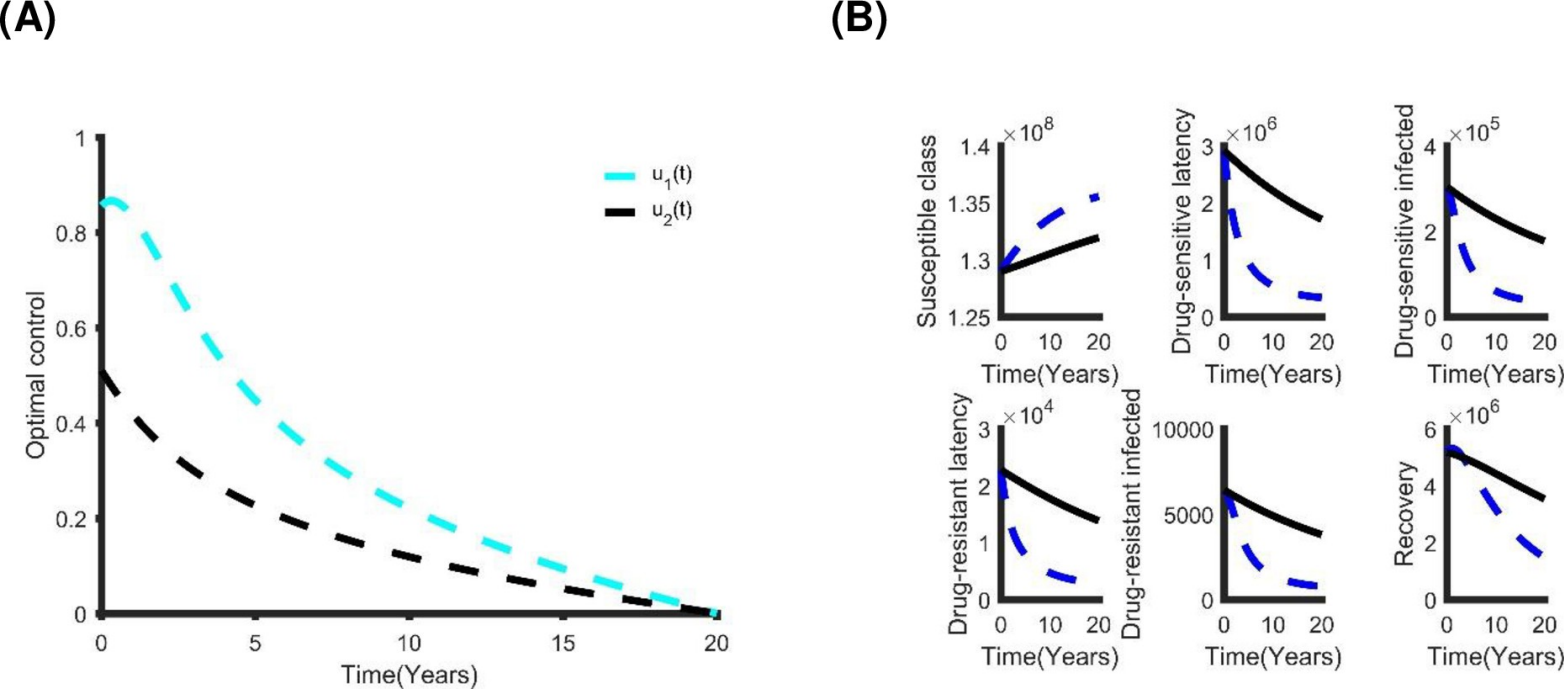
The double optimal control strategy: (A) The Distancing and latent case finding control strategy. (B) The benefits of using distancing and latent case finding control strategy.

The objective functional in this case is:

$$minimize:\;J(u_{i},u_{j})=\int_{t_{0}}^{t_{f}}\left(A_{1}L_{s}+A_{2}I_{s}{+A_{3}L_{m}+A_{4}I}_{m}+\frac{B_{i}}{2}u_{i}^{2}+\frac{B_{j}}{2}u_{j}^{2}\right)dt,$$ (8)

where the parameters in Eq (8) are as defined above. Each of the strategies resulted in decreasing the number of infected people at different levels of control (Fig 8 and section S.2, S5 Fig in S1 File). The cost-effectiveness analysis shows that a combination of the distancing and latent case finding is the best dual strategy (see Table 5).

**Table 5:** Incremental cost-effective ratio of coupled control strategy.

Dual Control	Total Cost (TC)	TATBA	ICER
Distancing and latent case finding	US$1.22×10^9^	3.40×10^6^	3.59×10^2^
Distancing and case holding	US$1.40×10^9^	3.09×10^6^	4.52×10^2^
Distancing and active case finding	US$1.40×10^9^	3.08×10^6^	4.53×10^2^
Latent case finding and case holding	US$1.92×10^9^	2.25×10^6^	8.55×10^2^
Latent and active case finding	US$1.82×10^9^	2.42×10^6^	7.54×10^2^
Case holding and active case finding	US$2.85×10^9^	6.77×10^5^	4.21×10^3^

#### 3.4.3 Triple control strategy

For the combination of three different control practices, we have four alternative strategies:

- u_1_(t), u_2_(t) and u_4_(t): distancing, latent case finding and active case finding
- u_1_(t), u_2_(t) and u_3_(t): distancing, latent case finding and case holding
- u_1_(t), u_3_(t) and u_4_(t): distancing, case holding and active case finding
- u_2_(t), u_3_(t) and u_4_(t): latent case finding, case holding and active case finding

Hence, the objective functional is

$$minimize:\;J(u_{i},u_{j},u_{k})=\int_{t_{0}}^{t_{f}}\left({A_{1}L}_{s}+{A_{2}I}_{s}{+A_{3}L_{m}+A_{4}I}_{m}+\frac{B_{i}}{2}u_{i}^{2}+\frac{B_{j}}{2}u_{j}^{2}+\frac{B_{k}}{2}u_{k}^{2}\right)dt,$$ (9)

**Fig 9:**
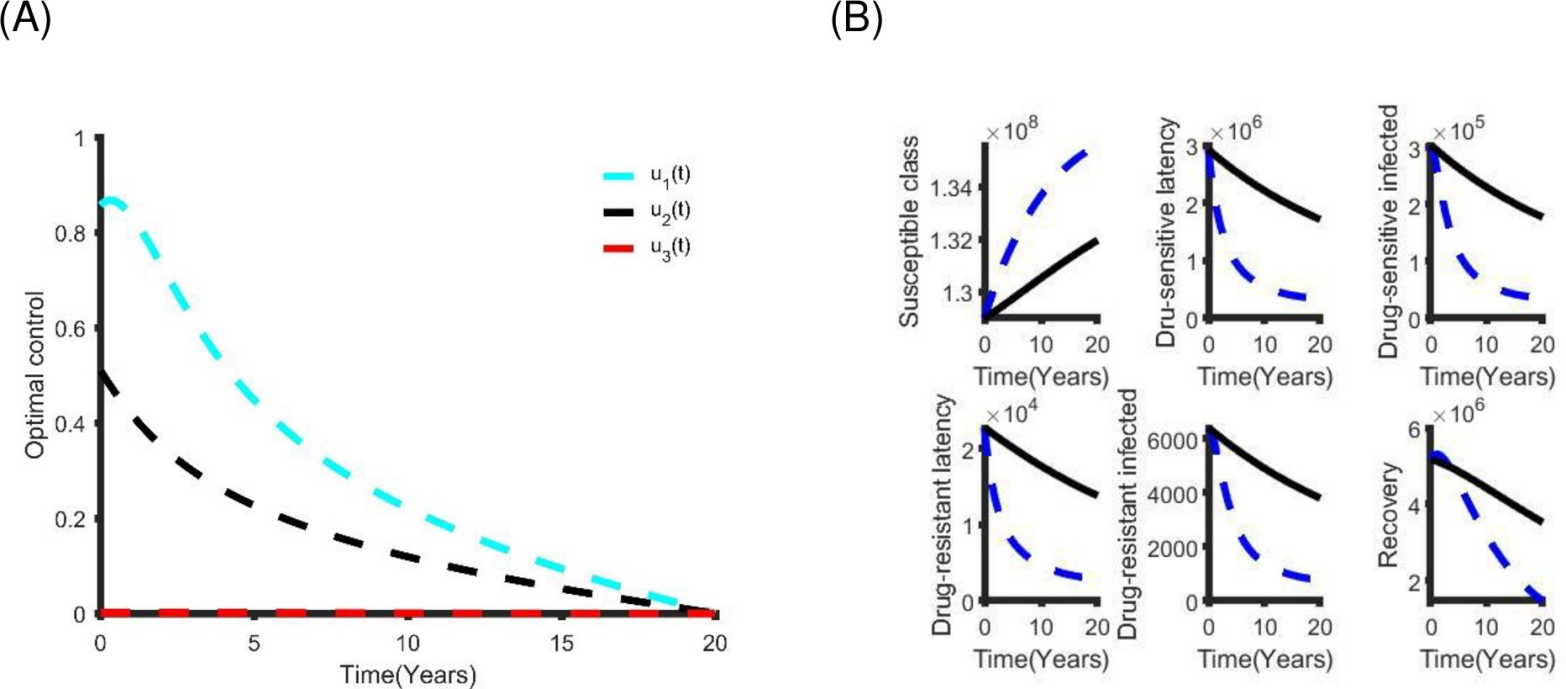
The triple optimal control strategy: (A) The distancing, latent case finding and case holding control strategy. (B) The benefits of using distancing, latent and active case finding control strategy.

As expected, each of the strategies resulted in decreasing the number of infected people at different levels of cost (Fig 9 and section S.2, S6 Fig in S1 File), and we used cost-effectiveness analysis to determine which of these strategies is the most cost-effective (Table 6). The combination of distancing, latent case finding and case holding is the best triple control strategy. Alternatively, distancing, latent case finding and active case finding also provides cost-effective results.

**Table 6:** Incremental cost-effectiveness ratio of each triple control strategy.

Triple Control	Total Cost (TC)	TATBA	ICER
u_1_(t), u_2_(t) and u_3_(t)	US$1.22×10^9^	3.40×10^6^	3.58×10^2^
u_1_(t), u_2_(t) and u_4_(t)	US$1.22×10^9^	3.39×10^6^	3.58×10^2^
u_1_(t), u_3_(t) and u_4_(t)	US$1.40×10^9^	3.08×10^6^	4.53×10^2^
u_2_(t), u_3_(t) and u_4_(t)	US$1.82×10^9^	2.42×10^6^	7.53×10^2^

#### 3.4.4. Quadruple control strategy

In this case, all four controls are used. The objective function is

$$minimize:\;J(u_{1},u_{2},u_{3},u_{4})=\int_{t_{0}}^{t_{f}}\left({A_{1}L}_{s}+{A_{2}I}_{s}{+A_{3}L}_{m}+A_{4}I_{m}+\frac{B_{1}}{2}u_{1}^{2}+\frac{B_{2}}{2}u_{2}^{2}+\frac{B_{3}}{2}u_{3}^{2}+\frac{B_{4}}{2}u_{4}^{2}\right)dt.$$ (10)

**Fig 10:**
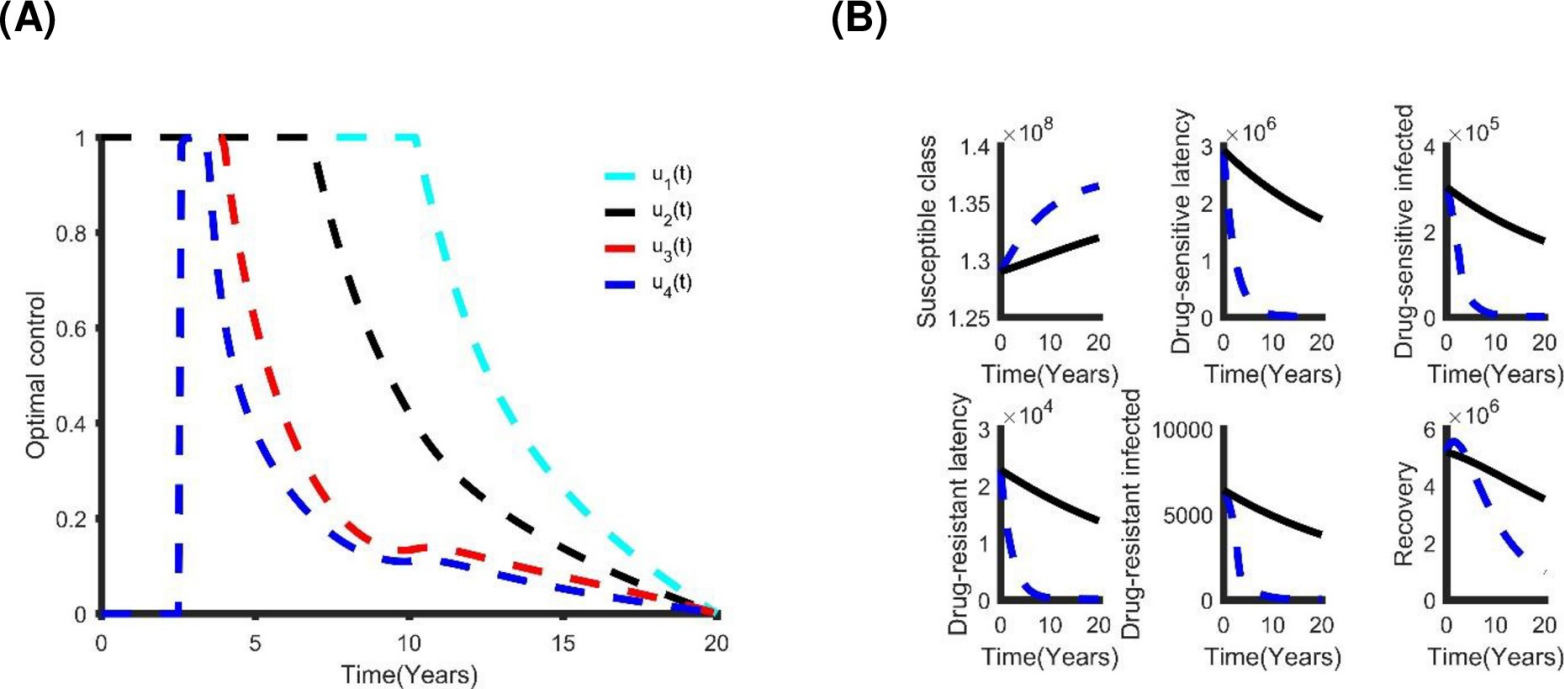
(A) The optimal quadruple control strategy; and (B) its effect on TB prevalence in Bangladesh.

Fig 10 shows the optimal controls and the benefits of this intervention method. The number of individuals with DS and MDR TB reduces to zero in less than 12 years of rolling out this policy. This outcome is similar to the triple control strategy but comes at a cost of US$5.87×10^8^ with a total of 4.46×10^6^ infections averted within 20 years. We compared all the control strategies with each other to determine which is the most cost-effective (Table 7). The quadruple control strategy (distancing, latent case finding, case holding and active case finding) is the best strategy. However, depending on availability of funding, other strategies in Table 5 can be considered.

**Table 7:** Selecting best control strategy.

Best control strategy	Total Cost (TC)	TATBA	ICER
u_1_(t), u_2_(t), u_3_(t) and u_4_(t)	US$5.87×10^8^	4.46×10^6^	1.32×10^2^
u_1_(t), u_2_(t) and u_4_(t)	US$1.22×10^9^	3.39×10^6^	3.58×10^2^
u_1_ and u_2_(t)	US$1.22×10^9^	3.40×10^6^	3.59×10^2^
u_1_(t)	US$1.40×10^9^	3.09×10^6^	4.52×10^2^

#### 3.4.5 Sensitivity analysis of our best optimal control strategies

**Fig 11:**
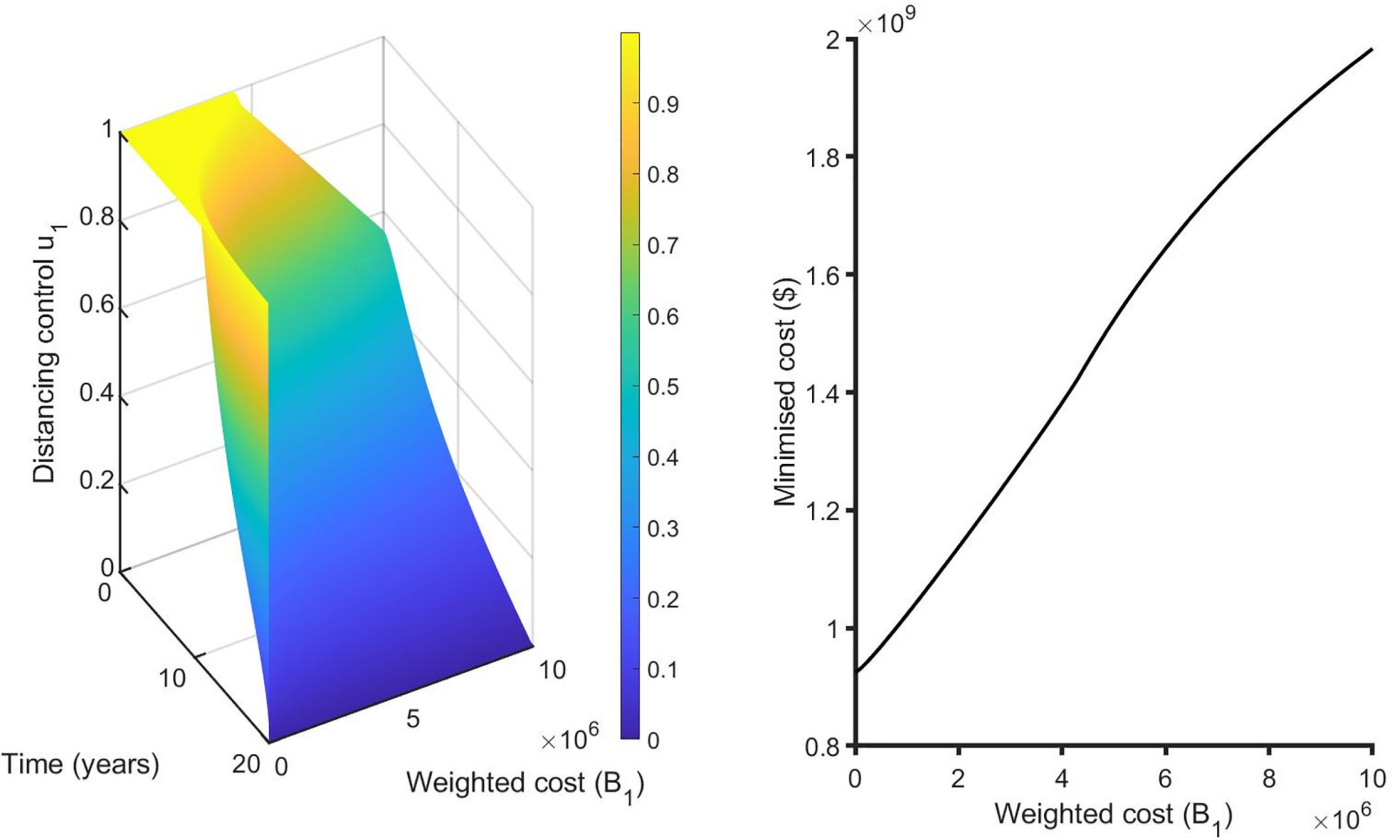
Effects of varying the weighted cost (B_1_) on the distancing control (u_1_) (left Fig) and the objective functional (right Fig).

**Fig 12:**
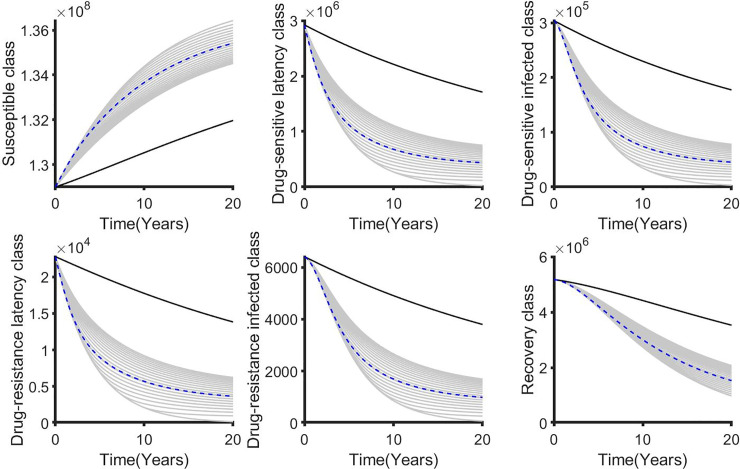
The corresponding state variables associated with varying the weighted cost (B_1_) while applying the distancing control (u_1_). The state variables with and without controls are plotted by gray/blue dotted and black lines respectively.

We performed a sensitivity analysis on the weighted costs associated with each control from our selected best strategies to determine how variability in the weighted costs affects our objective functional and the optimal control adopted. For the single control strategies, the distancing control u_1_(t) is the best strategy for this class. Hence, we vary B_1_ from 1 to 10^7^ with an equidistant step resulting in 1000 variates of B_1_. In general, if the weighted cost is smaller, the relative unit cost of using control is cheaper and the control is fully utilized for all the intervention period (Fig 11). Otherwise, the increase weighted cost penalizes the control and it’s proportionally applied to adjust for high cost. The corresponding state variables are shown in Fig 12.

**Fig 13:**
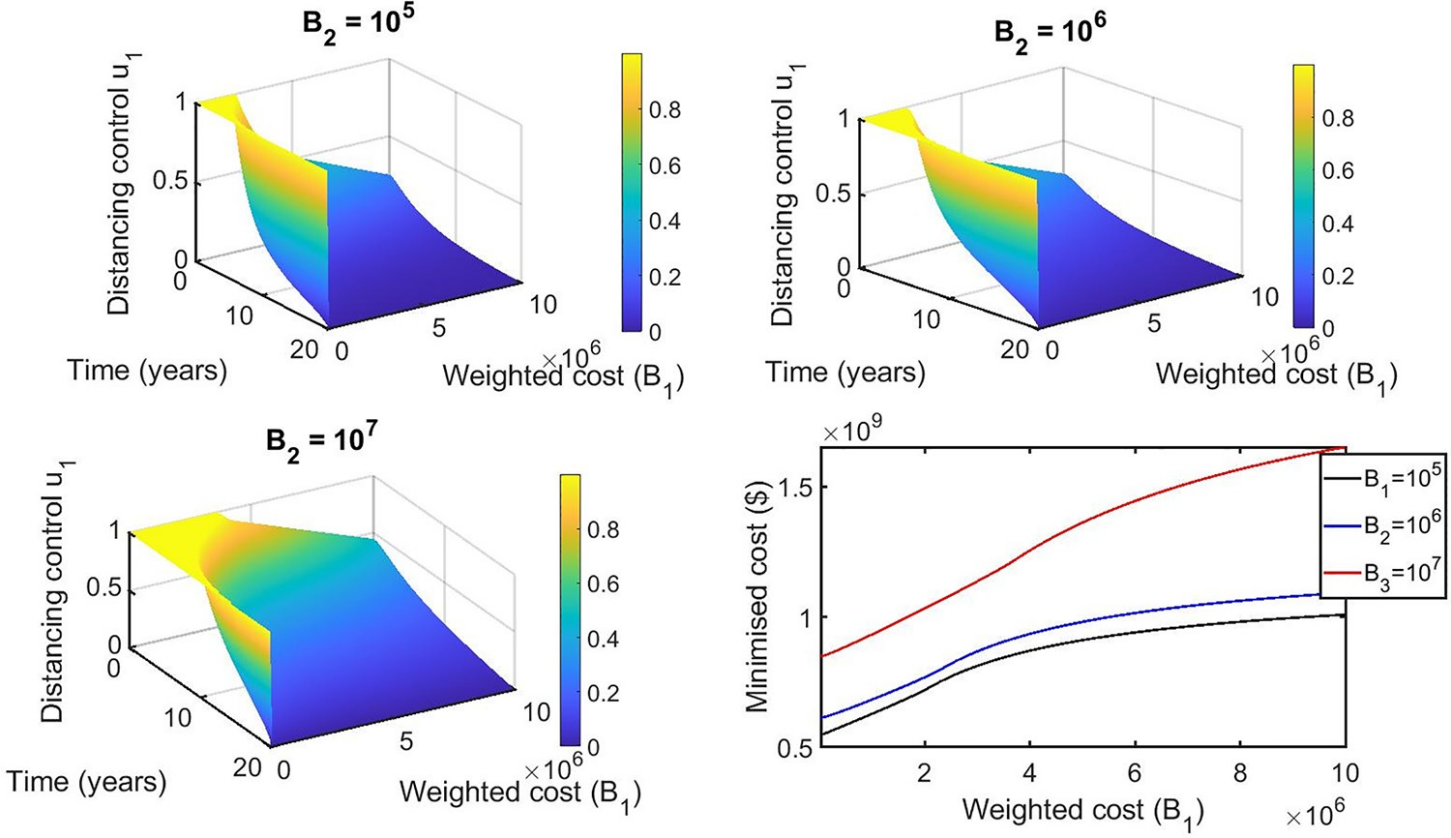
Combination of distancing control (u_1_) and latent case finding control (u_2_) strategy, and considering distancing control (u_1_) strategy as a function of time and weighted cost (B1). The weighted cost (B_2_) is set to the threshold values B_2_ = 10^5^ = 10^6^ = 10^7^.

**Fig 14:**
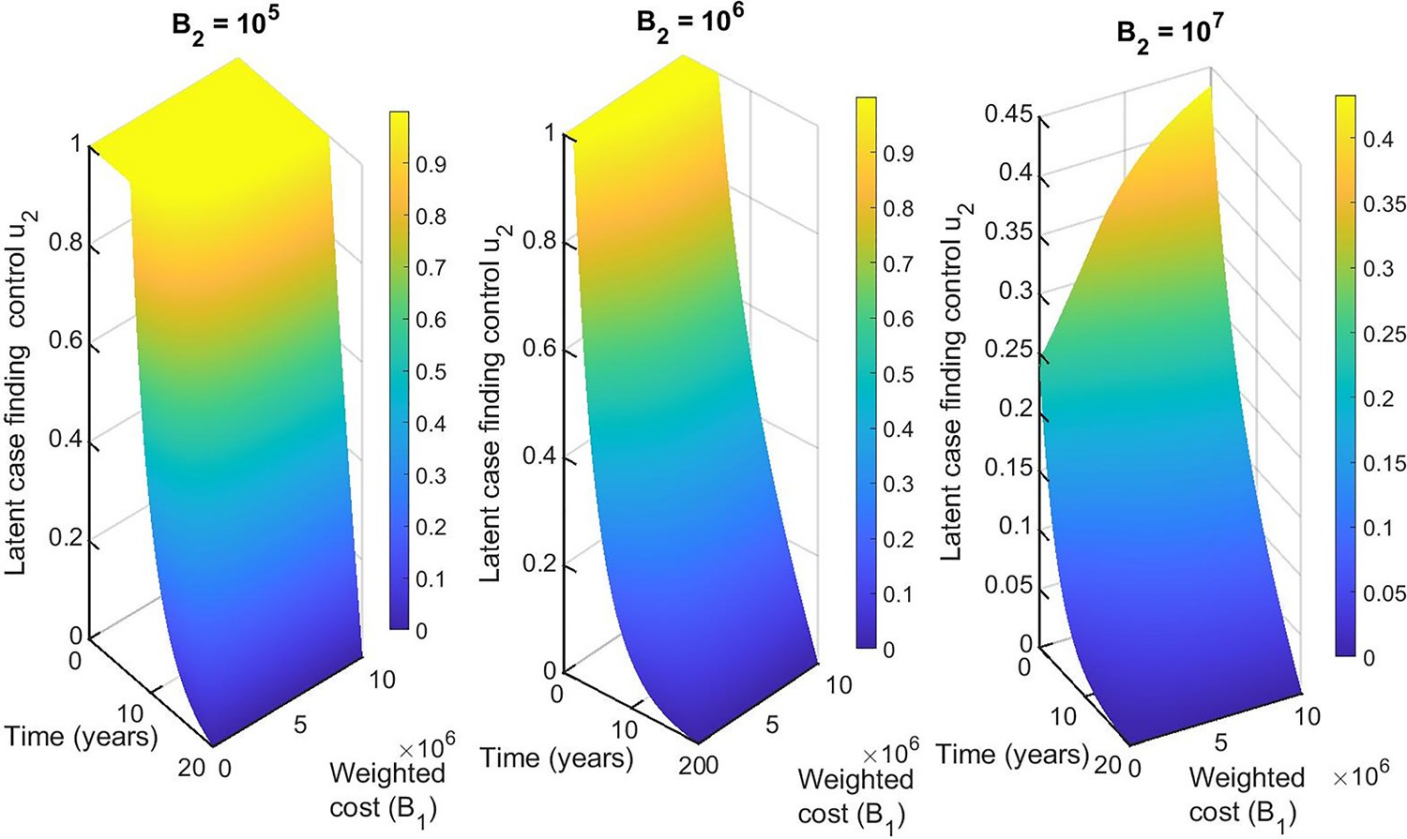
Combination of distancing control (u_1_) and latent case finding control (u_2_) strategy, and considering latent case finding control (u_2_) strategy as a function of time and weighted cost (B_1_). The weighted cost (B_2_) determined by three threshold values B_2_ = 10^5^ = 10^6^ = 10^7^.

**Fig 15:**
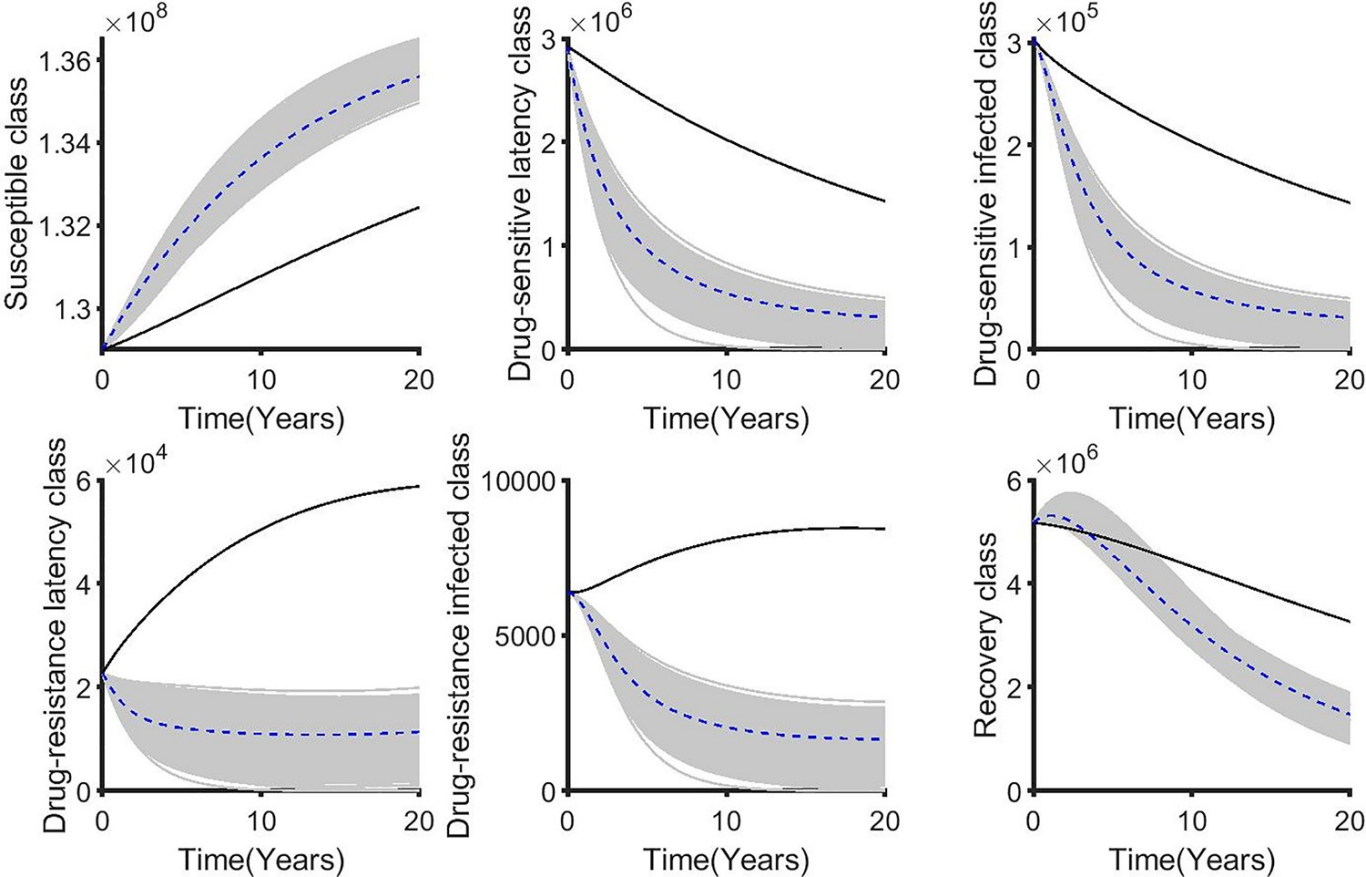
The corresponding state variables of the combination of distancing control (u_1_) and latent case finding control (u_2_) strategy and considering the weighted cost B_1_ is varied and B_2_ = 10^5^ = 10^6^ = 10^7^. The state variables with and without controls are plotted by grays/blue dotted and black lines respectively.

**Fig 16:**
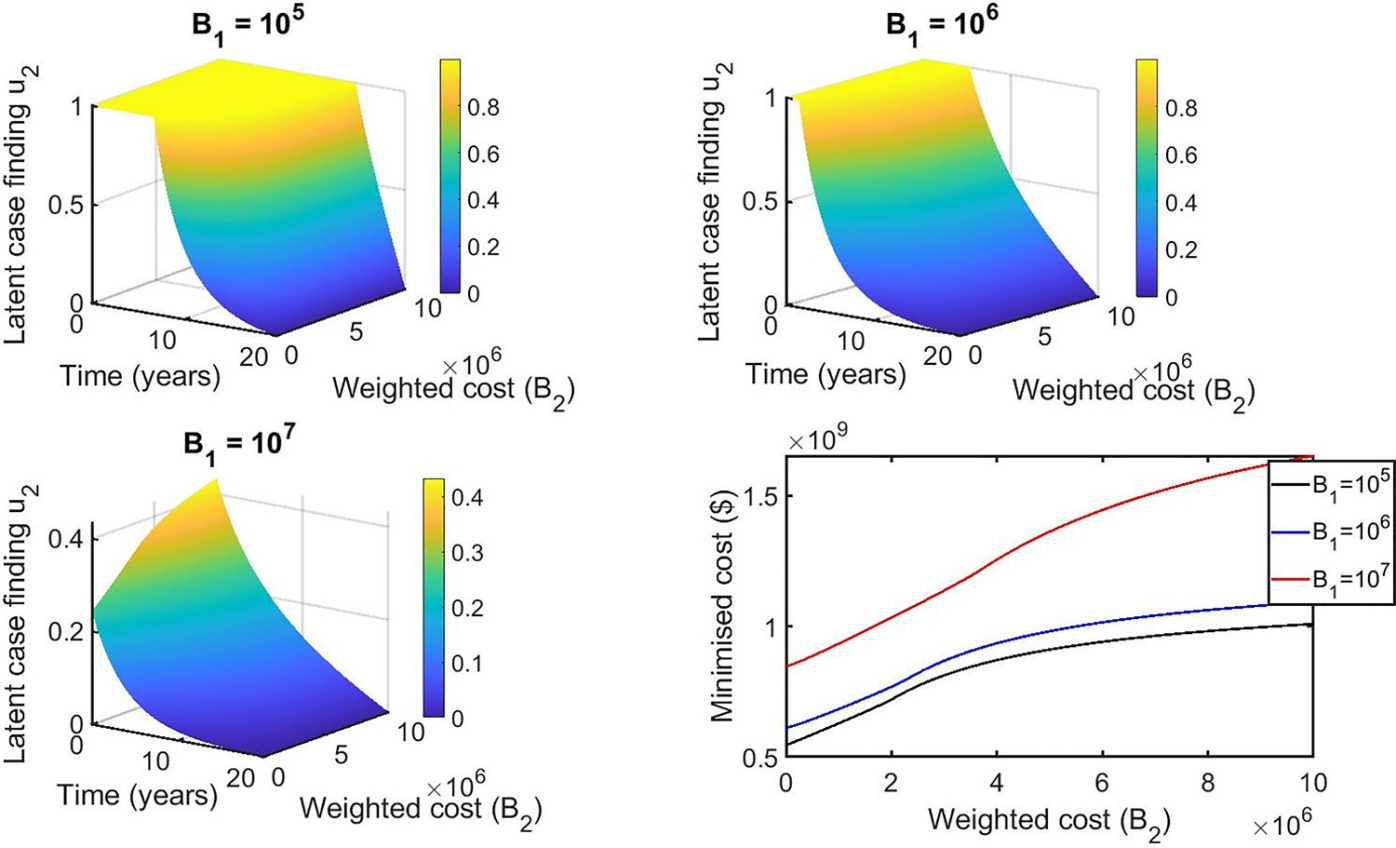
Combination of distancing control (u_1_) and latent case finding control (u_2_) strategy, and considering latent case finding (u_2_) strategy as a function of time and weighted cost (B_2_). The weighted cost (B_1_) determined by three threshold values B_1_ = 10^5^ = 10^6^ = 10^7^.

**Fig 17:**
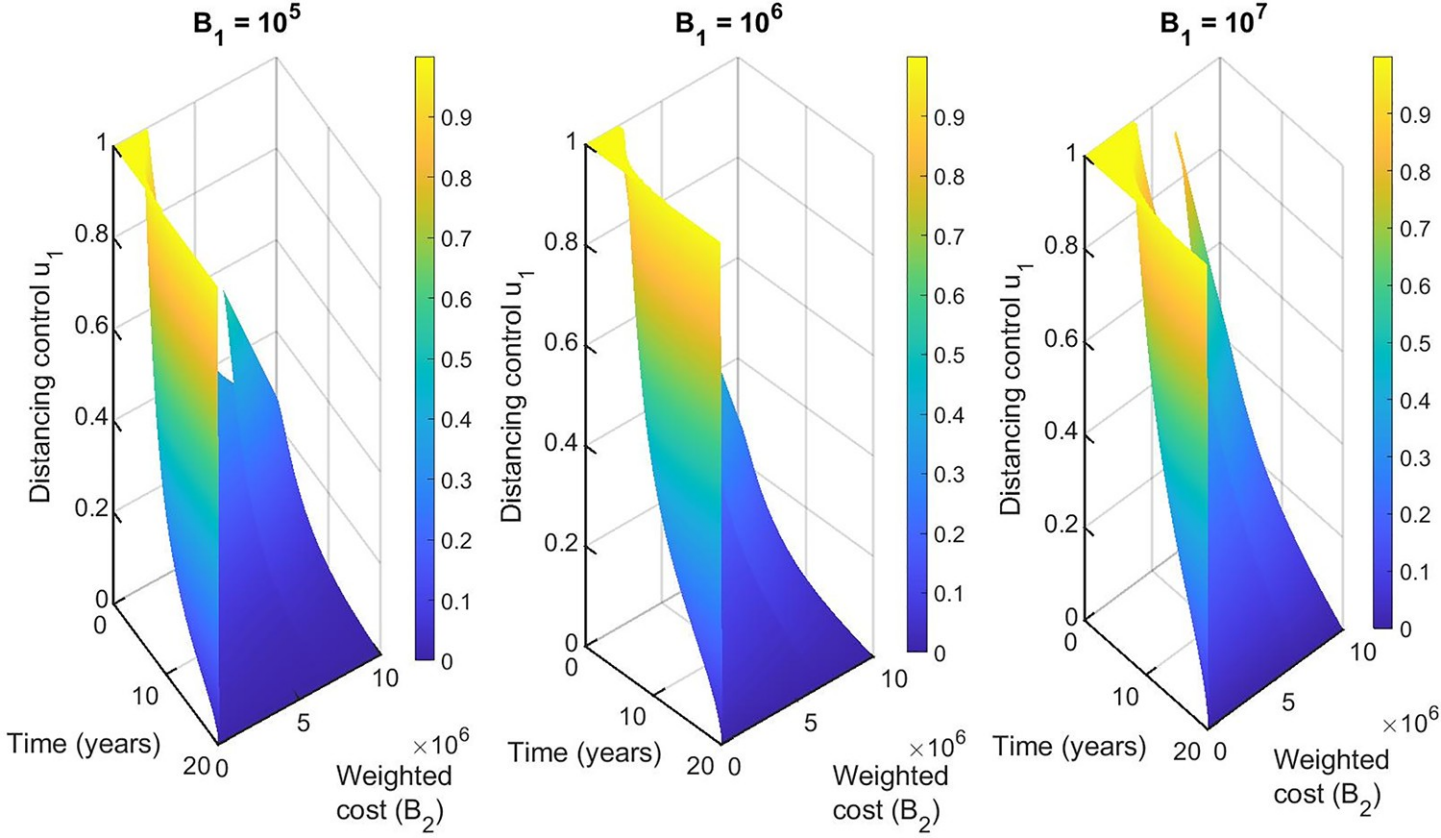
Combination of distancing control (u_1_) and latent case finding (u_2_) control strategy, and considering distancing control (u_1_) strategy as a function of time and weighted cost (B_2_). The weighted cost (B_1_) determined by three threshold values B_1_ = 10^5^ = 10^6^ = 10^7^.

**Fig 18:**
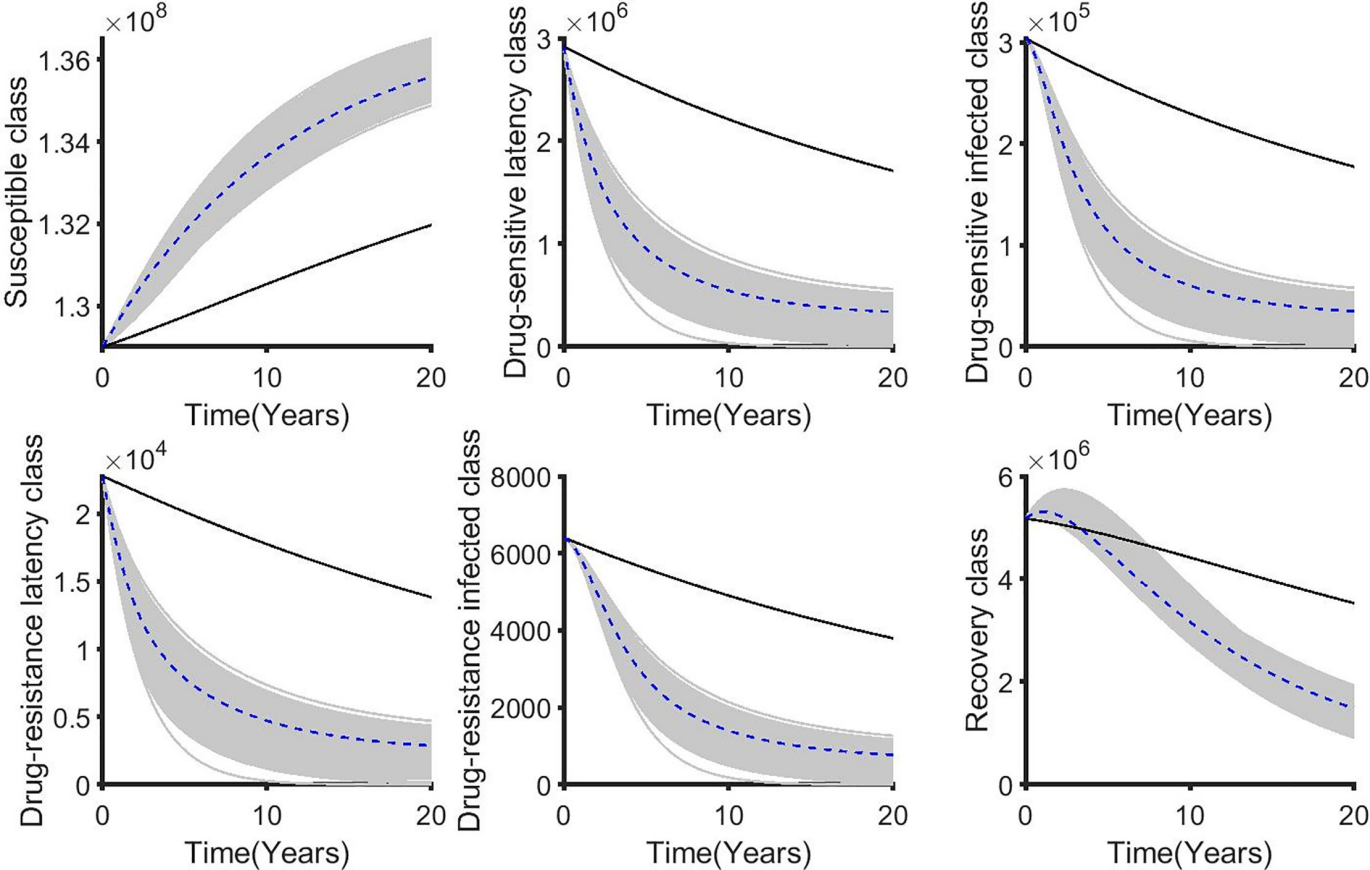
The corresponding state variables of the combination of distancing control (u_1_) and latent case finding control (u_2_) strategy and considering the weighted cost B_2_ is varied and B_1_ = 10^5^ = 10^6^ = 10^7^. The state variables with and without controls are plotted by grays/blue dotted and black lines respectively.

For the double control strategies, the combination of distancing u_1_(t) and latent case finding u_2_(t) is the best strategy for this class. Figs 13, 14, 15, 16 and 17 show the combination of optimal control strategies including distancing and latent case finding and their effects on the state variables. In Fig 13, we considered three threshold values for B_2_: B_2_ = 10^5^, 10^6^ and 10^7^, and varied the weighted cost B_1_. This increase in B_2_ shows little effect on the distancing control u_1_(t) (Fig 13) but strong effect on the latent case finding control u_2_(t) (Fig 14). In a similar way, when we fixed the weighted cost B_1_, both controls change, with reductions in the amount of control required at higher cost (see Figs 16 and 17). As expected higher weighted cost increases the cost of implementation of the controls (see the right lower quadrant of Figs 13 and 16), and the effects on the state variables are shown in Figs 15 and 18.

We further performed similar sensitivity of the weighted costs on both triple and quadruple control strategies (see section S.3 in S1 File).

## 4. Discussion and conclusion

TB (DS and MDR) is one of the most pressing public health problems in Bangladesh [1]. Overall, the transmission dynamics and epidemiology of TB (DS and MDR) in Bangladesh are poorly understood. Bangladesh’s government initiated various intervention programs to eliminate DS and MDR TB in the last decades. Although DS and MDR TB control in Bangladesh has significantly progressed–improved case finding, availability of free diagnostic and treatment services, the involvement of multiple partners, newer diagnostic facilities, sufficient human resources, adequate capacity, and guidelines–more effort is required. To reduce DS and MDR TB incidence, prevalence and prevent deaths from DS and MDR TB in Bangladesh, we need to identify the critical factors for developing TB (DS and MDR) disease, improve treatment effectiveness, and reduce failure of treatment in infectious individuals.

In this paper, we presented a two strain TB compartmental model with amplification to understand the transmission dynamics of DS and MDR TB in Bangladesh. We derived the basic reproduction number of each TB strain, and evaluated the role of the strain-specific reproduction number on the dynamics of DS and MDR TB. We proposed five different TB models and applied them to DS and MDR TB incidence data in Bangladesh. The model with unequal transmission DS and MDR TB transmission rates and same treatment rate captured the MDR TB dynamics the best. With these parameters estimated, we calculated the basic reproduction number of TB in Bangladesh and found it to be greater than one. However, the uncertainty around the parameter estimates could bring the basic reproduction number to below one. This is reflective of the effective reproduction number indicating whether control measures are effective or not. Nonetheless, the estimates help identify interventions that may be effective via sensitivity analysis of the associate parameters and suggests further intervention that can be achieved via optimal control strategies. Both were carried out in the study with transmission rates influencing the TB dynamics more than any other variable.

We adopted optimal control analysis via Pontryagin’s Maximal principle [45] and formulated the optimal strategies for controlling the DS and MDR TB epidemic in Bangladesh. Four different control strategies were considered (single, dual, triple and quadruple) from combinations of distancing, latent case finding, case holding and active case finding controls and were examined to measure their cost-effectiveness.

Among the four single-controls, the distancing control strategy is the most cost-effective. Latent case finding control appears to be more effective than active case finding. The least effective is the case holding control. Therefore, when only one control strategy is used, our results suggest that the Bangladesh government should improve distancing control interventions, reducing contact between infectious and susceptible people.

Within the six-dual-control strategies, combinations with distancing control performed best, and adding latent case finding control is the most cost-effective and more rapidly reduces DS and MDR TB compared to other dual control strategies. The active case finding control is more feasible than the case holding control in light of not only reducing the number of DS and MDR TB cases but also reducing control implementation duration. In view of the difficulty of implementing distancing measures which involves a high social cost, pharmaceutical control which includes latent case finding, active case finding and case holding should also be considered. We found that latent case finding and active case finding control as a dual control strategy is more cost-effective than the other dual pharmaceutical control strategies and rapidly reduces DS and MDR TB. Therefore, if two control strategies are considered, we recommended that distancing control should be included. If distancing control is implemented successfully, the Bangladesh government can achieve the WHO TB elimination goal with fewer pharmaceutical control processes. However, if distancing is infeasible, combined latent and active therapy is also worthwhile.

Considering the triple control strategy structure, distancing with latent case finding and case holding control is the most cost-effective. If distancing control is difficult to implement, it is suggested that pharmaceutical controls including latent case finding, case holding and active case finding can be used. From the analysis of all the control strategies, we found that the most cost-effective control is the quadruple control strategy, followed by the double control strategy, triple control and single control.

Optimal control strategies has been applied in other endemic settings to minimize the number of TB cases and the intervention implementation costs. Previous studies show that for the single control strategy, distancing control is the best strategy and for the double control strategy, distancing and latent case finding control is the best strategy to decrease the number of TB cases and intervention costs [37, 46], which is similar to our results. However, our study shows that for the four triple-control strategies, distancing, latent case finding and case holding is the best option, which is similar to [46] but dissimilar to [37]. We can speculate as to why active case finding becomes less important in the triple control strategy compared with the double control strategy. It may be because case numbers decline, making the strategy more costly for every active case found.

Our principal finding in this study is that the quadruple control strategy, which includes distancing, latent case finding, case holding and active case finding control together is the most impactful and cost-effective approach for decreasing the spread of DS and MDR TB in Bangladesh. Our findings also suggests that to focus on a single control strategy will not dramatically affect the decline in DS and MDR TB in Bangladesh, whereas to combine two or more control strategies simultaneously will decrease the burden of DS and MDR TB in Bangladesh, which is found to be consistent with previous works [13, 37, 38, 47].

In Bangladesh, infectious disease surveillance does not detect all cases of tuberculosis, hence our estimates may be biased by underreporting. Therefore, more accurate data should be put in place to address concerns related to DS and MDR TB. Accurate data leads to better estimation and conclusions based on these data become more robust. Hence, policy-makers need to consider the possibility of under-reporting bias when analyzing our findings.

## Supporting information

S1 File (DOCX)
