# Supplementary material for: Modeling drug-resistant tuberculosis amplification rates and intervention strategies in Bangladesh
Source: PLoS One. 2020 Jul 23;15(7):e0236112. doi: 10.1371/journal.pone.0236112 (PMC7377424; doi:10.1371/journal.pone.0236112)
Supplement: S1 File — (DOCX) [file pone.0236112.s001.docx]

**Supporting information**

**Section S.1: Model Fit**

The fitting results of other parameter variation are shown in the table and Figs below.

**S1 Table.** Model 1 (drug-failure only): $\beta_{s},\beta_{m}=0, \tau_{s}, \tau_{m}=0 and \rho$ (3 parameter model).

| **Parameter** | **Est.** | **CI** |
| --- | --- | --- |
| $\beta_{s}$ | $1.545\times{10}^{-8}$ | $(1.481\times{10}^{-8},2.431\times{10}^{-8})$ |
| $\tau_{s}$ | $0.520$ | $(0.293, 3.653)$ |
| $\rho$ | $0.28$ | $(0.272, 0.400)$ |
| $\rho\tau_{s}$ | $0.146$ | $(0.079, 1.46)$ |
|  | **DS** | **MDR** |
| AIC | $382.54$ | $439.61$ |

**
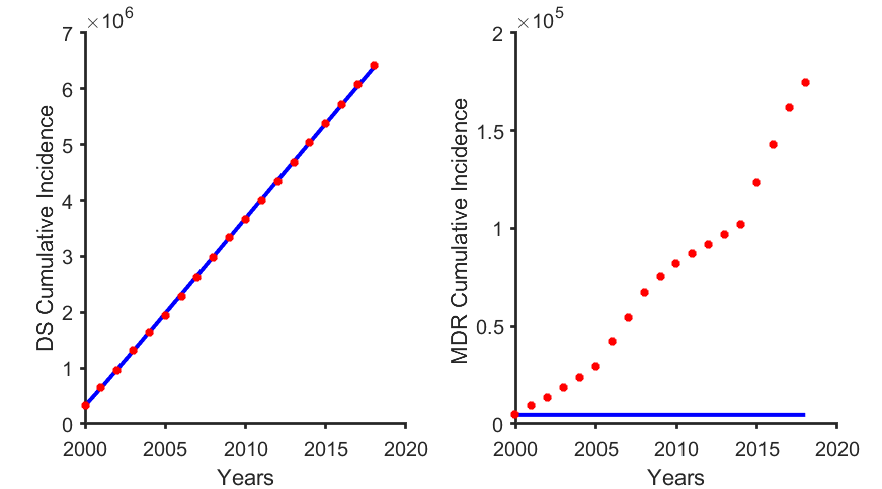
S1 Fig.** A fit of model 1 to the Bangladesh TB cumulative incidence: (A) drug- susceptible (DS) TB and (B) multi-drug resistant (MDR) TB.

**
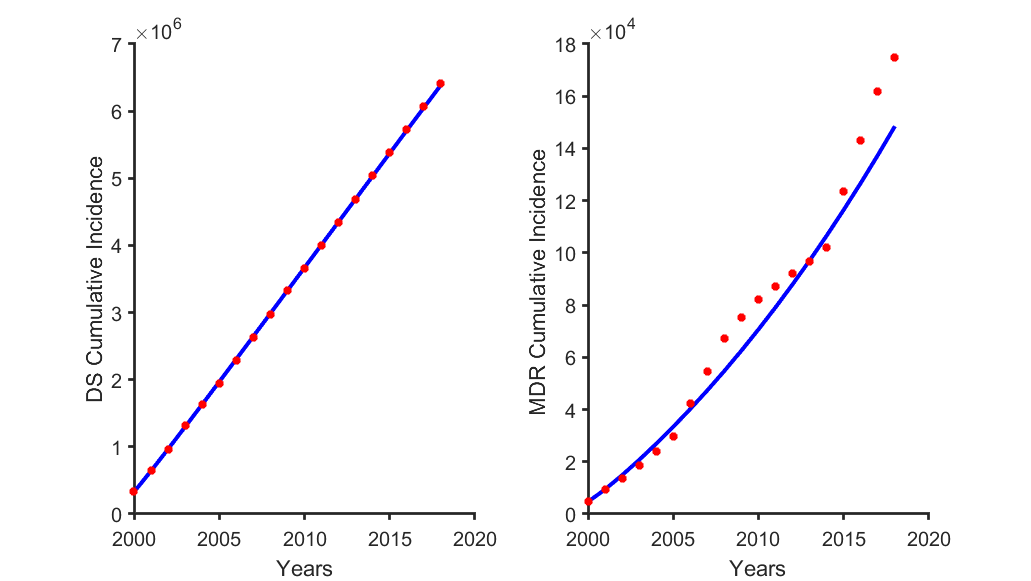
**

**S2 Fig.** A fit of model 2 to the Bangladesh TB cumulative incidence: (A) drug- susceptible (DS) TB and (B) multi-drug resistant (MDR) TB.

**S2 Table.** Model 4 (drug failure and different treatment outcome between DS and MDR): $\beta_{s}=\beta_{m}, \tau_{s}\neq\tau_{m} and \rho$ (4 parameter model).

| **Parameter** | **Est.** | **CI** |
| --- | --- | --- |
| $\beta_{s}=\beta_{m}$ | $1.55\times{10}^{-8}$ | $(1.50\times{10}^{-8}, 1.60\times{10}^{-8})$ |
| $\tau_{s}$ | $0.494$ | $(0.266, 0.724)$ |
| $\tau_{m}$ | $0.025$ | $(0, 0.301)$ |
| $\rho$ | $0.075$ | $(0.067, 0.082)$ |
| $\rho\tau_{s}$ | $0.0019$ | $(0, 0.0013)$ |
|  | **DS** | **MDR** |
| AIC | $378.79$ | $425.66$ |

**
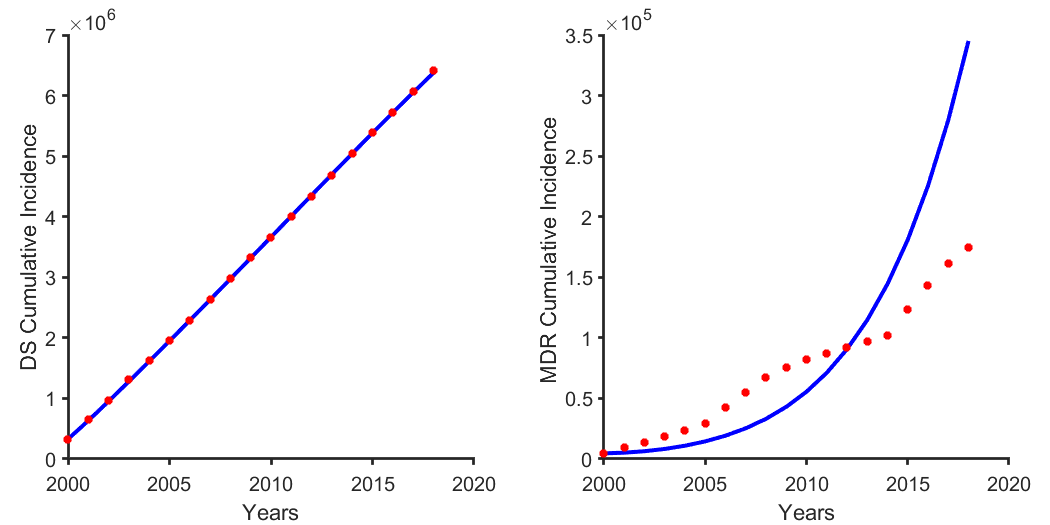
S3 Fig.** A fit of model 4 to the Bangladesh TB cumulative incidence: (A) drug- susceptible (DS) TB and (B) multi-drug resistant (MDR) TB.

**Section S.2: Optimal control analysis**

We use Pontryagin’s Maximum Principle [1] on our model (4). The aim is to reduce the number of DS and MDR TB cases and corresponding costs. Pontryagin’s Maximum Principle changes into problem that minimize pointwise a Hamiltonian H, with respect to the control. The Hamiltonian is given as

$$H=L_{s}+I_{s}+L_{m}+I_{m}+\frac{B_{1}}{2}u_{1}^{2}(t)+\frac{B_{2}}{2}u_{2}^{2}(t)+\frac{B_{3}}{2}u_{3}^{2}(t) +\frac{B_{4}}{2}u_{4}^{2}(t)$$

$+\lambda_{1}\left( \mu N-(1-u_{1}(t))\beta_{s}I_{s}S-{(1-u_{1}(t))\beta}_{m}I_{m}S-\mu S+\gamma R+\varphi_{s}I_{s}+\varphi_{m}I_{m} \right)$

$+\lambda_{2}\left( {(1-u_{1}(t))\beta}_{s}I_{s}S-\left( \alpha_{s}+{(\delta}_{s}+u_{2}\left( t \right)\tau_{1})+\mu\right)L_{s} \right)$

$+\lambda_{3}\left( \alpha_{s}L_{s}-\left( \omega_{s}+\left( 1+u_{4}\left( t \right) \right)\tau_{s}+\phi_{s}+\mu\right)I_{s} \right)$

$+\lambda_{4}\left( (1-u_{1}(t))\beta_{m}I_{m}S-\left( \alpha_{m}+{(\delta}_{m}+u_{2}\left( t \right)\tau_{2})+\mu\right)L_{m} \right)$

+$\lambda_{5}\left( \alpha_{m}L_{m}+(\rho(1-u_{3}\left( t \right)))(1+u_{4}(t))\tau_{s}I_{s}-\left( \omega_{m}+\tau_{m}\left( 1+u_{4}\left( t \right) \right)+\phi_{m}+\mu\right)I_{m} \right)$

${+\lambda}_{6}\left( \left( 1-\rho\left( 1-u_{3}\left( t \right) \right) \right)(1+u_{4}(t))\tau_{s}I_{s}+\tau_{m}\left( 1+u_{4}\left( t \right) \right)I_{m}+\omega_{s}I_{s}+\omega_{m}I_{m}+{(\delta}_{s}+u_{2}\left( t \right)\tau_{1})L_{s} +\left( \delta_{m}+u_{2}\left( t \right)\tau_{2} \right)L_{m}-\gamma R-\mu R \right)$.

Now using Pontryagin’s maximum principle, we obtain the following theorem.

**Theorem:** There exist optimal controls$u_{1}^{*}\left( t \right), u_{2}^{*}(t)$,$u_{3}^{*}(t)$, and $u_{4}^{*}(t)$ minimizing the objective function $\Omega=\left\{ \left( u_{1}, u_{2}, u_{3},u_{4} \right)| a\leq u_{i}\left( t \right)\leq b, u_{i}\epsilon\mathcal{L}^{2}\left( 0, 20 \right), i=1,2,3,4 \right\}$.

Given these optimal solutions, there exist adjoint variables,$\lambda_{1}, \lambda_{2}, \lambda_{3}, \lambda_{4}, \lambda_{5}$, and $\lambda_{6}$ which satisfy,

$\frac{d\lambda_{1}}{\mathrm{dt}}=\lambda_{1}\left( \left( 1-u_{1} \right)\beta_{s}I_{s}+\left( 1-u_{1} \right)\beta_{m}I_{m}+\mu\right)-\lambda_{2}\left( 1-u_{1} \right)\beta_{s}I_{s}-\lambda_{4}\left( 1-u_{1} \right)\beta_{m}I_{m}$,

$\frac{d\lambda_{2}}{\mathrm{dt}}=-1+\lambda_{2}\left\{ \alpha_{s}+\left( \delta_{s}+u_{2}\tau_{1} \right)+\mu\right\}-\lambda_{3}\alpha_{s}-\lambda_{6} (\delta_{s}+u_{2}\tau_{1})$,

$$\frac{d\lambda_{3}}{\mathrm{dt}}=-1+\lambda_{1}\left( \left( 1-u_{1} \right)\beta_{s}S-\phi_{s} \right)-\lambda_{2}\left( 1-u_{1} \right)\beta_{s}S+\lambda_{3}\left( \omega_{s}+\phi_{s}+\tau_{s}\left( 1+u_{4} \right)+\mu\right)$$

$-\lambda_{5}\rho\left( 1-u_{3} \right){(1+u_{4})\tau}_{s}-\lambda_{6}\left( \left( 1-\rho\left( 1-u_{3} \right) \right){(1+u_{4})\tau}_{s}+\omega_{s} \right)$,

$\frac{d\lambda_{4}}{\mathrm{dt}}=-1+\lambda_{4}\left\{ \alpha_{m}+\left( \delta_{m}+u_{2}\tau_{2} \right)+\mu\right\}-\lambda_{5}\alpha_{m}-\lambda_{6}\left( \delta_{m}+u_{2}\tau_{2} \right)$,

$$\frac{d\lambda_{5}}{\mathrm{dt}}=-1+\lambda_{1}\left\{ \left( 1-u_{1} \right)\beta_{m}S-\phi_{m} \right\}-\lambda_{4}\left( 1-u_{1} \right)\beta_{m}S+\lambda_{5}\left( \omega_{m}+\phi_{m}+\tau_{m}\left( 1+u_{4} \right)+\mu\right)$$

$-\lambda_{6}(\omega_{m}+\tau_{m}(1+u_{4}))$,

$\frac{d\lambda_{6}}{\mathrm{dt}}=\lambda_{6}\left( \gamma+\mu\right)-\lambda_{1}\gamma$.

with transversality conditions $\lambda_{i}=0$, for $i=1, 2, 3, 4, 5, 6$.

Furthermore,

$u_{1}^{*}\left( t \right)=min\left( b, max\left( a, \frac{1}{B_{1}}\left( \left( \lambda_{2}-\lambda_{1} \right)\beta_{s}I_{s}S+\left( \lambda_{4}-\lambda_{1} \right)\beta_{m}I_{m}S \right) \right) \right)$,

$u_{2}^{*}\left( t \right)=min\left( b, max\left( a, \frac{1}{B_{2}}\left( \left( \lambda_{2}-\lambda_{6} \right)\tau_{1}L_{s}+\left( \lambda_{4}-\lambda_{6} \right)\tau_{2}L_{m} \right) \right) \right)$,

$u_{3}^{*}\left( t \right)=min\left( b, max\left( a, m_{1}+\frac{m_{1}}{m_{2}}\left( m_{3}+\left( \lambda_{5}-\lambda_{6} \right)\tau_{m}I_{m}+\left( \lambda_{5}-\lambda_{6} \right)m_{1}\rho\tau_{s}I_{s} \right) \right) \right)$,

$u_{4}^{*}\left( t \right)=min\left( b, max\left( a, \frac{1}{m_{2}}\left( m_{3}+\left( \lambda_{5}-\lambda_{6} \right)\tau_{m}I_{m}+\left( \lambda_{5}-\lambda_{6} \right)m_{1}\rho\tau_{s}I_{s} \right) \right) \right)$,

Where,

$m_{1}=\frac{\left( \lambda_{5}-\lambda_{6} \right)\rho\tau_{s}I_{s}}{B_{3}}$ , $m_{2}=B4-\left( \lambda_{5}-\lambda_{6} \right)\rho\tau_{s}I_{s}m_{1}$, and $m_{3}=\left( \lambda_{3}-\rho\lambda_{5}-\lambda_{6}+\rho\lambda_{6} \right)\tau_{s}I_{s}$.

**Proof:** The existence of optimal controls$u_{1}^{*}\left( t \right), u_{2}^{*}(t)$, $u_{3}^{*}(t)$ and $u_{4}^{*}(t)$ such that

$J\left( u_{1}^{*}\left( t \right), u_{2}^{*}\left( t \right), u_{3}^{*}\left( t \right), u_{4}^{*}(t) \right)=={}_{\Omega}^{\min}{J(u_{1}, u_{2}, u_{3}, u_{4})}$ with state system (4) is given by the convexity of the objective functional integrand. By Pontryagin’s Maximum Principle [1], the adjoint equations and transversality conditions are obtained. Differentiation of Hamiltonian H with respect to the state variables gives the following system,

$\frac{d\lambda_{1}}{\mathrm{dt}}=-\frac{\partial H}{\partial S}$,

$\frac{d\lambda_{2}}{\mathrm{dt}}=-\frac{\partial H}{\partial L_{s}}$,

$\frac{d\lambda_{3}}{\mathrm{dt}}=-\frac{\partial H}{\partial I_{s}}$,

$\frac{d\lambda_{4}}{\mathrm{dt}}=-\frac{\partial H}{\partial L_{m}}$,

$\frac{d\lambda_{5}}{\mathrm{dt}}=-\frac{\partial H}{\partial I_{m}}$,

$\frac{d\lambda_{6}}{\mathrm{dt}}=-\frac{\partial H}{\partial R}$,

with $\lambda_{i}=0$, for $i=1, 2, 3, 4, 5, 6$.

Optimal controls$u_{1}^{*}\left( t \right), u_{2}^{*}(t)$, $u_{3}^{*}(t)$ and $u_{4}^{*}\left( t \right)$are derived by the following optimality conditions

$\frac{\partial H}{\partial u_{1}}=B_{1}u_{1}+\lambda_{1}\beta_{s}I_{s}S+\lambda_{1}\beta_{m}I_{m}S-\lambda_{2}\beta_{s}I_{s}S-\lambda_{4}\beta_{m}I_{m}S=0$,

$\frac{\partial H}{\partial u_{2}}=B_{2}u_{2}-\lambda_{2}\tau_{1}L_{s}-\lambda_{4}\tau_{2}L_{m}+\lambda_{6}{\tau_{1}L}_{s}+\lambda_{6}\tau_{2}L_{m}=0$,

$\frac{\partial H}{\partial u_{3}}=B_{3}u_{3}-\lambda_{5} \rho(1+u_{4})\tau_{s}I_{s}+\lambda_{6}\rho(1+u_{4})\tau_{s}I_{s}=0$,

$\frac{\partial H}{\partial u_{4}}=B_{4}u_{4}-\lambda_{3}\tau_{s}I_{s}+\lambda_{5}\rho\left( 1-u_{3} \right)\tau_{s}I_{s}-\lambda_{5}\tau_{m}I_{m}+\lambda_{6}\left( 1-\rho\left( 1-u_{3} \right) \right){\left( 1+u_{4} \right)\tau}_{s}I_{s}+\lambda_{6}\tau_{m}I_{m}=0$,

at$u_{1}^{*}\left( t \right), u_{2}^{*}(t)$, $u_{3}^{*}(t)$ and $u_{4}^{*}\left( t \right)$ on the set $\Omega$. On this set

$u_{1}^{*}(t)=\frac{\left( \lambda_{2}-\lambda_{1} \right)\beta_{s}I_{s}S+\left( \lambda_{4}-\lambda_{1} \right)\beta_{m}I_{m}S}{B_{1}}$,

$u_{2}^{*}\left( t \right)=\frac{\left( \lambda_{2}-\lambda_{6} \right)\tau_{1}L_{s}+\left( \lambda_{4}-\lambda_{6} \right)\tau_{2}L_{m}}{B_{2}}$,

$u_{3}^{*}\left( t \right)=\frac{\left( \lambda_{5}-\lambda_{6} \right) \rho{(1+u_{4})\tau}_{s}I_{s}}{B_{3}}$,

$u_{4}^{*}\left( t \right)=\frac{\left( \lambda_{3}-\rho\left( 1-u_{3} \right)\lambda_{5}-\left( 1-\rho(1-u_{3}) \right)\lambda_{6} \right)\tau_{s}I_{s}+\left( \lambda_{5}-\lambda_{6} \right)\tau_{m}I_{m}}{B_{4}}$.

The Figs below shows the optimal solutions of our control strategies.

| (A)  **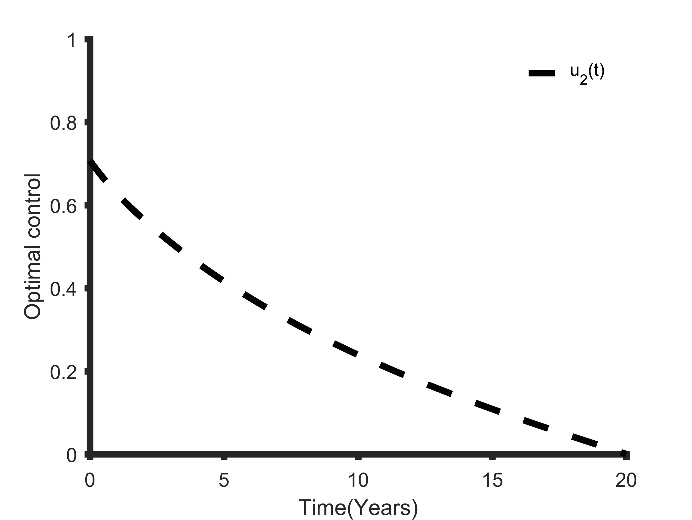** | (B)  **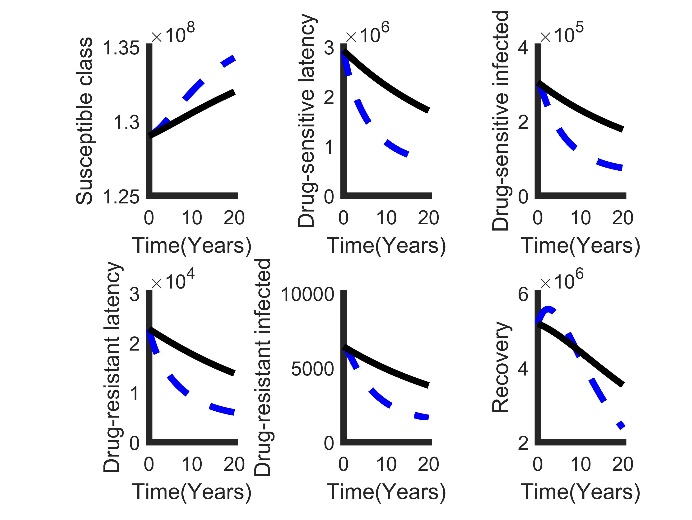** |
| --- | --- |
| (C)  **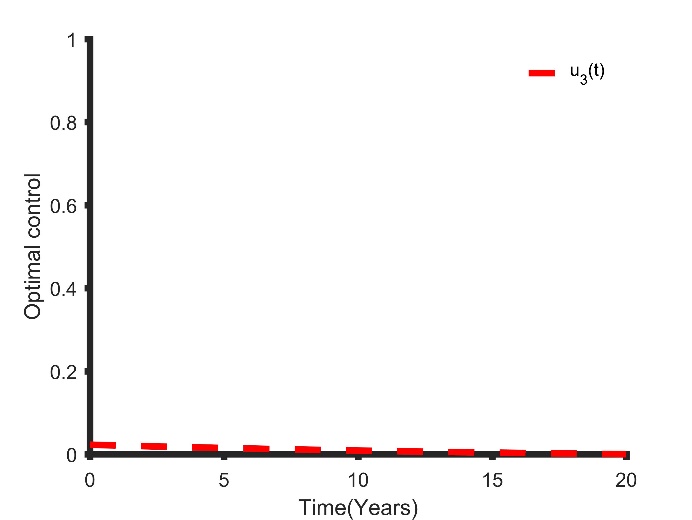** | (D)  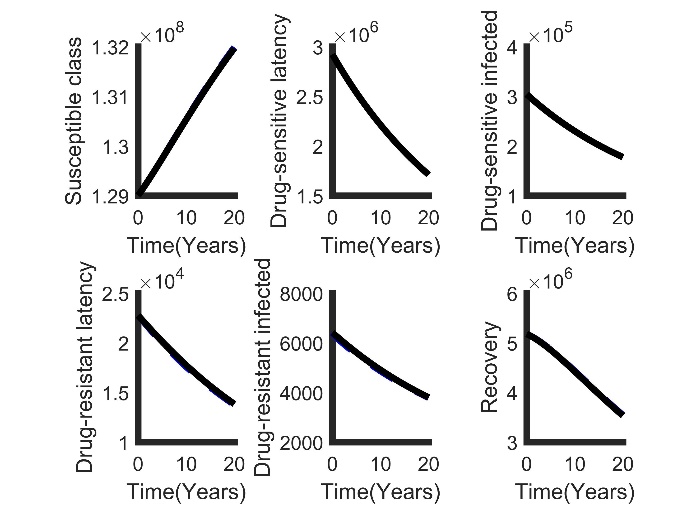 |
| (E)  **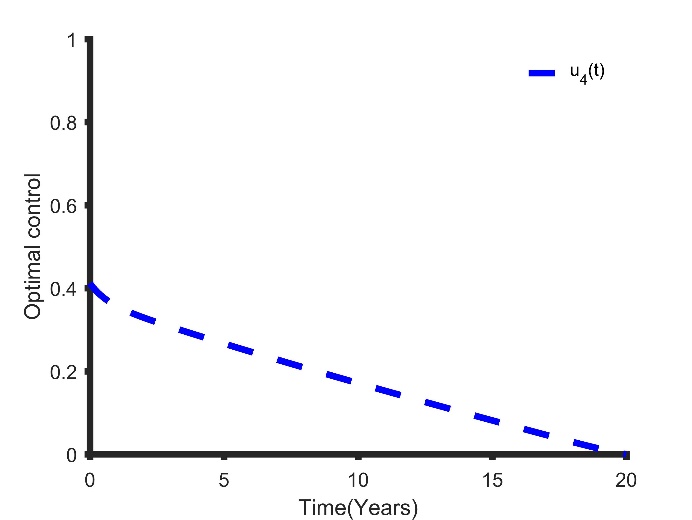** | (F)  **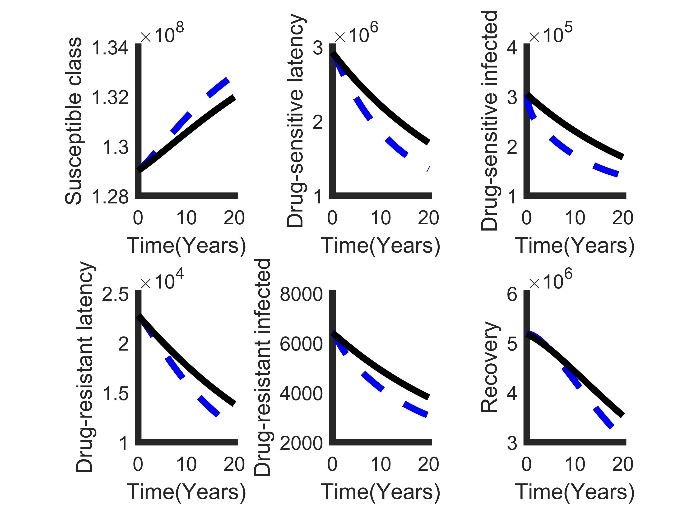** |

**S4 Fig.** The single optimal control strategy: (A) The optimal latent case finding control. (B) The corresponding benefits of the latent case finding. (C) The optimal case holding strategy. (D) The corresponding effects of using the case holding strategy. (E) The optimal active case finding strategy. (F) The benefits of optimal active case finding.

| (A)  **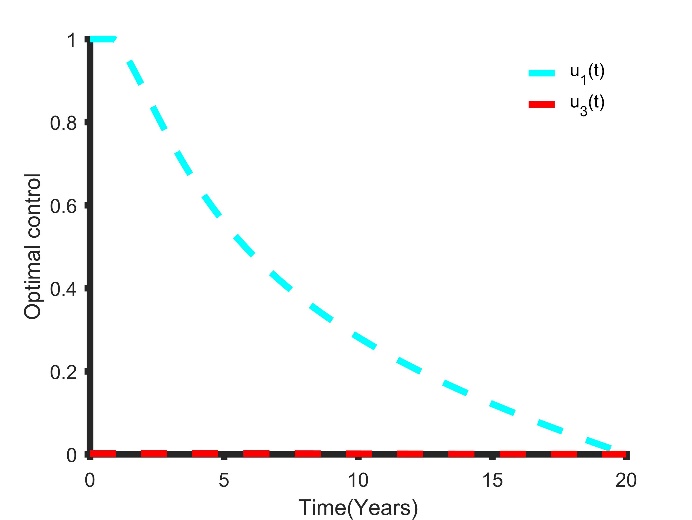** | (B)  **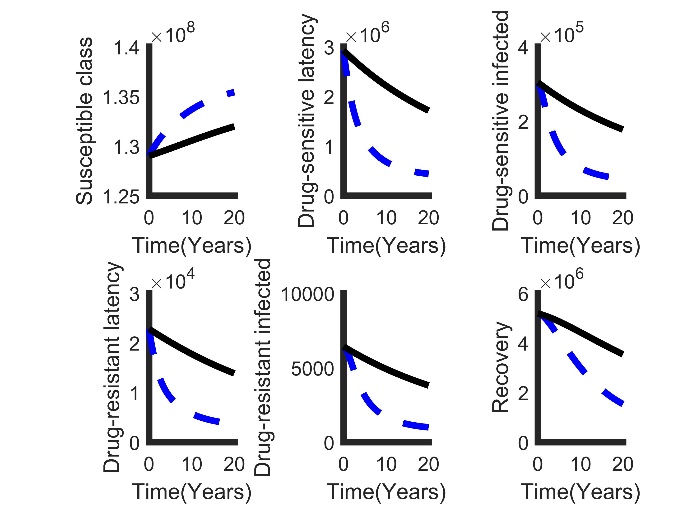** |
| --- | --- |
| (C)  **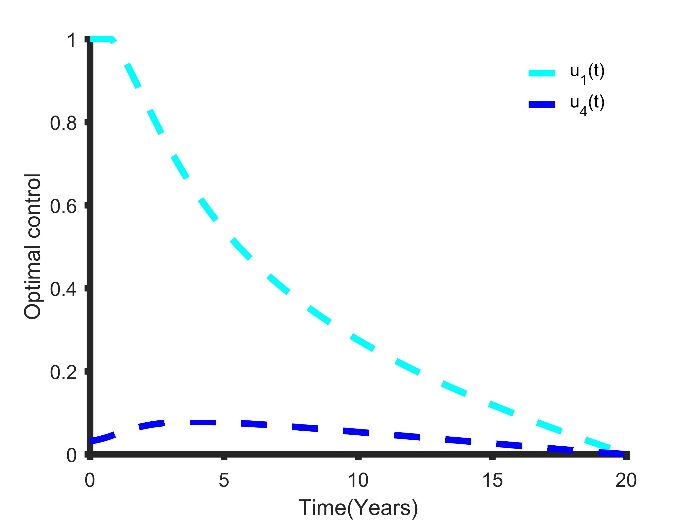** | (D)  **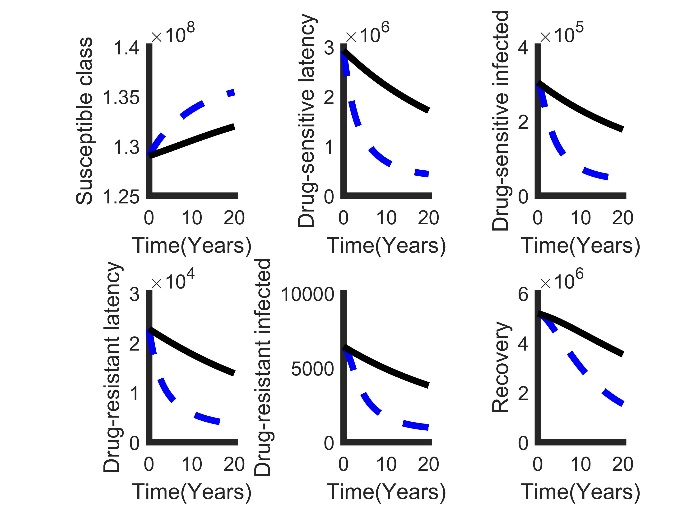** |
| (E)  **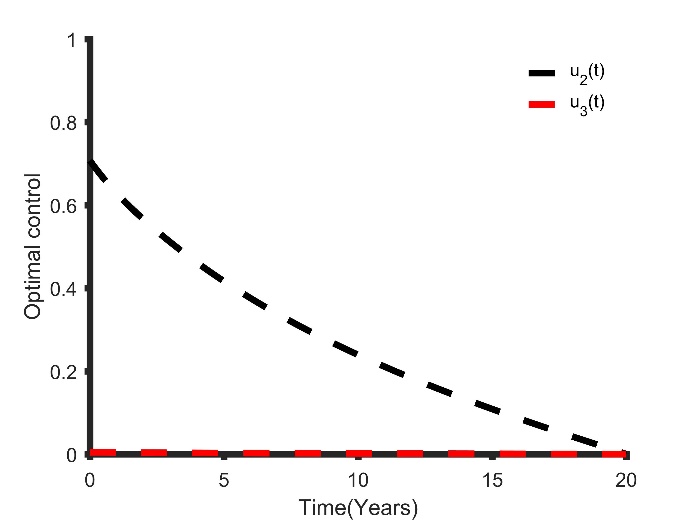** | (F)  **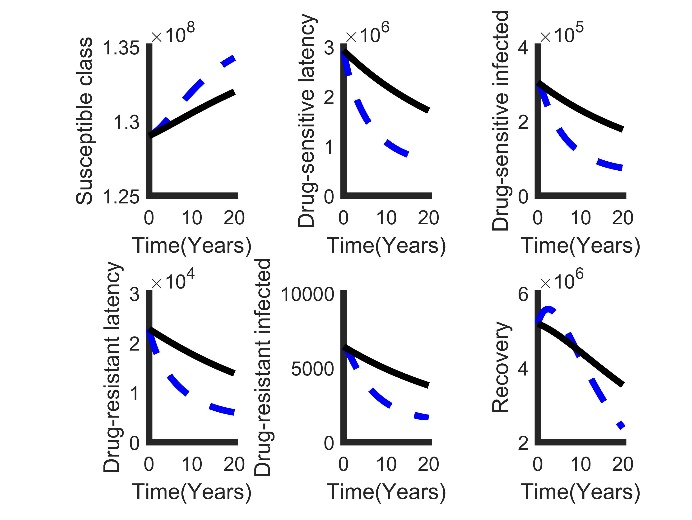** |
| (G)  **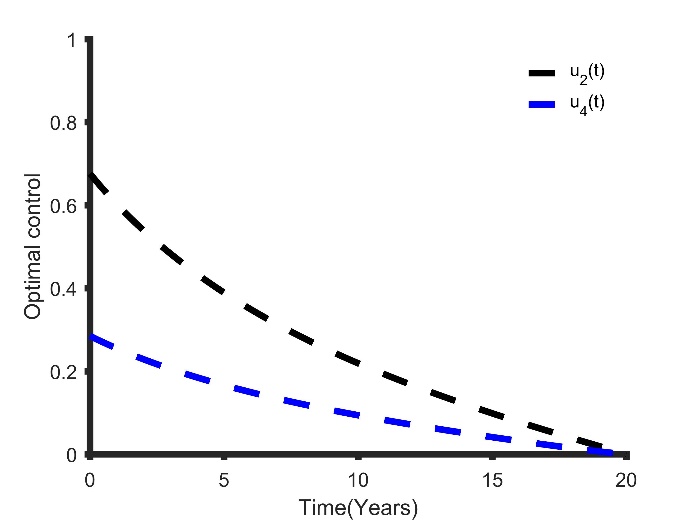** | (H)  **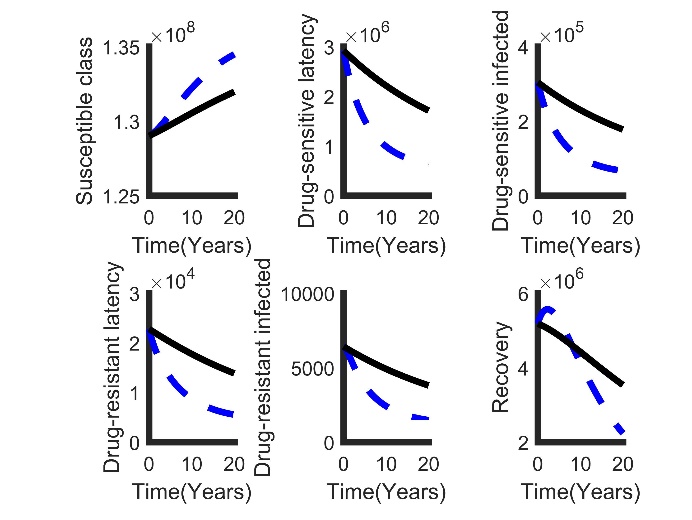** |
| (I)  **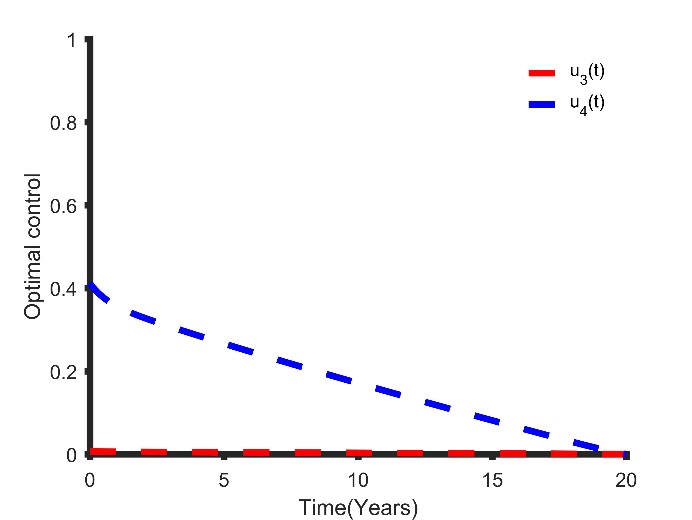** | (J)  **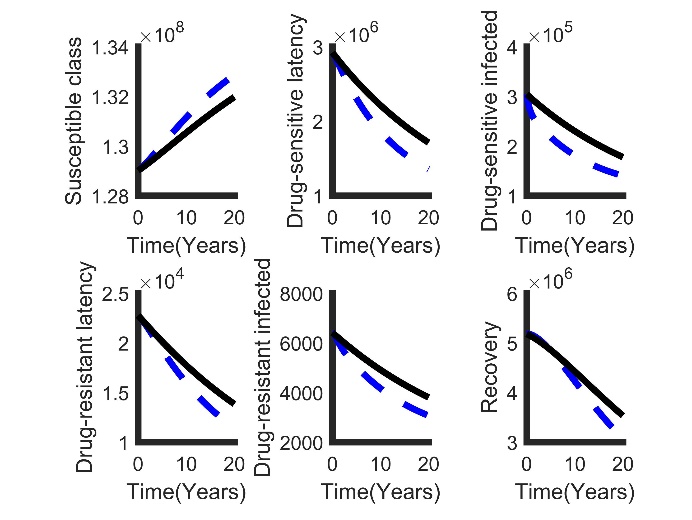** |

**S5 Fig.** The double optimal control strategy: (A) The optimal distancing and case holding strategies. (B) The corresponding benefits of the distancing and case holding. (C) The optimal distancing and active case finding strategies. (D) The corresponding effects of using the distancing and active case finding. (E) The optimal latent case finding and case holding strategies. (F) The benefits of optimal latent case finding and case holding. (G) The optimal latent and active case finding strategies. (H) The benefits of optimal latent and active case finding. (I) The optimal case holding and active case finding strategies. (J) The benefits of optimal case holding and active case finding.

| (A)  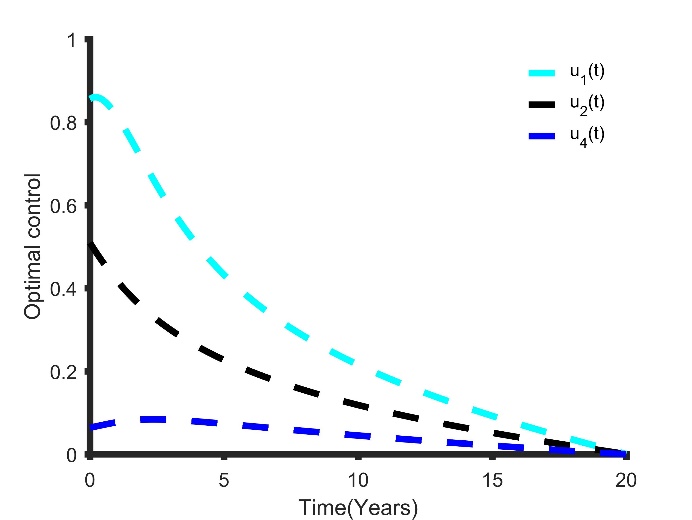 | (B)  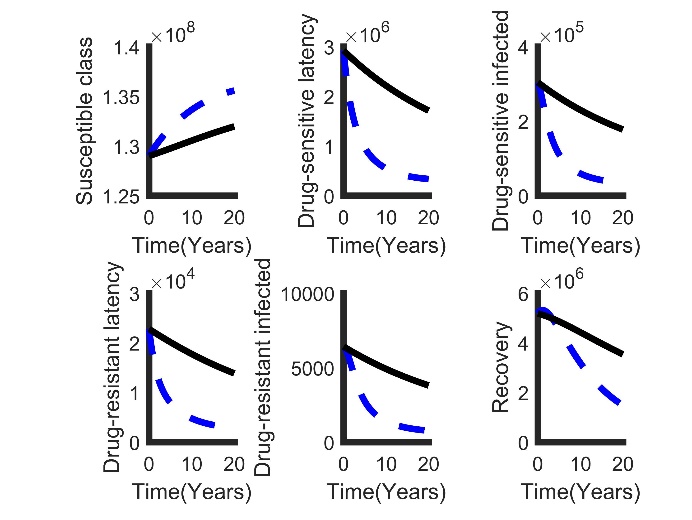 |
| --- | --- |
| (C)  **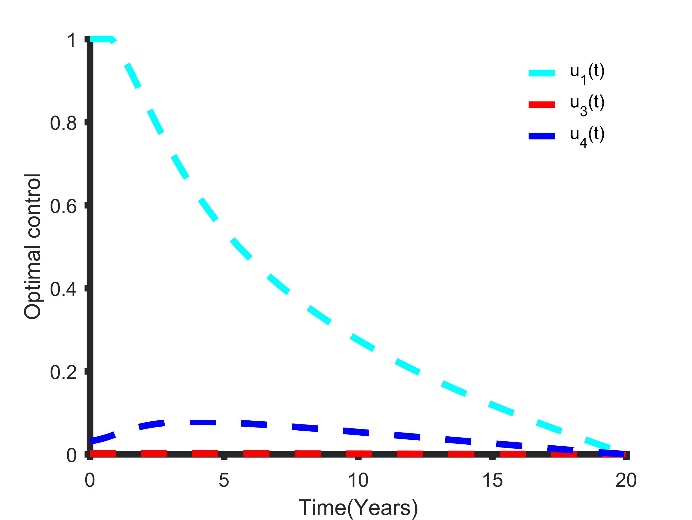** | (D)  **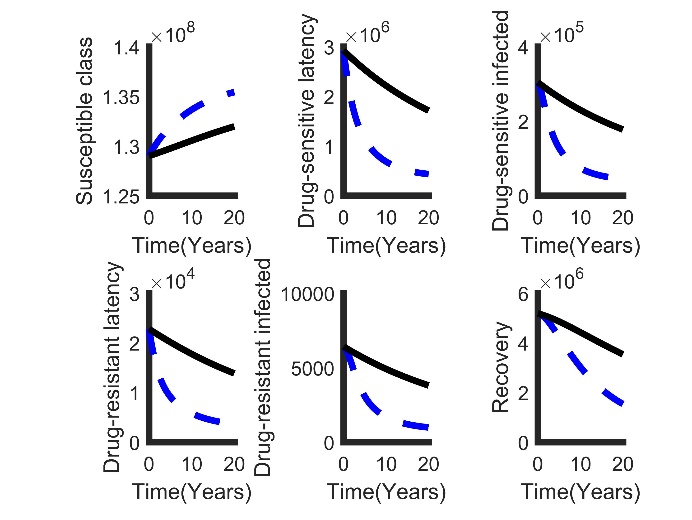** |
| (E)  **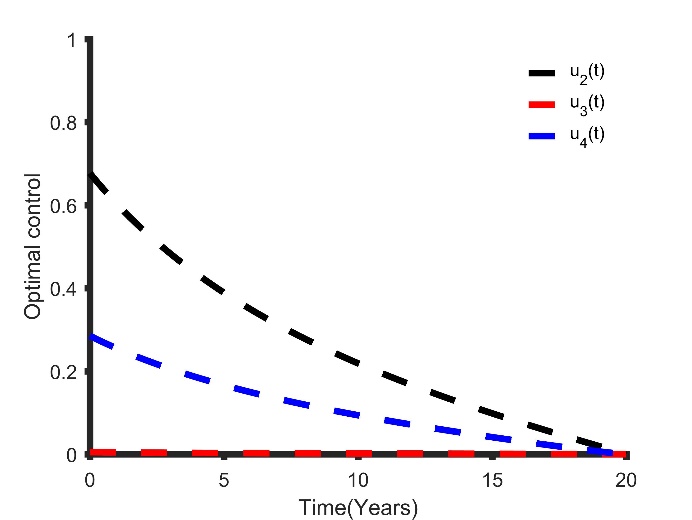** | (F)  **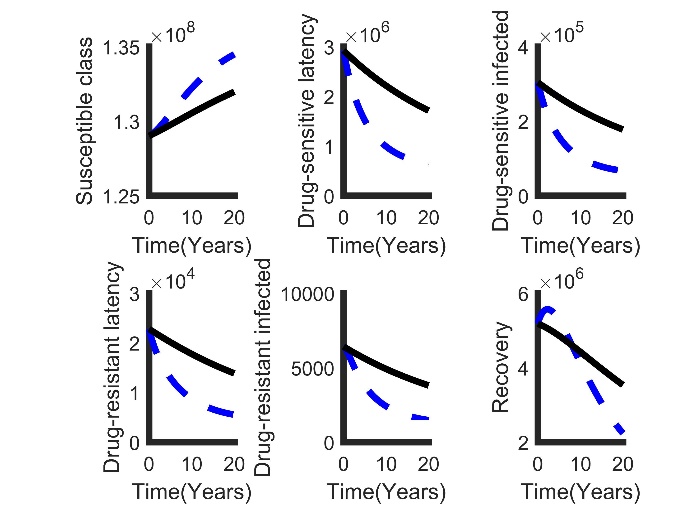** |

**S6 Fig.** The tripled optimal control strategy: (A) The optimal distancing, latent and active case finding control strategy. (B) The corresponding benefits of the distancing, latent case finding and case holding. (C) The optimal distancing, case holding and active case finding strategy. (D) The corresponding effects of using the distancing, case holding and active case finding. (E) The optimal latent case finding, case holding and active case finding strategy. (F) The benefits of optimal latent case finding, case holding and active case finding.

**Section S. 3**

**
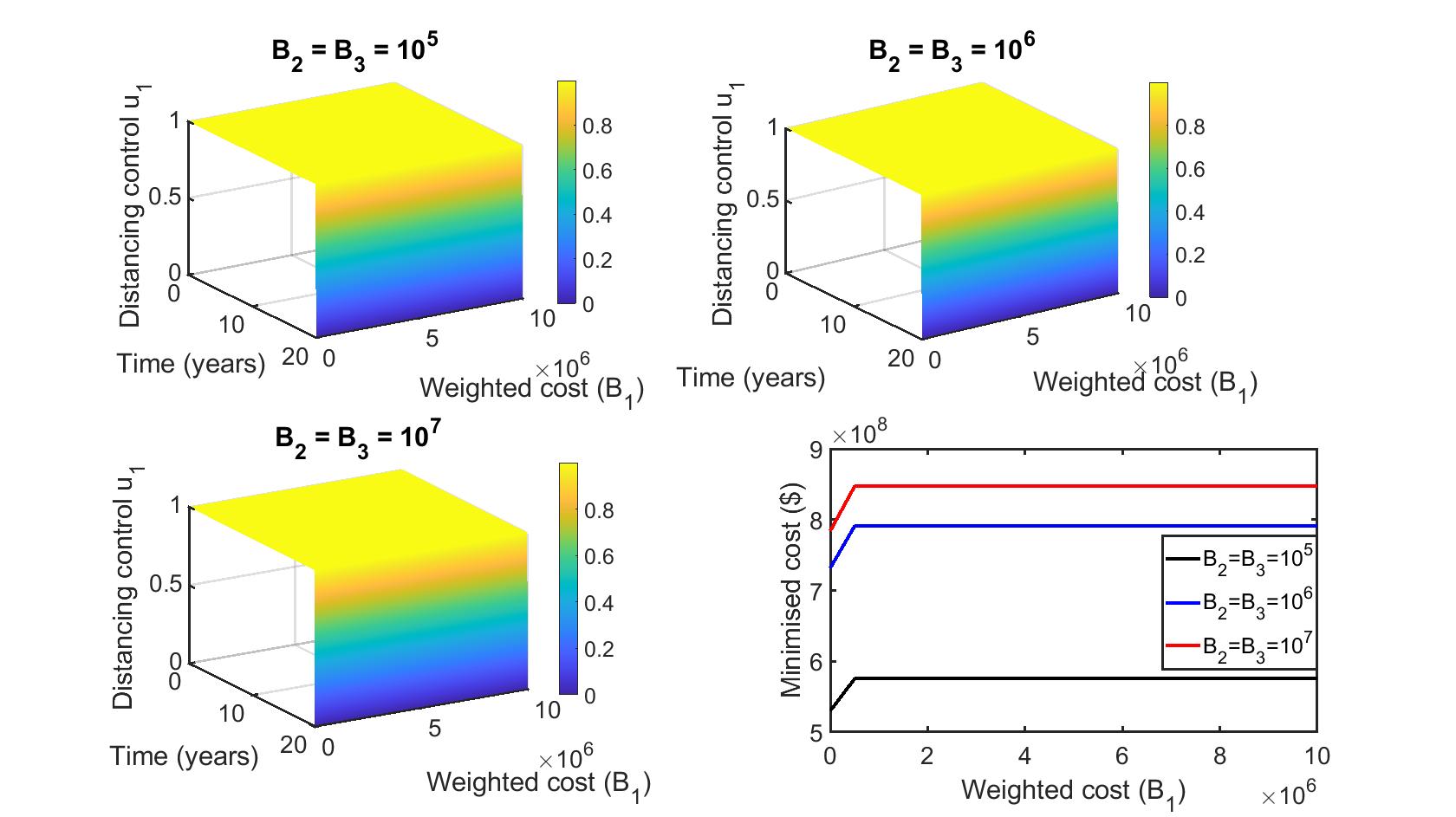
**

**S7 Fig (A).** Combination of distancing$\left( u_{1} \right)$, latent case finding$(u_{2})$ and case holding $(u_{3})$strategy, and considering distancing control $\left( u_{1} \right)$ strategy as a function of time and weighted cost$(B_{1})$. The weighted cost ${(B}_{2}\mathrm{and}B_{3})$ determined by three threshold values$B_{2}{=B}_{3}=10^{5}=10^{6}=10^{7}$.

**
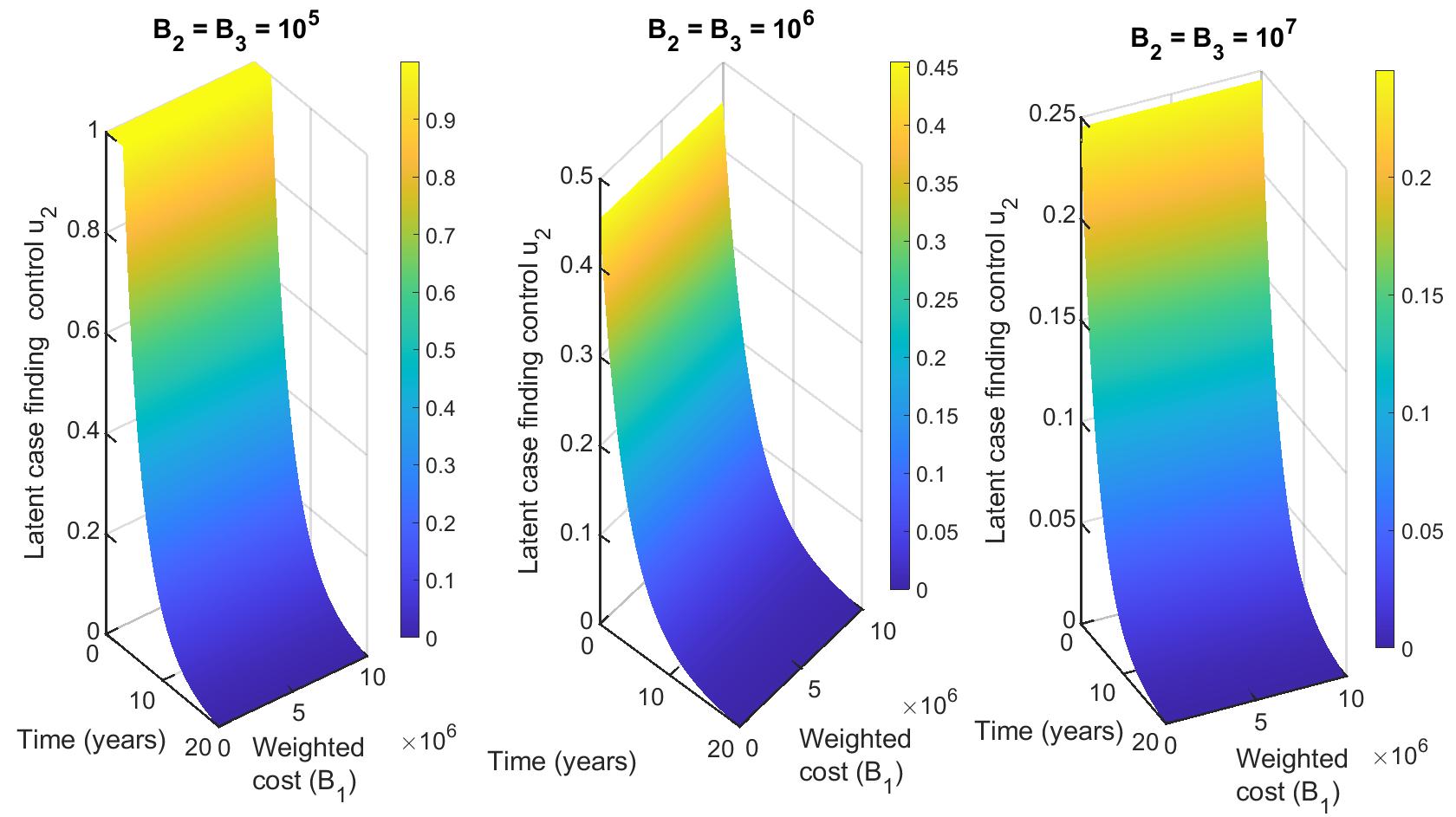
**

**S7 Fig (B).** Combination of distancing$\left( u_{1} \right)$, latent case finding$(u_{2})$ and case holding $(u_{3})$strategy, and considering latent case finding control $\left( u_{2} \right)$ strategy as a function of time and weighted cost$(B_{1})$. The weighted cost ${(B}_{2}\mathrm{and}B_{3})$ determined by three threshold values$B_{2}{=B}_{3}=10^{5}=10^{6}=10^{7}$.

**
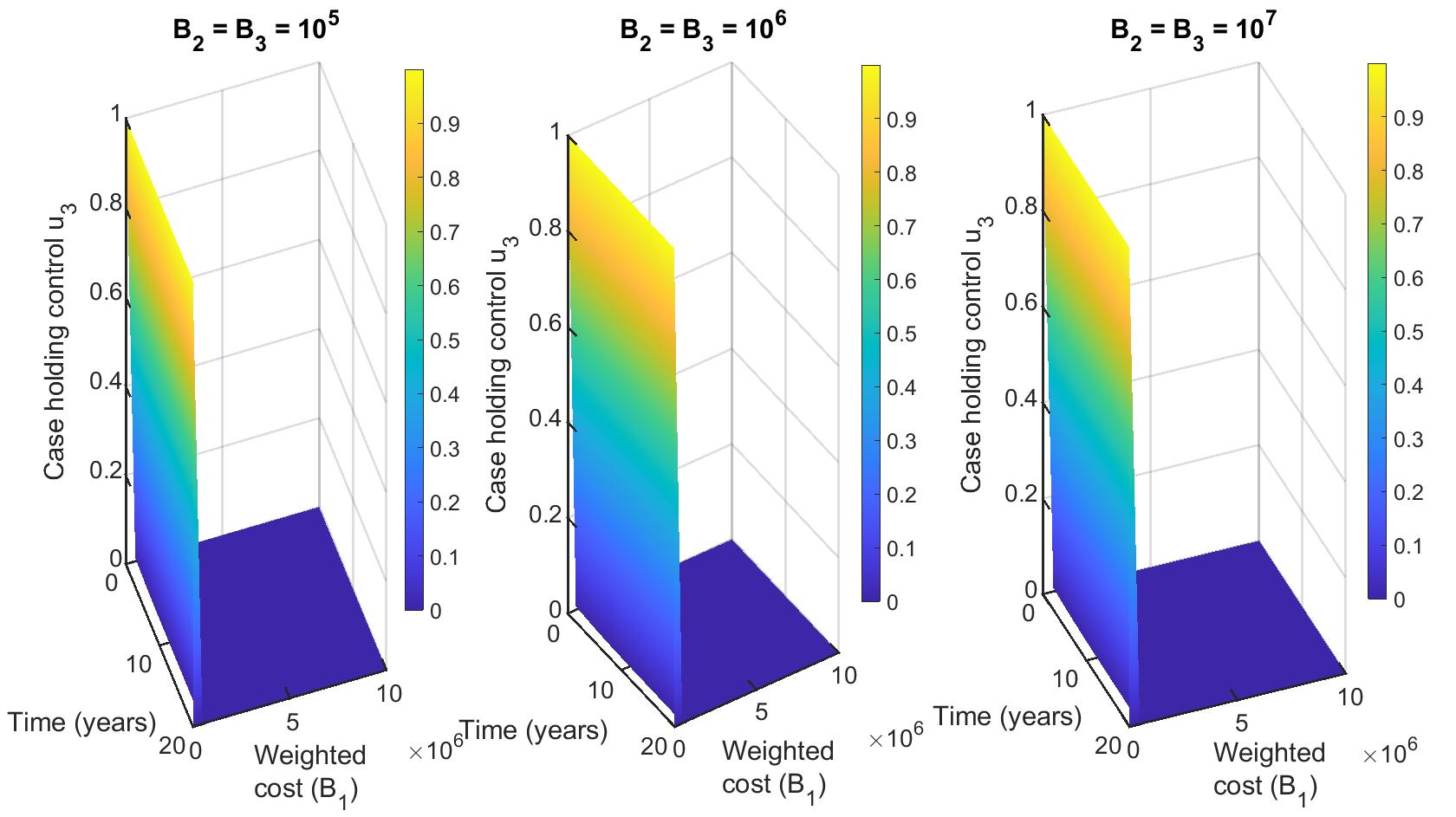
**

**S7** **Fig (C).** Combination of distancing$\left( u_{1} \right)$, latent case finding$(u_{2})$ and case holding $(u_{3})$strategy, and considering case holding control $\left( u_{2} \right)$ strategy as a function of time and weighted cost$(B_{1})$. The weighted cost ${(B}_{2}\mathrm{and}B_{3})$ determined by three threshold values$B_{2}{=B}_{3}=10^{5}=10^{6}=10^{7}$.

**
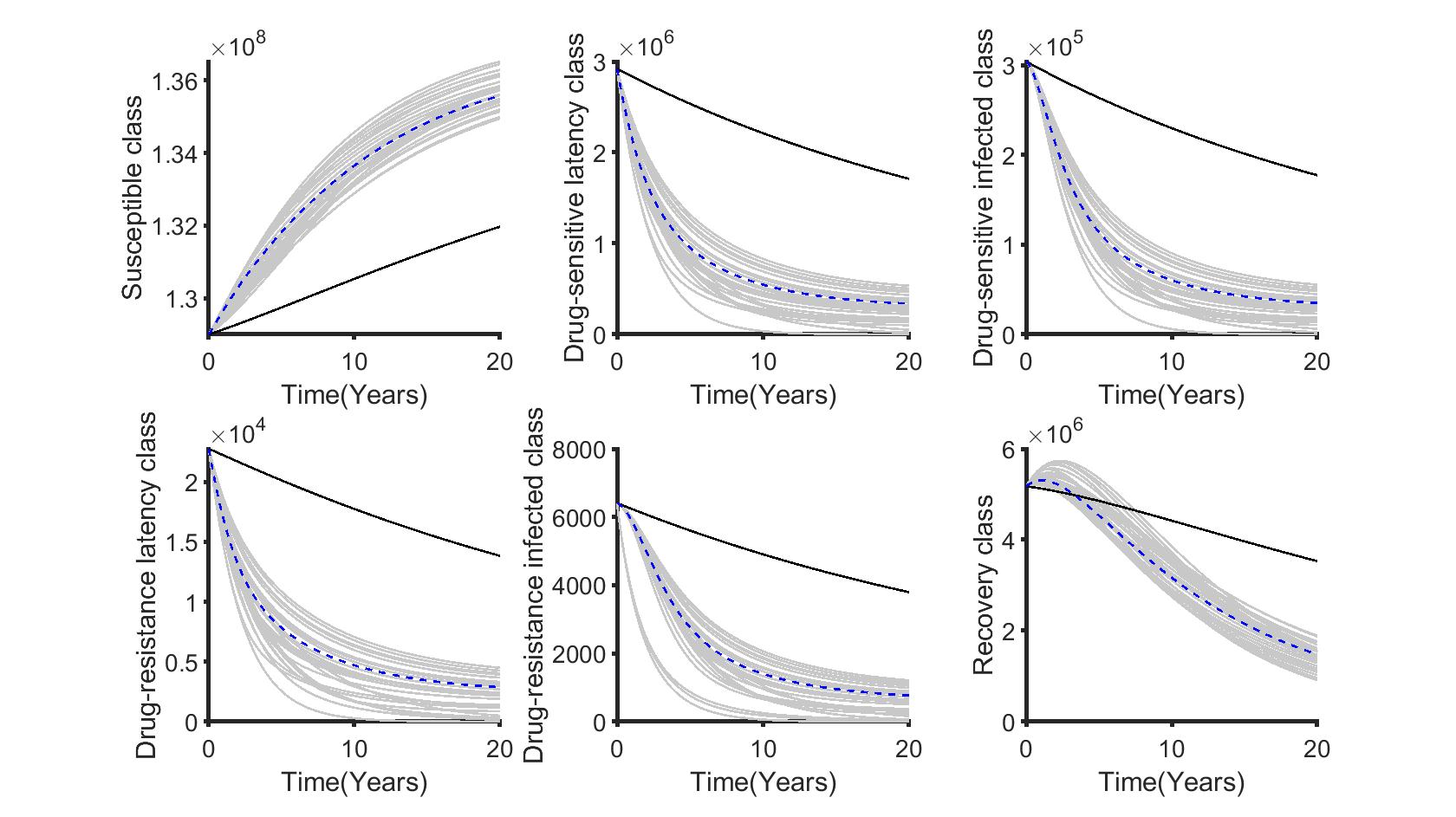
**

**S7 Fig (D).** The corresponding state variables of the combination of distancing control$(u_{1})$, latent case finding $(u_{2})$ and case holding $(u_{3})$ control strategy and considering the weighted cost $B_{1}$ is varied and $B_{2}{=B}_{3}=10^{5}=10^{6}=10^{7}$. The state variables with and without controls are plotted by grays and black lines respectively.

**
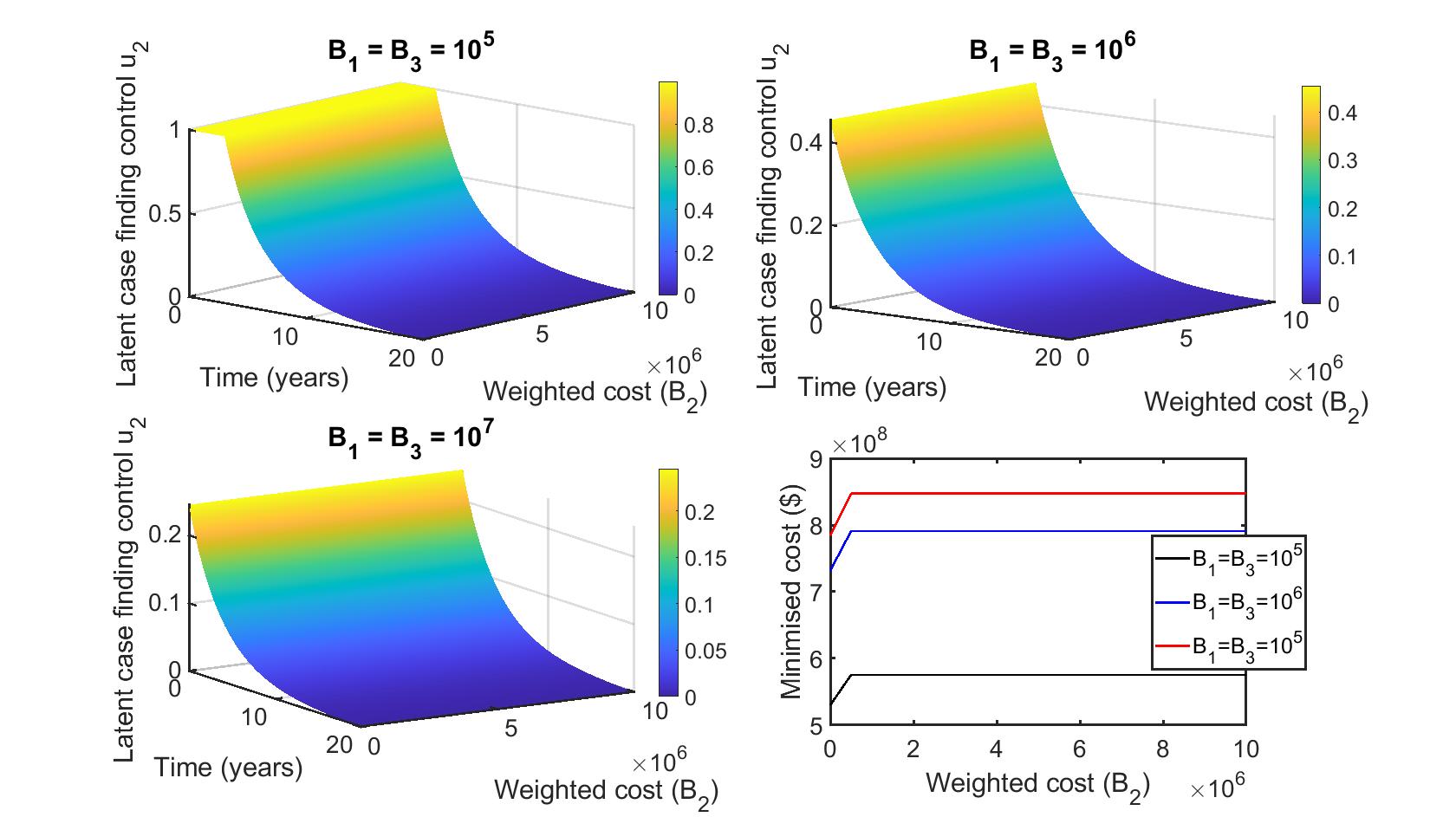
**

**S8 Fig (A).** Combination of distancing$\left( u_{1} \right)$, latent case finding$(u_{2})$ and case holding $(u_{3})$strategy, and considering latent case finding control $\left( u_{2} \right)$ strategy as a function of time and weighted cost$(B_{2})$. The weighted cost ${(B}_{1}\mathrm{and}B_{3})$ determined by three threshold values$B_{1}{=B}_{3}=10^{5}=10^{6}=10^{7}$.


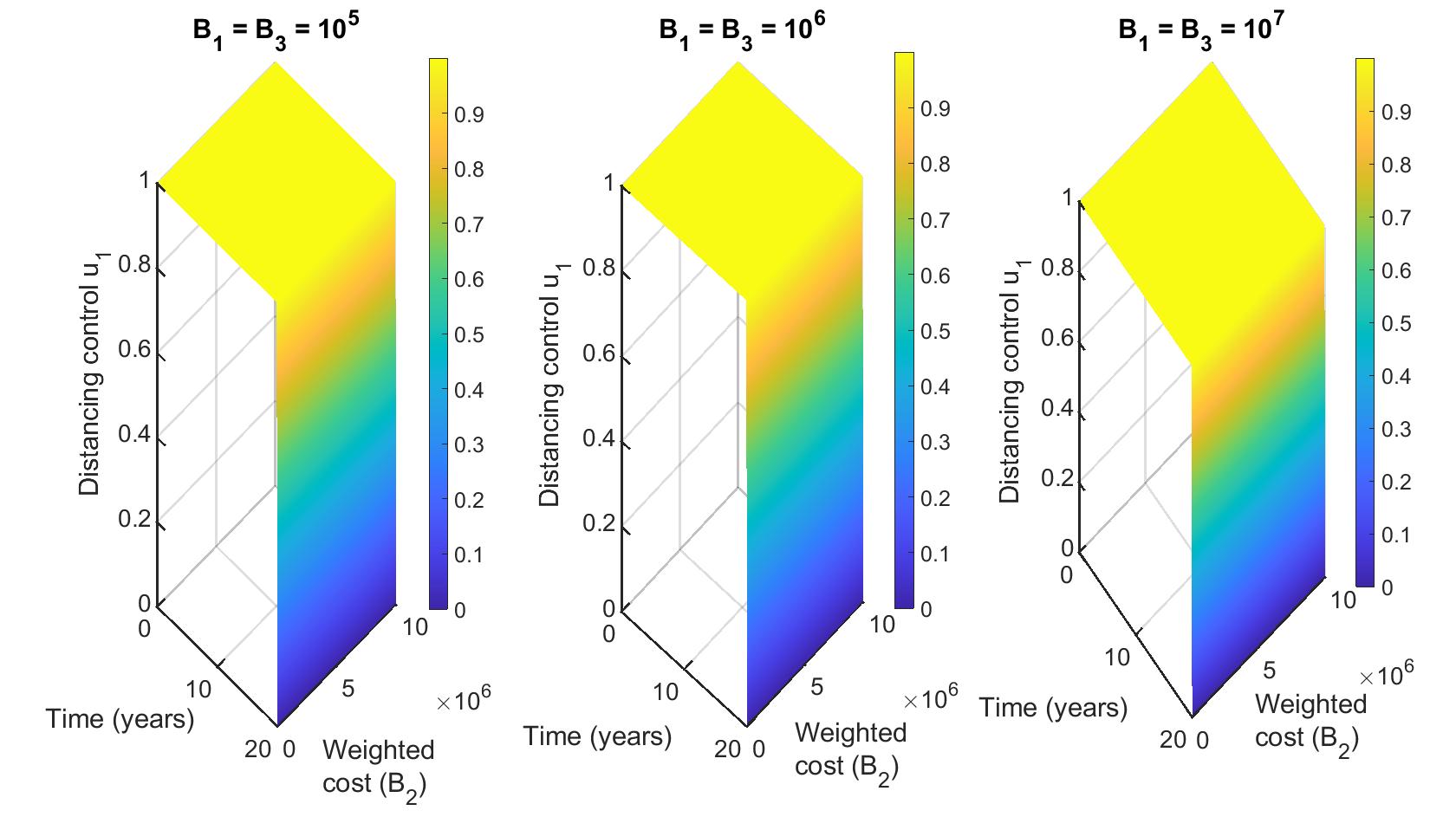


**S8 Fig (B).** Effects of combining of distancing$\left( u_{1} \right)$, latent case finding$(u_{2})$ and case holding $(u_{3})$strategy, and considering distancing control $\left( u_{1} \right)$ strategy as a function of time and weighted cost$(B_{2})$. The weighted cost ${(B}_{1}\mathrm{and}B_{3})$ determined by three threshold values$B_{1}{=B}_{3}=10^{5}=10^{6}=10^{7}$.

**
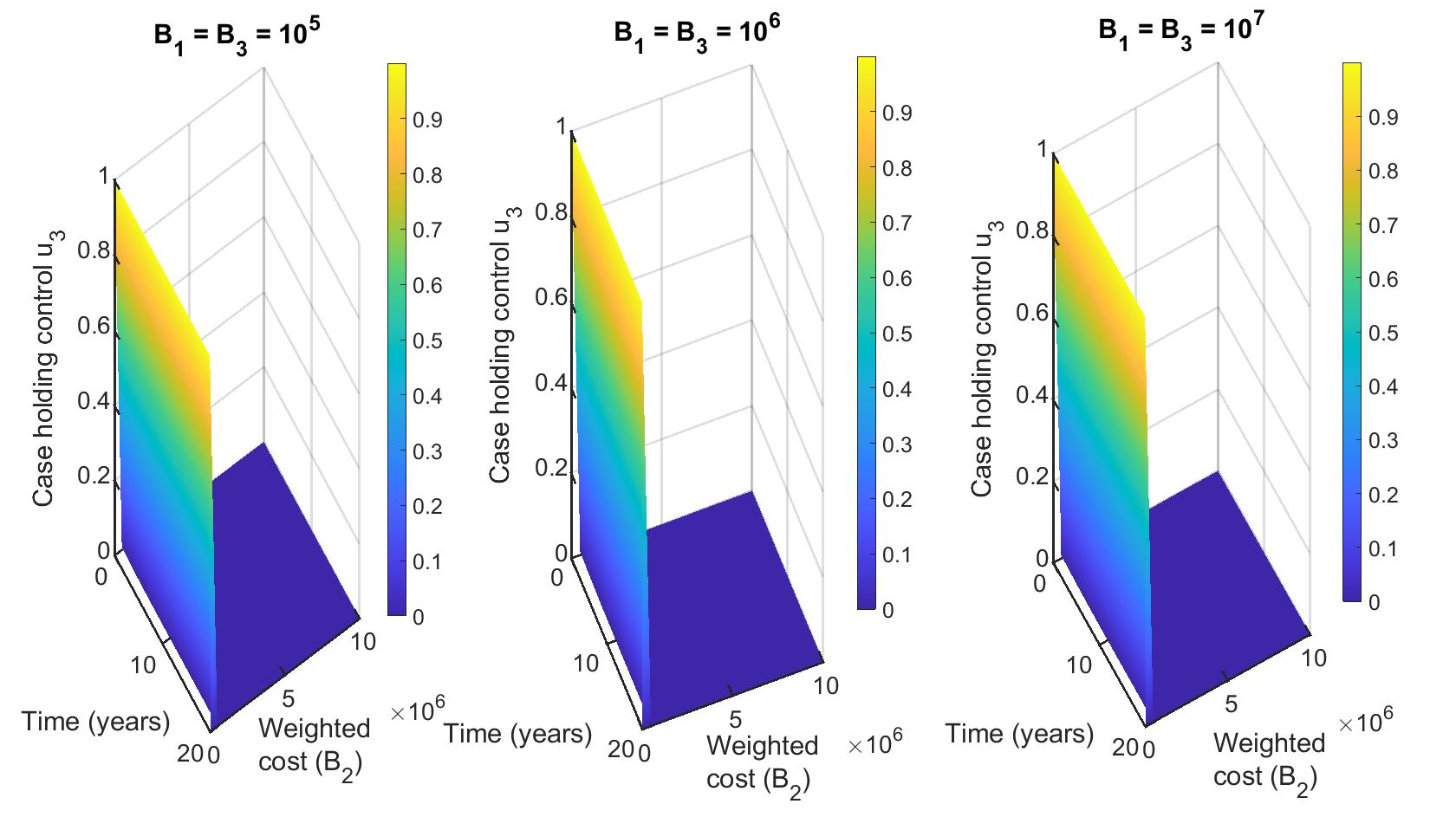
**

**S8 Fig (C**). Combination of distancing$\left( u_{1} \right)$, latent case finding$(u_{2})$ and case holding $(u_{3})$strategy, and considering case holding control $\left( u_{3} \right)$ strategy as a function of time and weighted cost$(B_{2})$. The weighted cost ${(B}_{1}\mathrm{and}B_{3})$ determined by three threshold values$B_{1}{=B}_{3}=10^{5}=10^{6}=10^{7}$**.**

**
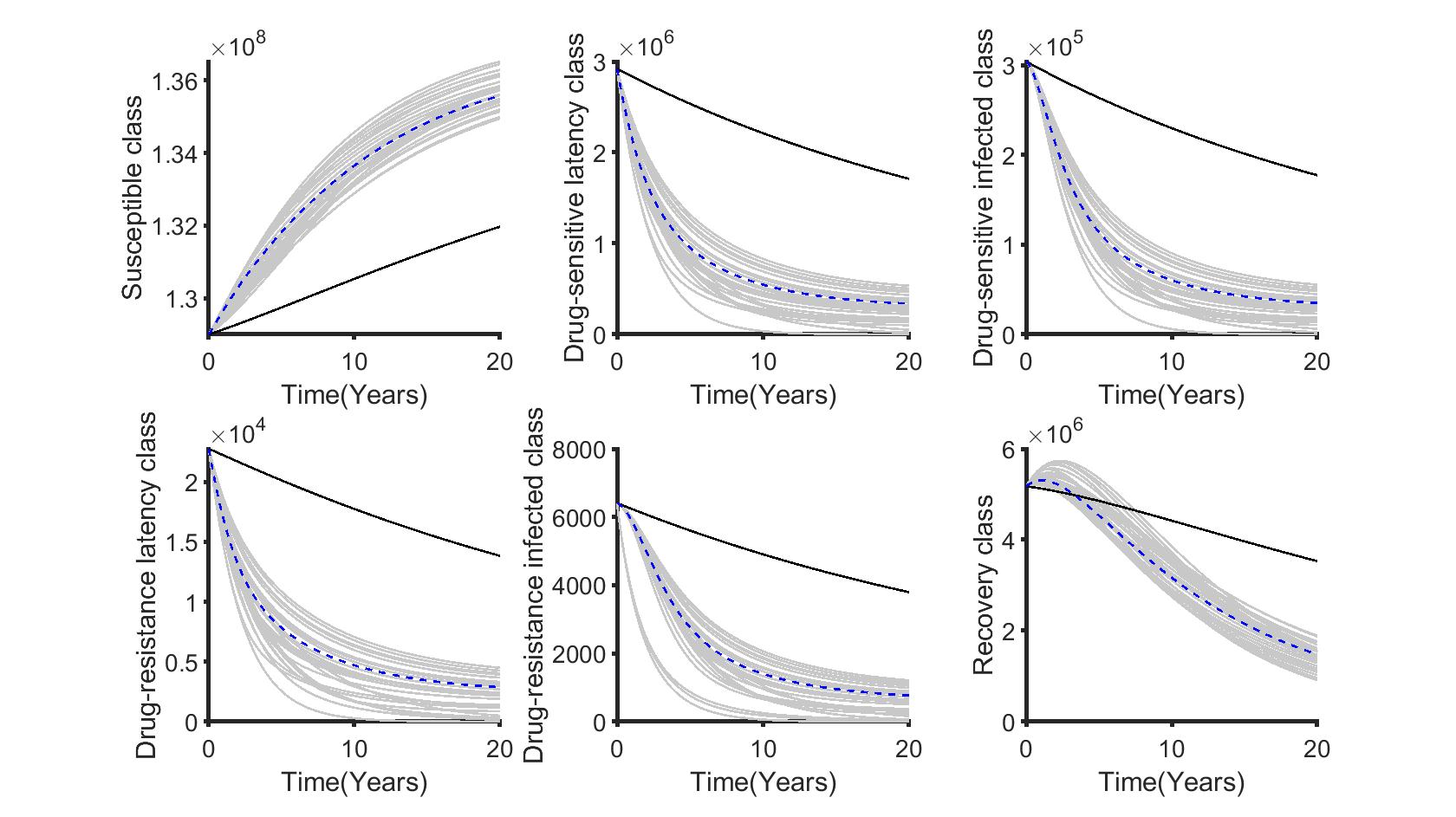
**

**S8 Fig (D).** The corresponding state variables of the combination of distancing control$(u_{1})$, latent case finding $(u_{2})$ and case holding $(u_{3})$ control strategy and considering the weighted cost $B_{2}$ is varied and $B_{1}{=B}_{3}=10^{5}=10^{6}=10^{7}$. The state variables with and without controls are plotted by grays and black lines respectively.

**
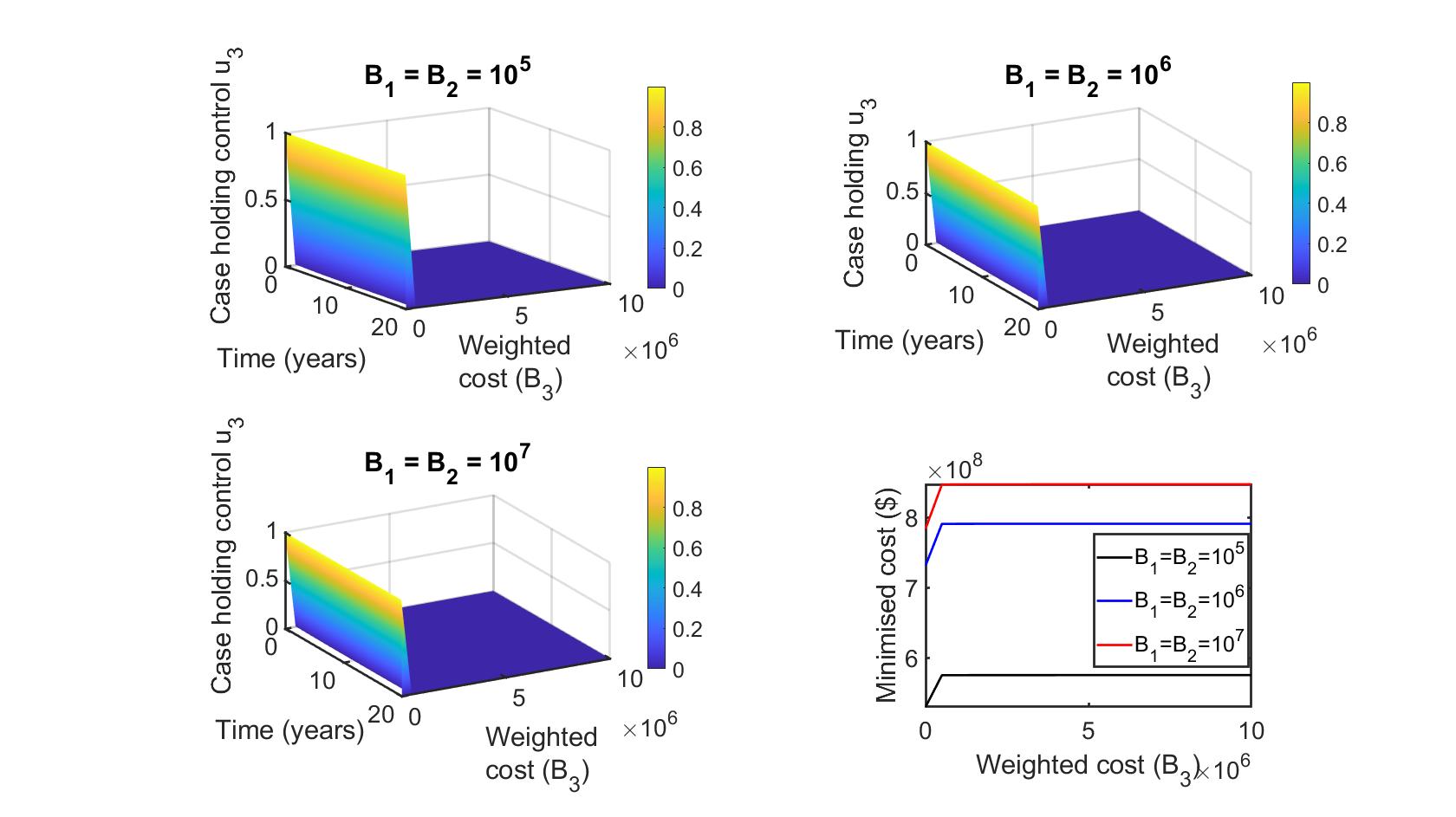
**

**S9 Fig (A).** Combination of distancing$\left( u_{1} \right)$, latent case finding$(u_{2})$ and case holding $(u_{3})$strategy, and considering case holding control $\left( u_{3} \right)$ strategy as a function of time and weighted cost$(B_{3})$. The weighted cost ${(B}_{1}\mathrm{and}B_{2})$ determined by three threshold values$B_{1}{=B}_{2}=10^{5}=10^{6}=10^{7}$.

**
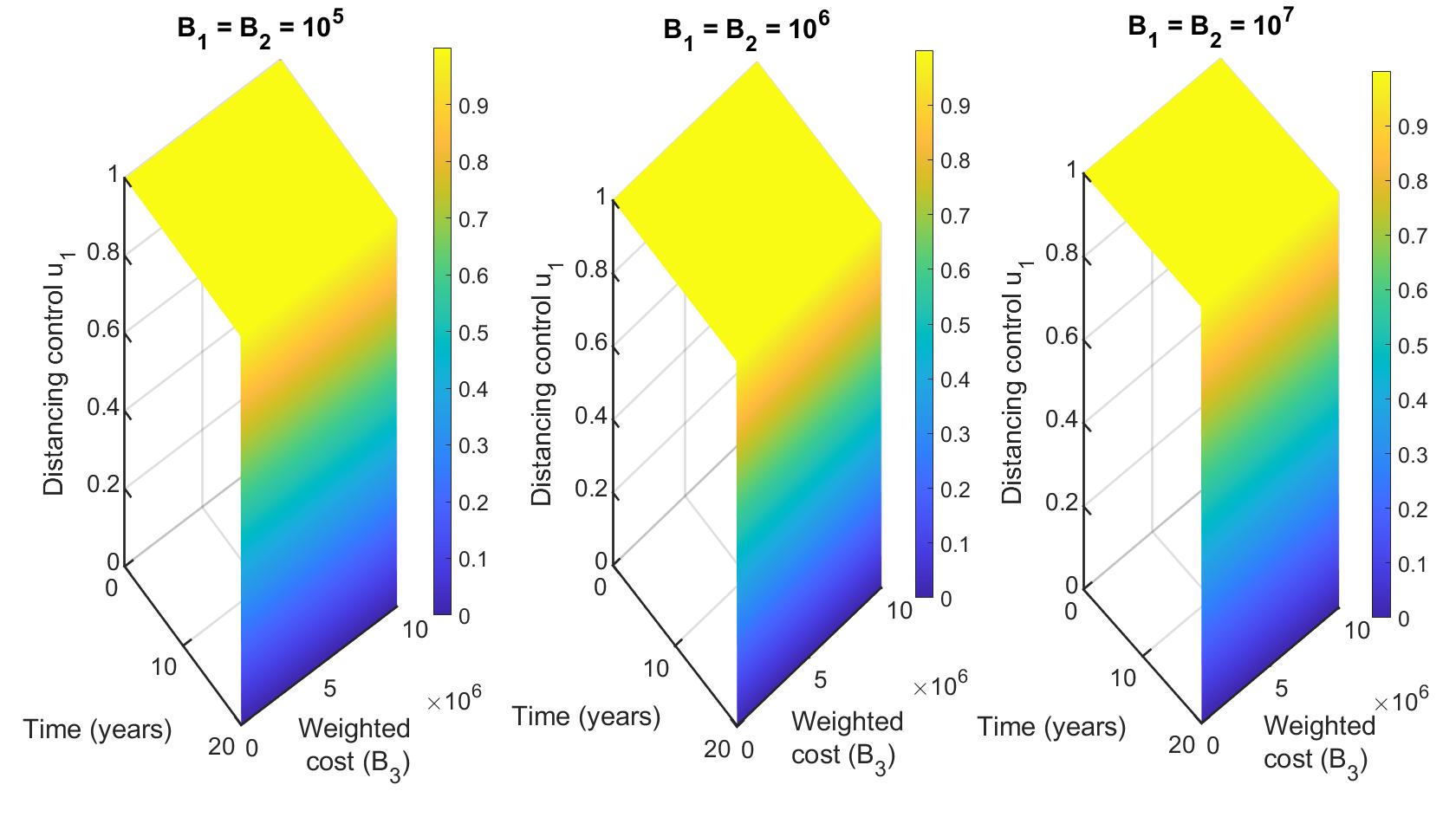
**

**S9 Fig (B).** Combination of distancing$\left( u_{1} \right)$, latent case finding$(u_{2})$ and case holding $(u_{3})$strategy, and considering distancing control $\left( u_{1} \right)$ strategy as a function of time and weighted cost$(B_{3})$. The weighted cost ${(B}_{1}\mathrm{and}B_{2})$ determined by three threshold values$B_{1}{=B}_{2}=10^{5}=10^{6}=10^{7}$.

**
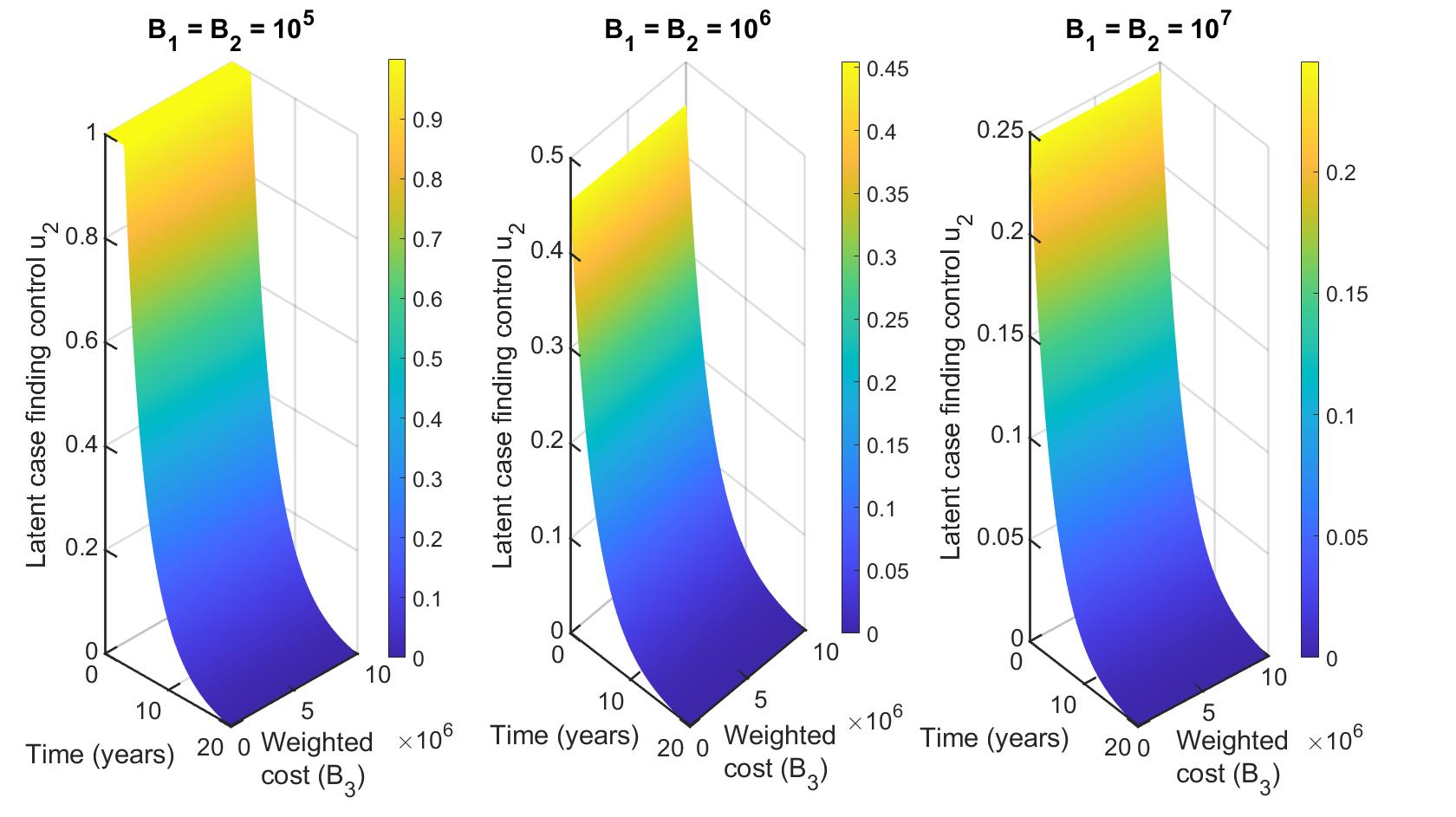
**

**S9 Fig (C).** Combination of distancing$\left( u_{1} \right)$, latent case finding$(u_{2})$ and case holding $(u_{3})$strategy, and considering latent case finding control $\left( u_{2} \right)$ strategy as a function of time and weighted cost$(B_{3})$. The weighted cost ${(B}_{1}\mathrm{and}B_{2})$ determined by three threshold values$B_{1}{=B}_{2}=10^{5}=10^{6}=10^{7}$.

**
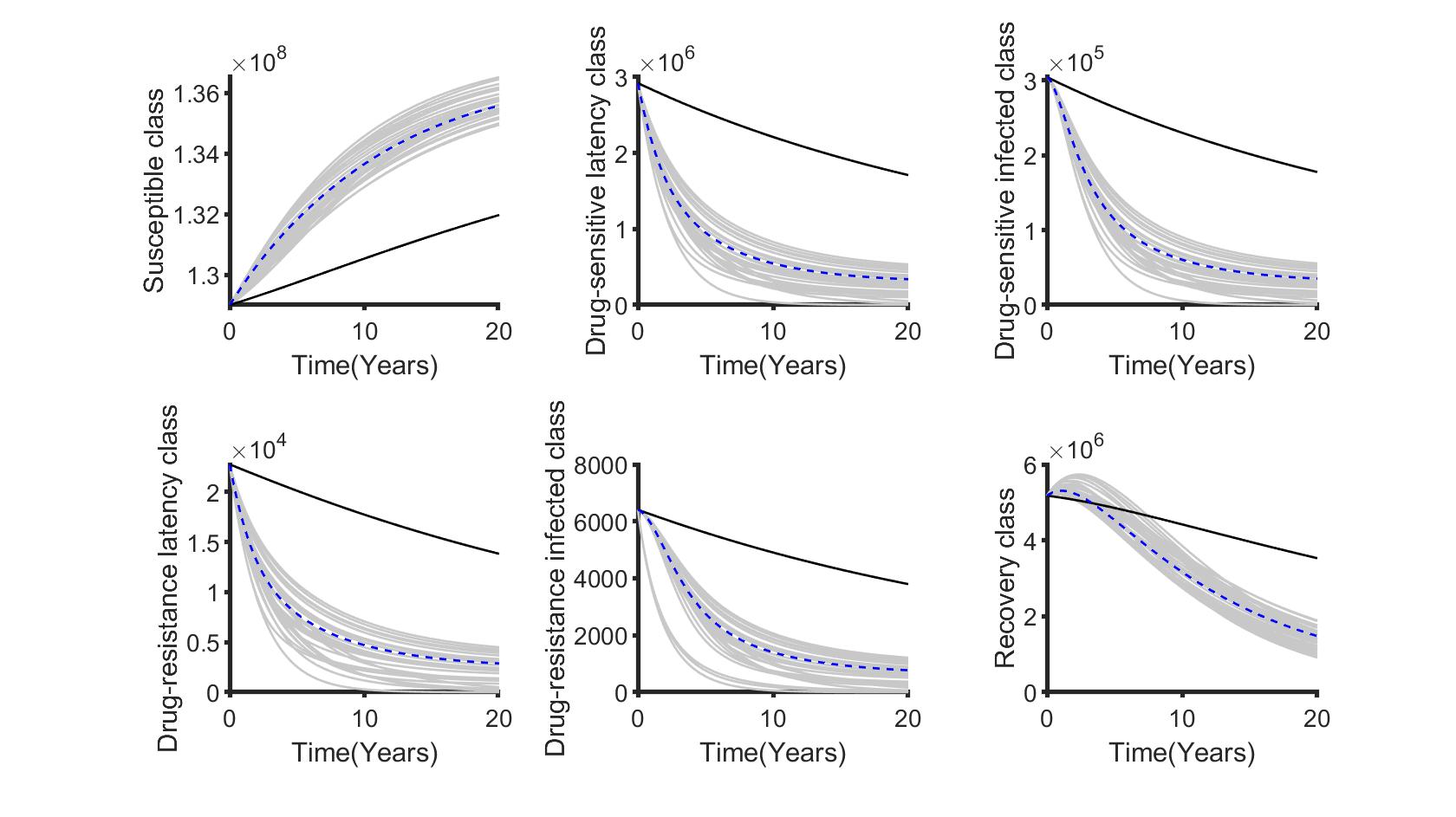
**

**S9 Fig (D).** The corresponding state variables of the combination of distancing control$(u_{1})$, latent case finding $(u_{2})$ and case holding $(u_{3})$ control strategy when the weighted cost $B_{3}$ is varied and $B_{1}{=B}_{2}=10^{5}=10^{6}=10^{7}$. The state variables with and without controls are plotted by grays and black lines respectively.

**
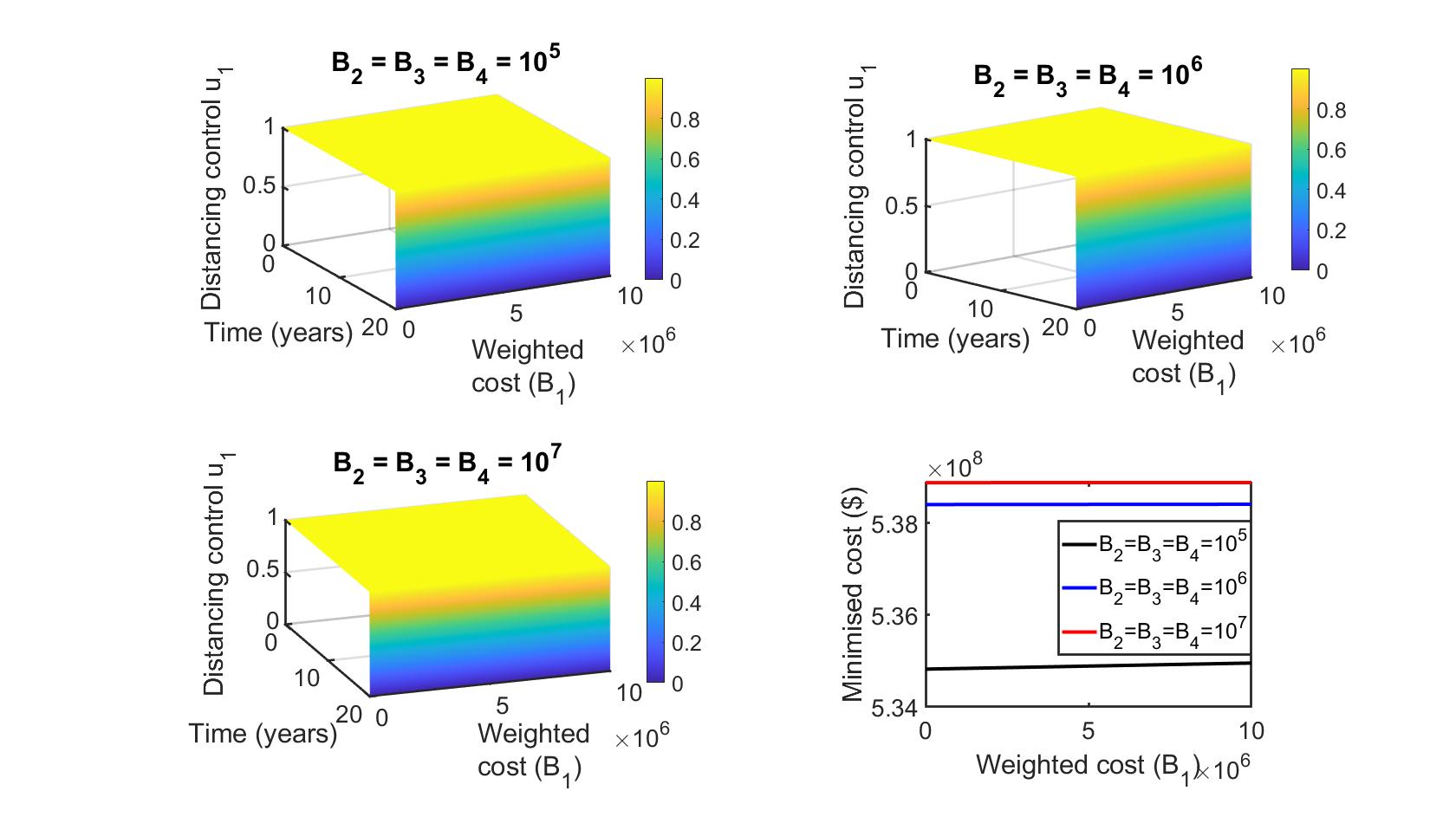
**

**S10 Fig (A).** Combination of distancing$\left( u_{1} \right)$, latent case finding$(u_{2})$, case holding $(u_{3})$ and active case finding control strategy, and considering distancing control $\left( u_{1} \right)$ strategy as a function of time and weighted cost$(B_{1})$. The weighted costs ${(B}_{2}, B_{3}\mathrm{and}B_{4})$ determined by three threshold values$B_{2}{=B}_{3}{=B}_{4}=10^{5}=10^{6}=10^{7}$.

**
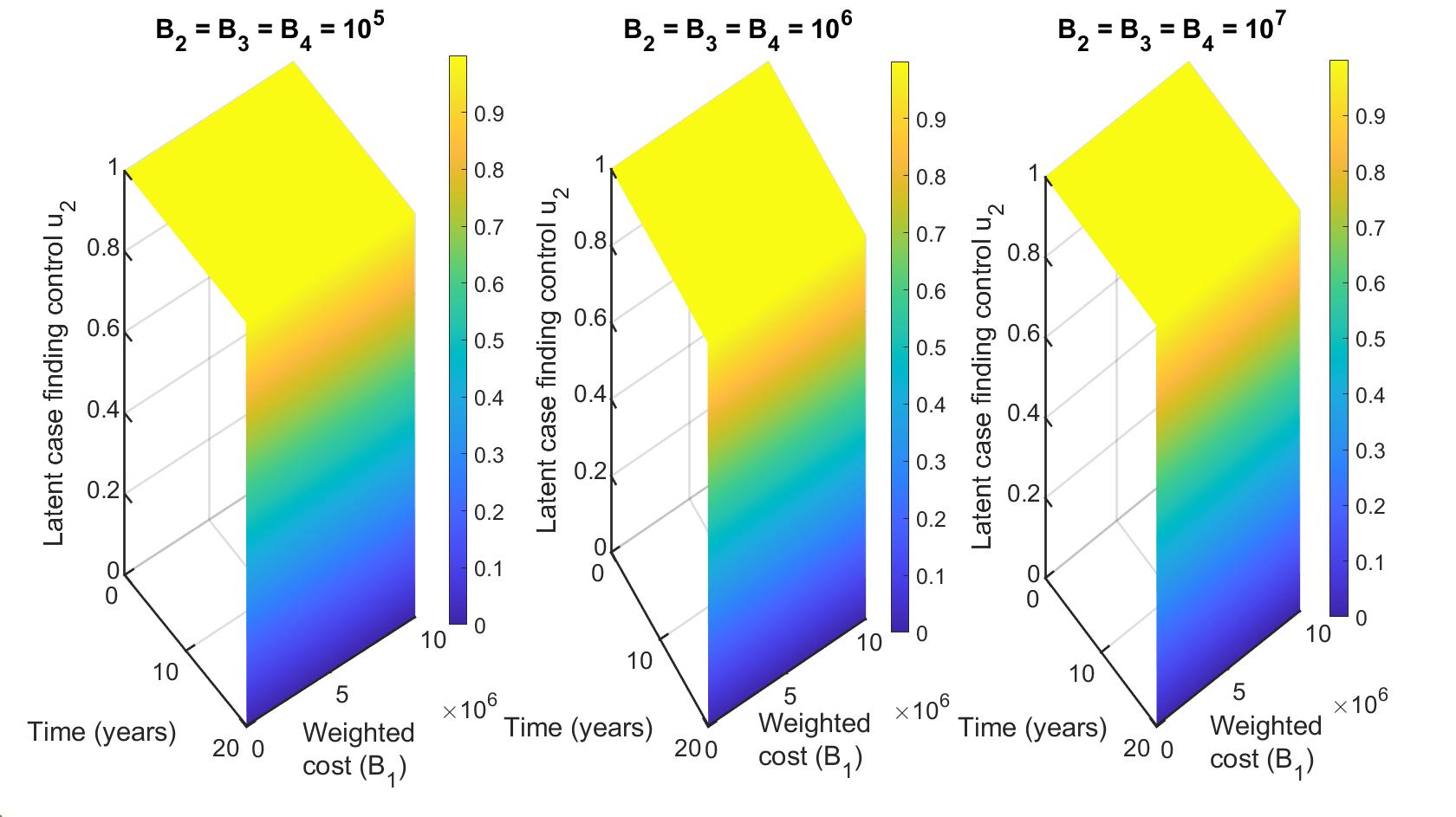
**

**S10 Fig (B).** Combination of distancing$\left( u_{1} \right)$, latent case finding$(u_{2})$, case holding $(u_{3})$ and active case finding control strategy, and considering latent case finding control $\left( u_{2} \right)$ strategy as a function of time and weighted cost$(B_{1})$. The weighted costs ${(B}_{2}, B_{3}\mathrm{and}B_{4})$ determined by three threshold values$B_{2}{=B}_{3}{=B}_{4}=10^{5}=10^{6}=10^{7}$.

**
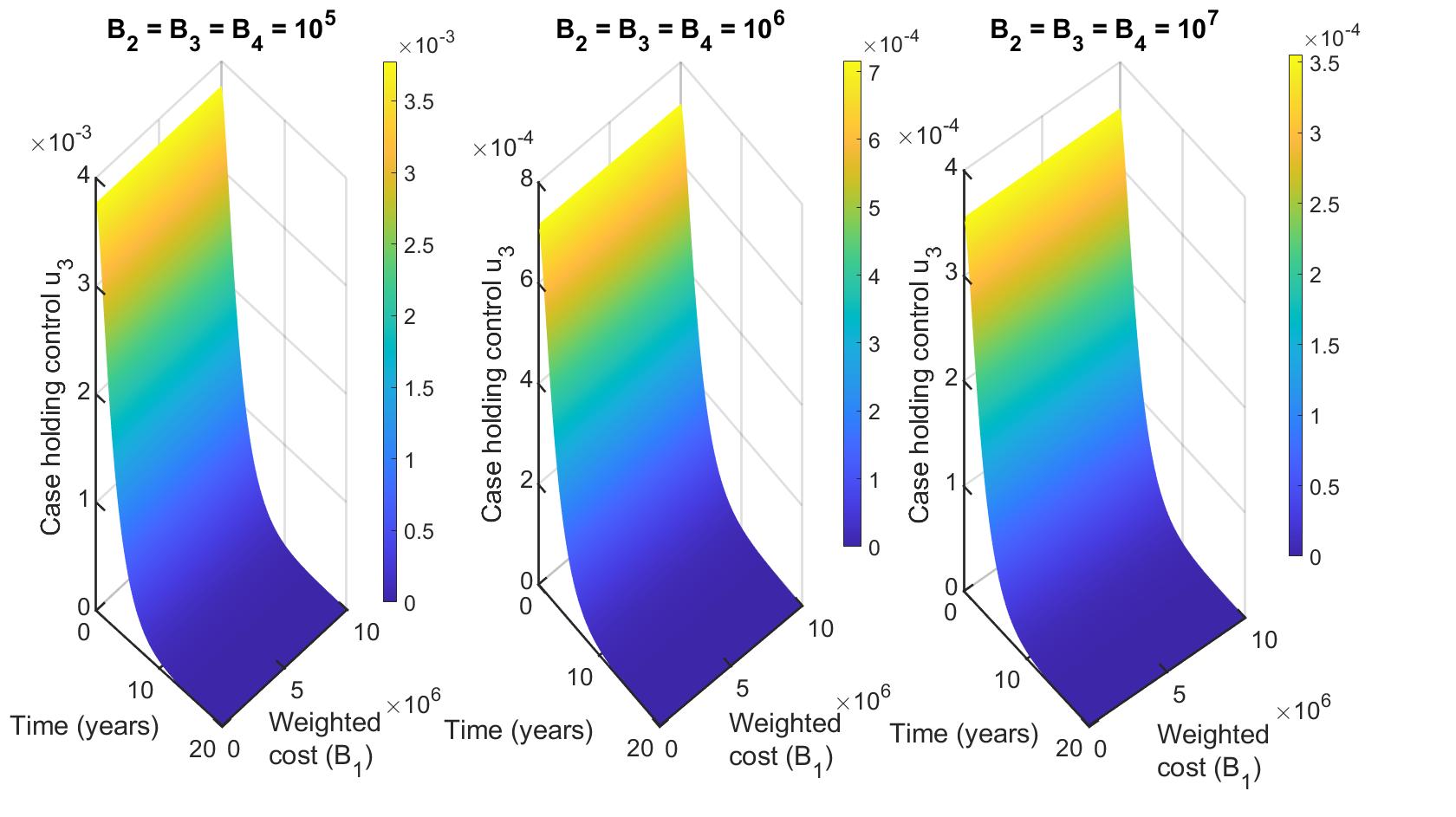
**

**S10 Fig (C).** Combination of distancing$\left( u_{1} \right)$, latent case finding$(u_{2})$, case holding $(u_{3})$ and active case finding control strategy, and considering case holding control $\left( u_{3} \right)$ strategy as a function of time and weighted cost$(B_{1})$. The weighted costs ${(B}_{2}, B_{3}\mathrm{and}B_{4})$ determined by three threshold values$B_{2}{=B}_{3}{=B}_{4}=10^{5}=10^{6}=10^{7}$.

**
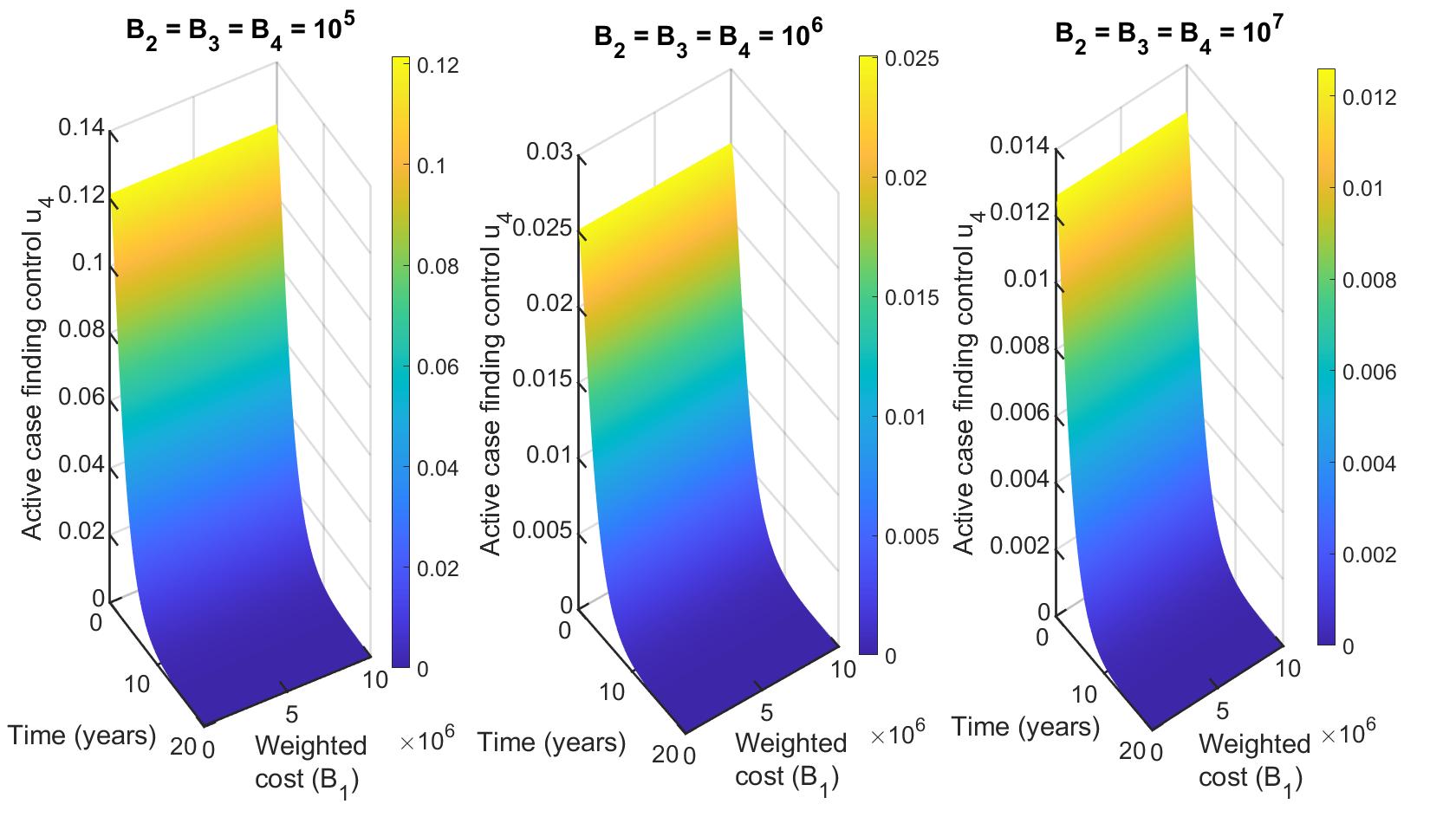
**

**S10 Fig (D).** Combination of distancing$\left( u_{1} \right)$, latent case finding$(u_{2})$, case holding $(u_{3})$ and active case finding control strategy, and considering active case finding control $\left( u_{4} \right)$ strategy as a function of time and weighted cost$(B_{1})$. The weighted costs ${(B}_{2}, B_{3}\mathrm{and}B_{4})$ determined by three threshold values$B_{2}{=B}_{3}{=B}_{4}=10^{5}=10^{6}=10^{7}$.

**
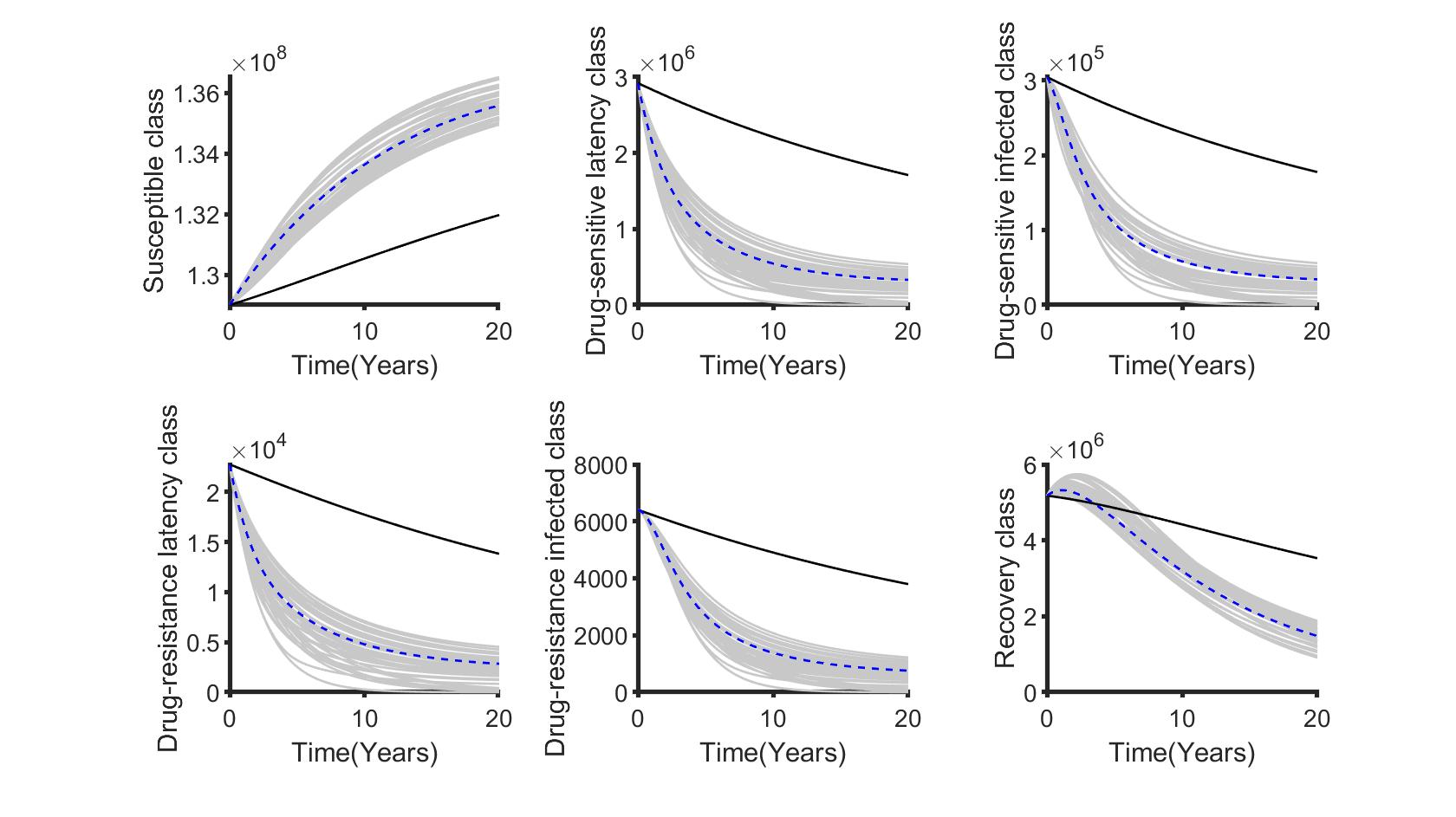
**

**S10 Fig (E).** The corresponding state variables of the combination of distancing control$(u_{1})$, latent case finding $(u_{2})$, case holding $(u_{3})$ and active case finding $(u_{4})$ control strategy when the weighted cost $B_{1}$ is varied and $B_{2}{=B}_{3}{=B}_{4}=10^{5}=10^{6}=10^{7}$. The state variables with and without controls are plotted by grays and black lines respectively.

**
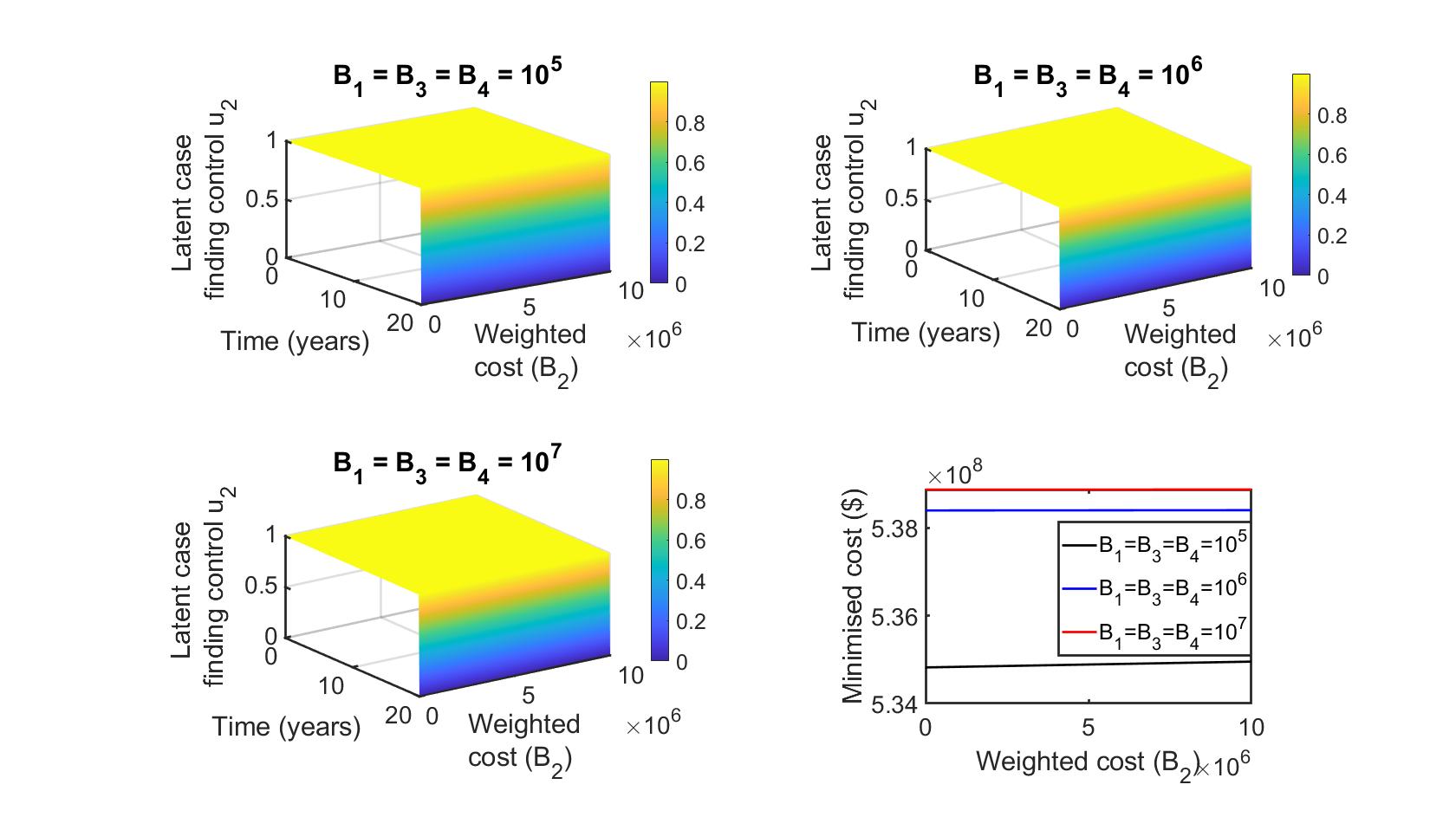
**

**S11 Fig (A).** Combination of distancing$\left( u_{1} \right)$, latent case finding$(u_{2})$, case holding $(u_{3})$ and active case finding control strategy, and considering latent case finding control $\left( u_{2} \right)$ strategy as a function of time and weighted cost$(B_{2})$. The weighted costs ${(B}_{1}, B_{3}\mathrm{and}B_{4})$ determined by three threshold values$B_{1}{=B}_{3}{=B}_{4}=10^{5}=10^{6}=10^{7}$.

**
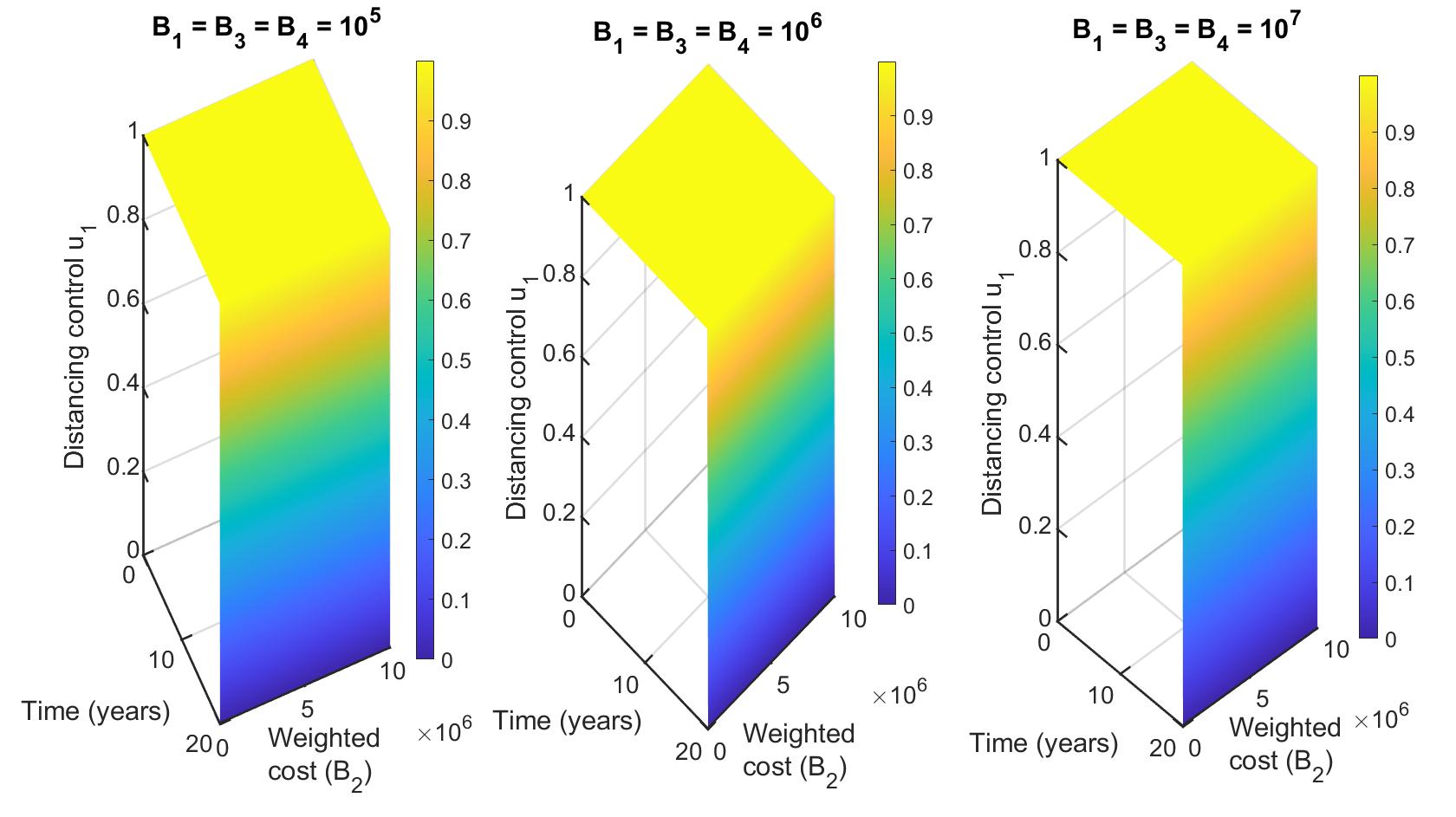
S11 Fig (B).** Combination of distancing$\left( u_{1} \right)$, latent case finding$(u_{2})$, case holding $(u_{3})$ and active case finding control strategy, and considering distancing control $\left( u_{1} \right)$ strategy as a function of time and weighted cost$(B_{2})$. The weighted costs ${(B}_{1}, B_{3}\mathrm{and}B_{4})$ determined by three threshold values$B_{1}{=B}_{3}{=B}_{4}=10^{5}=10^{6}=10^{7}$.

**
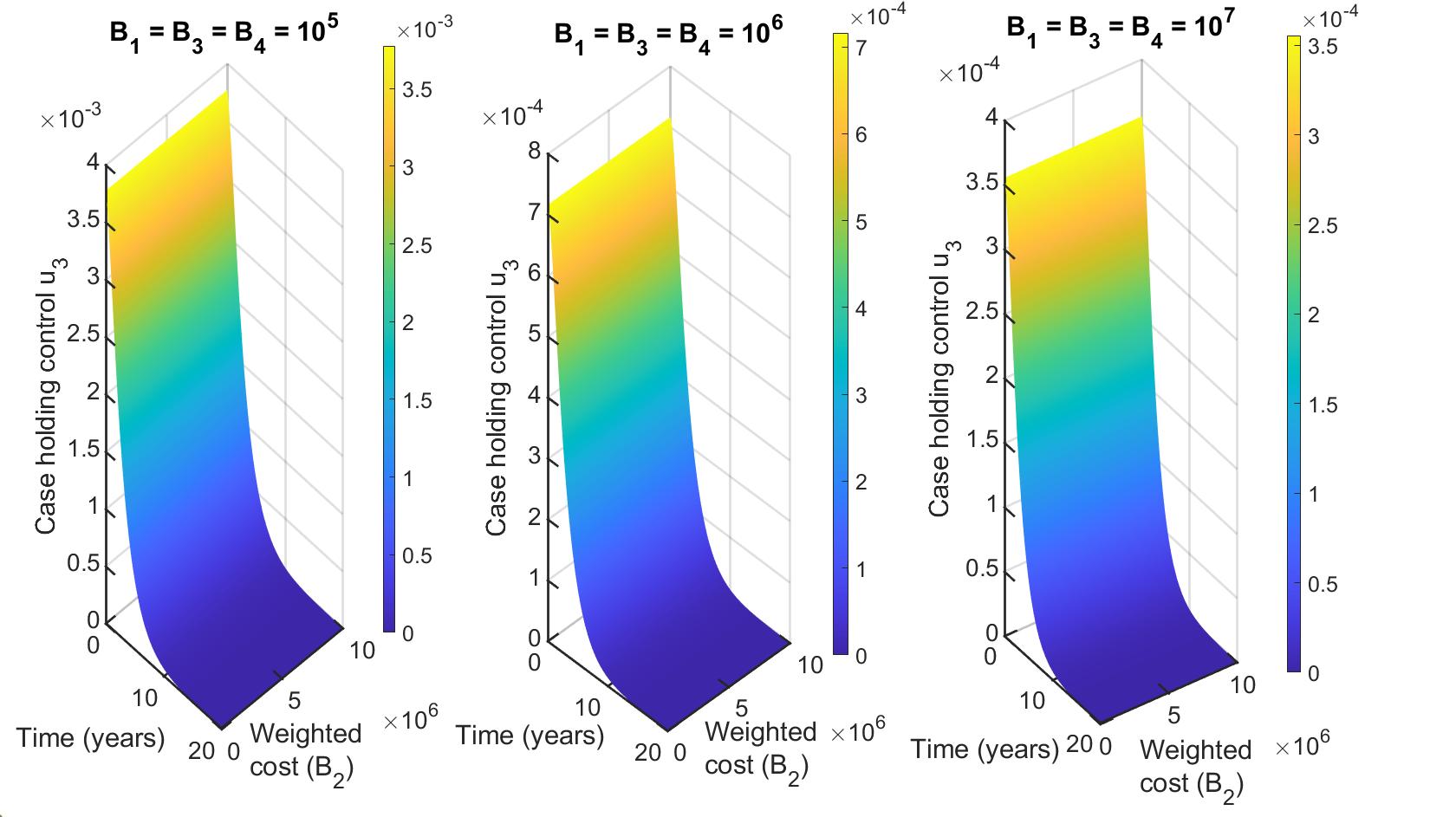
**

**S11 Fig (C).** Combination of distancing$\left( u_{1} \right)$, latent case finding$(u_{2})$, case holding $(u_{3})$ and active case finding control strategy, and considering case holding control $\left( u_{3} \right)$ strategy as a function of time and weighted cost$(B_{2})$. The weighted costs ${(B}_{1}, B_{3}\mathrm{and}B_{4})$ determined by three threshold values$B_{1}{=B}_{3}{=B}_{4}=10^{5}=10^{6}=10^{7}$.

**
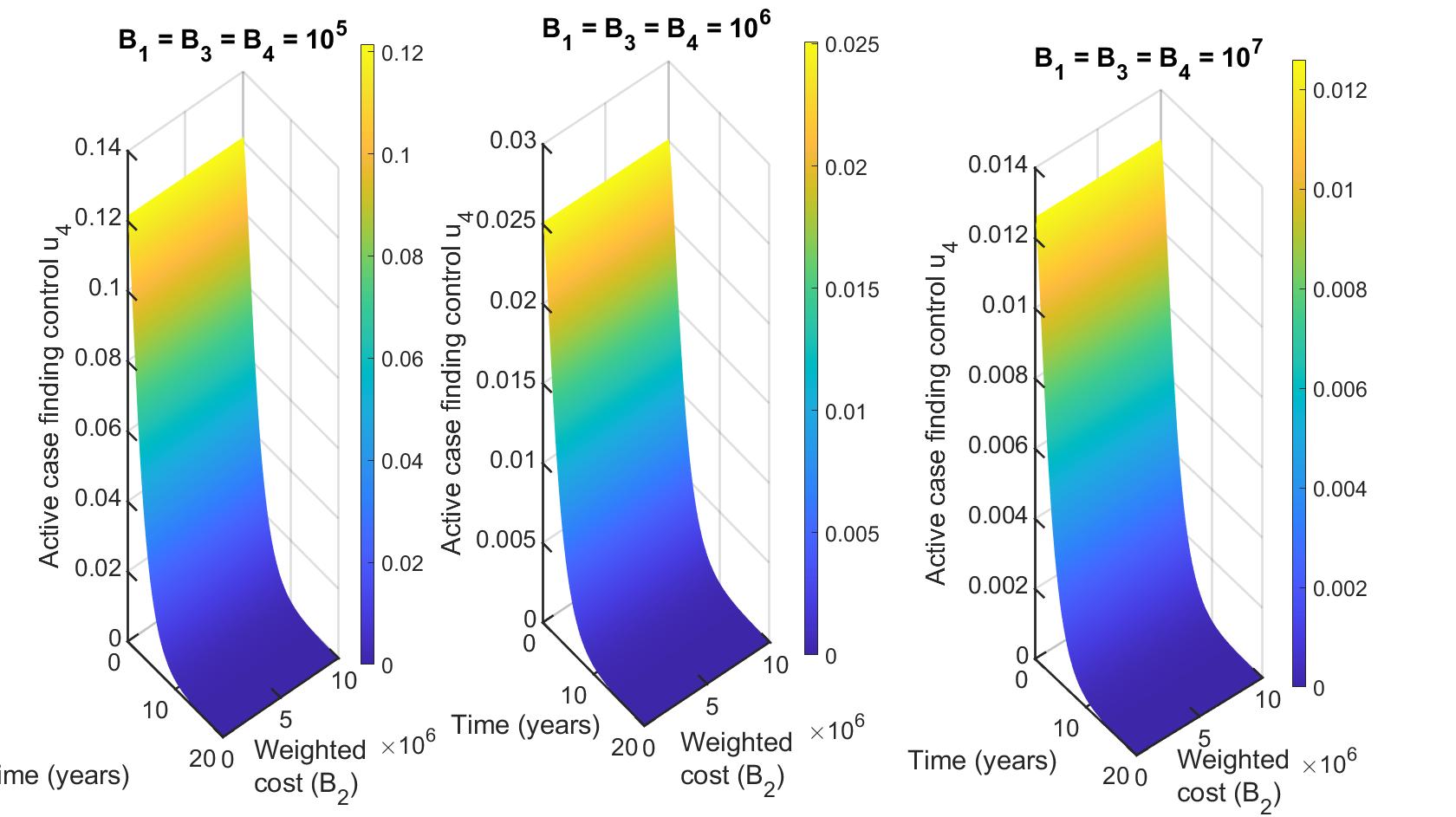
**

**S11 Fig (D).** Combination of distancing$\left( u_{1} \right)$, latent case finding$(u_{2})$, case holding $(u_{3})$ and active case finding control strategy, and considering active case finding control $\left( u_{4} \right)$ strategy as a function of time and weighted cost$(B_{2})$. The weighted costs ${(B}_{1}, B_{3}\mathrm{and}B_{4})$ determined by three threshold values$B_{1}{=B}_{3}{=B}_{4}=10^{5}=10^{6}=10^{7}$.

**
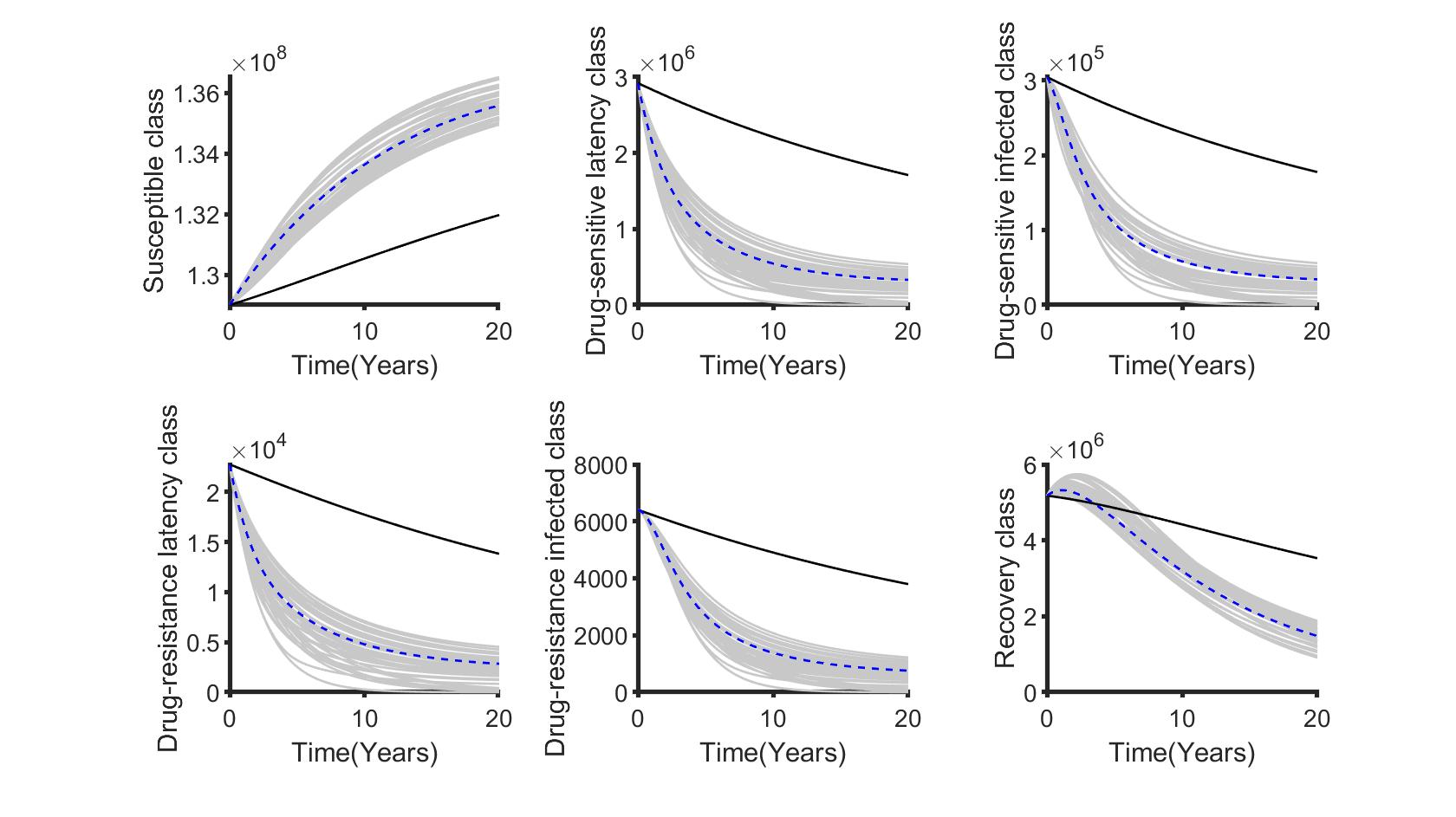
**

**S11 Fig (E).** The corresponding state variables of the combination of distancing control$(u_{1})$, latent case finding $(u_{2})$, case holding $(u_{3})$ and active case finding $(u_{4})$ control strategy when the weighted cost $B_{2}$ is varied and $B_{1}{=B}_{3}{=B}_{4}=10^{5}=10^{6}=10^{7}$. The state variables with and without controls are plotted by grays and black lines respectively.

**
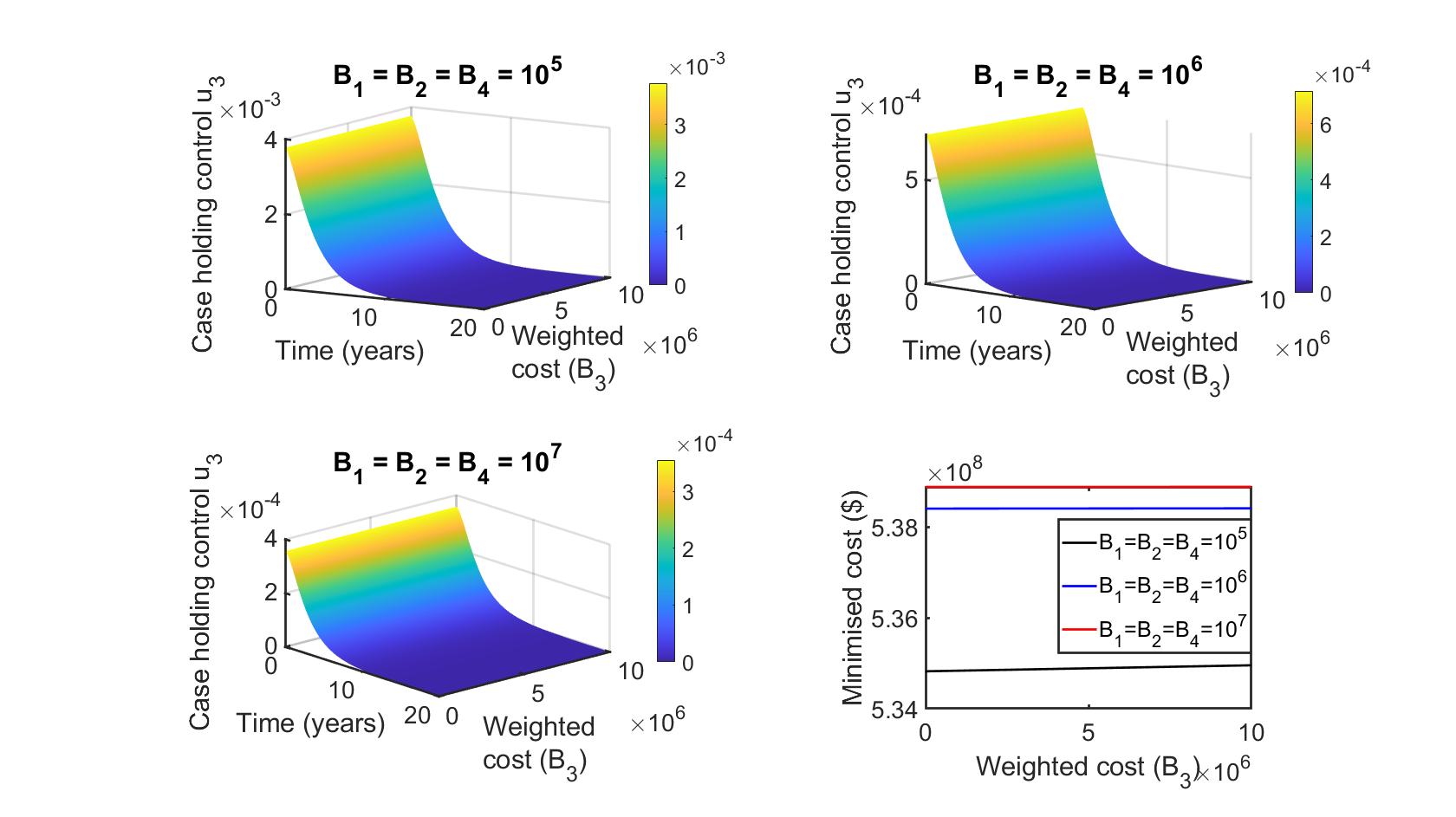
S12 Fig (A).** Combination of distancing$\left( u_{1} \right)$, latent case finding$(u_{2})$, case holding $(u_{3})$ and active case finding control strategy, and considering case holding control $\left( u_{3} \right)$ strategy as a function of time and weighted cost$(B_{3})$. The weighted costs ${(B}_{1}, B_{2}\mathrm{and}B_{4})$ determined by three threshold values$B_{1}{=B}_{2}{=B}_{4}=10^{5}=10^{6}=10^{7}$.

**
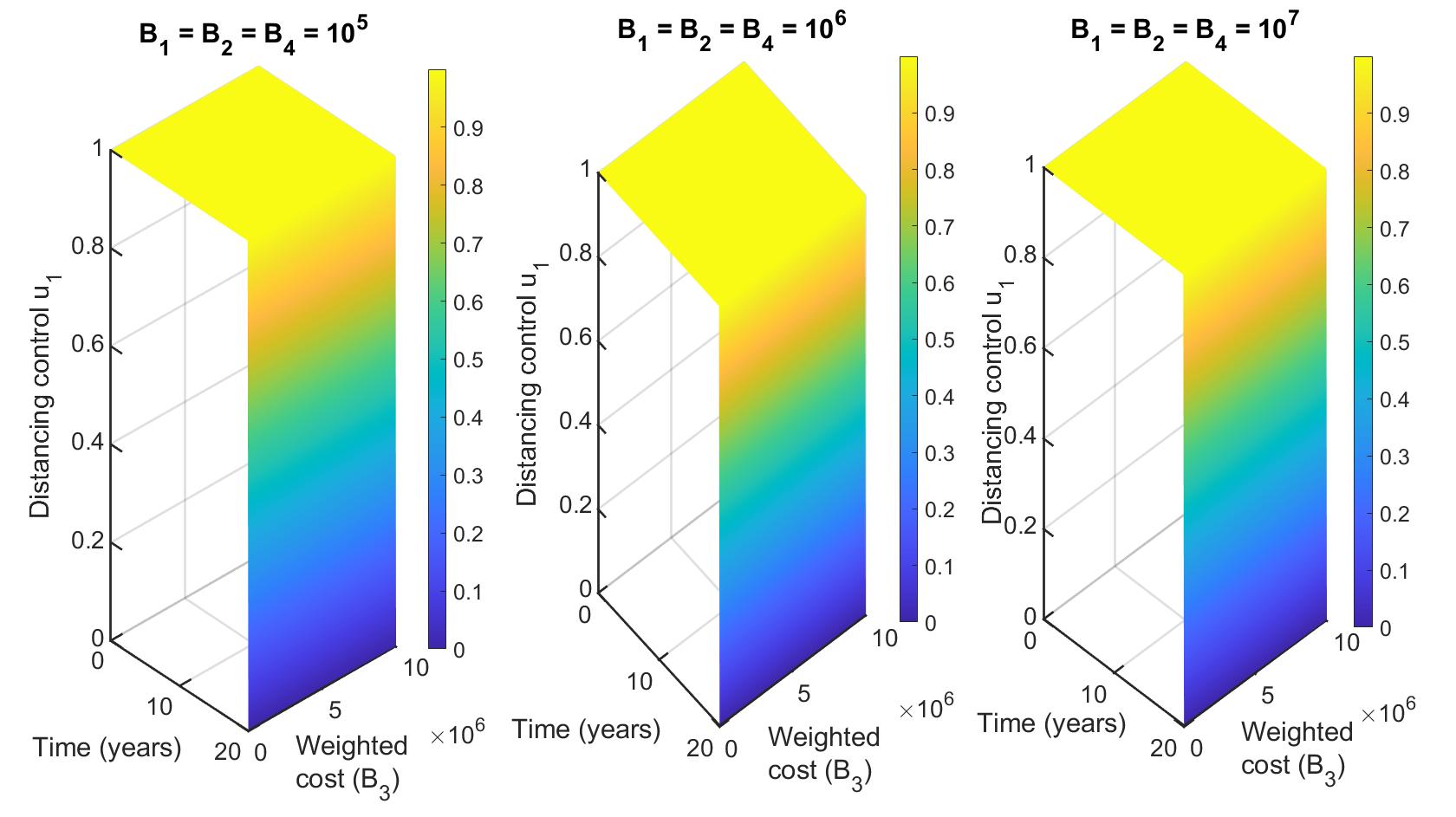
**

**S12 Fig (B).** Combination of distancing$\left( u_{1} \right)$, latent case finding$(u_{2})$, case holding $(u_{3})$ and active case finding control strategy, and considering distancing control $\left( u_{1} \right)$ strategy as a function of time and weighted cost$(B_{3})$. The weighted costs ${(B}_{1}, B_{2}\mathrm{and}B_{4})$ determined by three threshold values$B_{1}{=B}_{2}{=B}_{4}=10^{5}=10^{6}=10^{7}$.

**
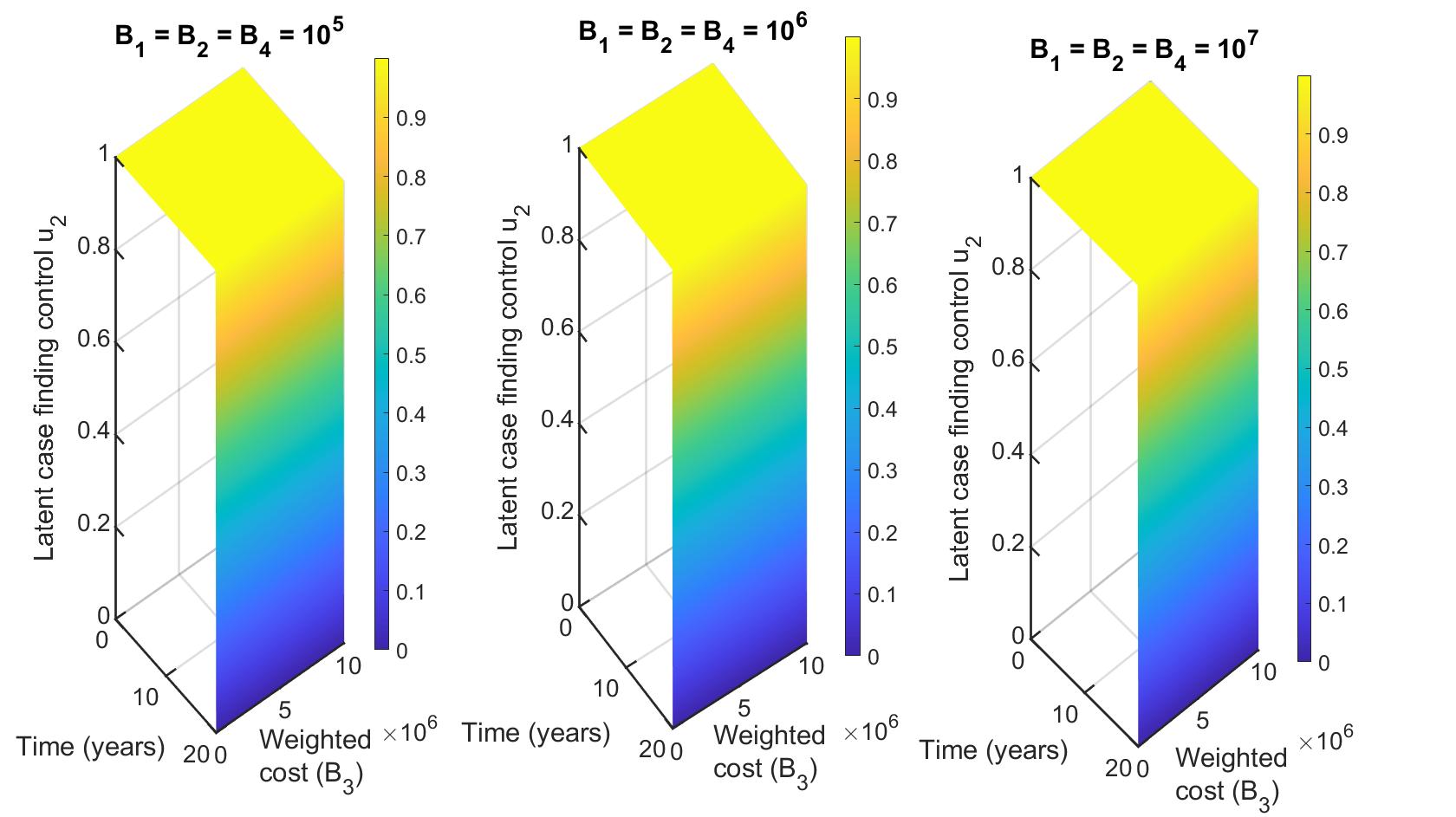
**

**S12 Fig (C).** Combination of distancing$\left( u_{1} \right)$, latent case finding$(u_{2})$, case holding $(u_{3})$ and active case finding control strategy, and considering latent case finding control $\left( u_{2} \right)$ strategy as a function of time and weighted cost$(B_{3})$. The weighted costs ${(B}_{1}, B_{2}\mathrm{and}B_{4})$ determined by three threshold values$B_{1}{=B}_{2}{=B}_{4}=10^{5}=10^{6}=10^{7}$.

**
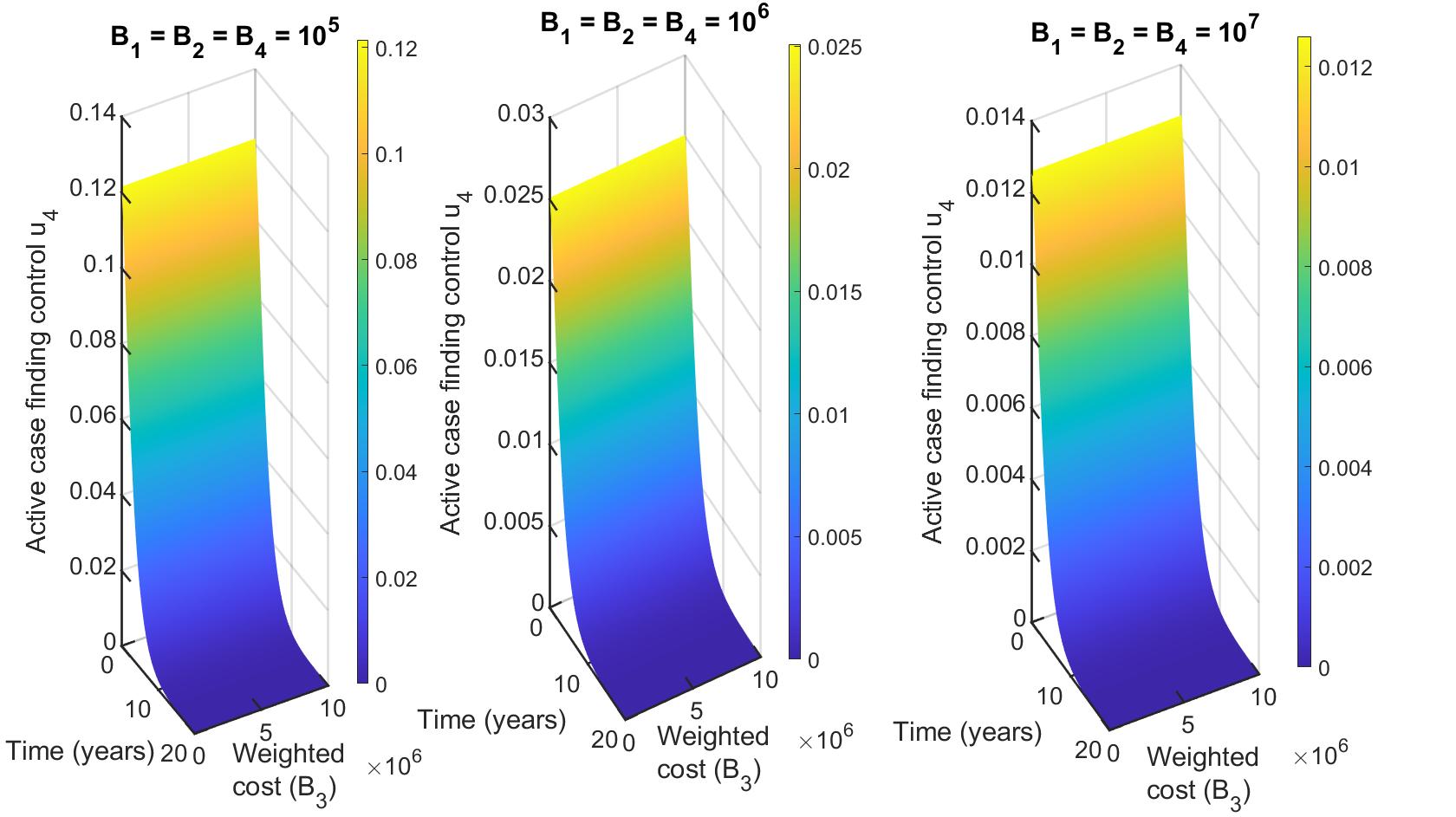
**

**S12 Fig (D).** Combination of distancing$\left( u_{1} \right)$, latent case finding$(u_{2})$, case holding $(u_{3})$ and active case finding control strategy, and considering active case finding control $\left( u_{4} \right)$ strategy as a function of time and weighted cost$(B_{3})$. The weighted costs ${(B}_{1}, B_{2}\mathrm{and}B_{4})$ determined by three threshold values$B_{1}{=B}_{2}{=B}_{4}=10^{5}=10^{6}=10^{7}$.

**
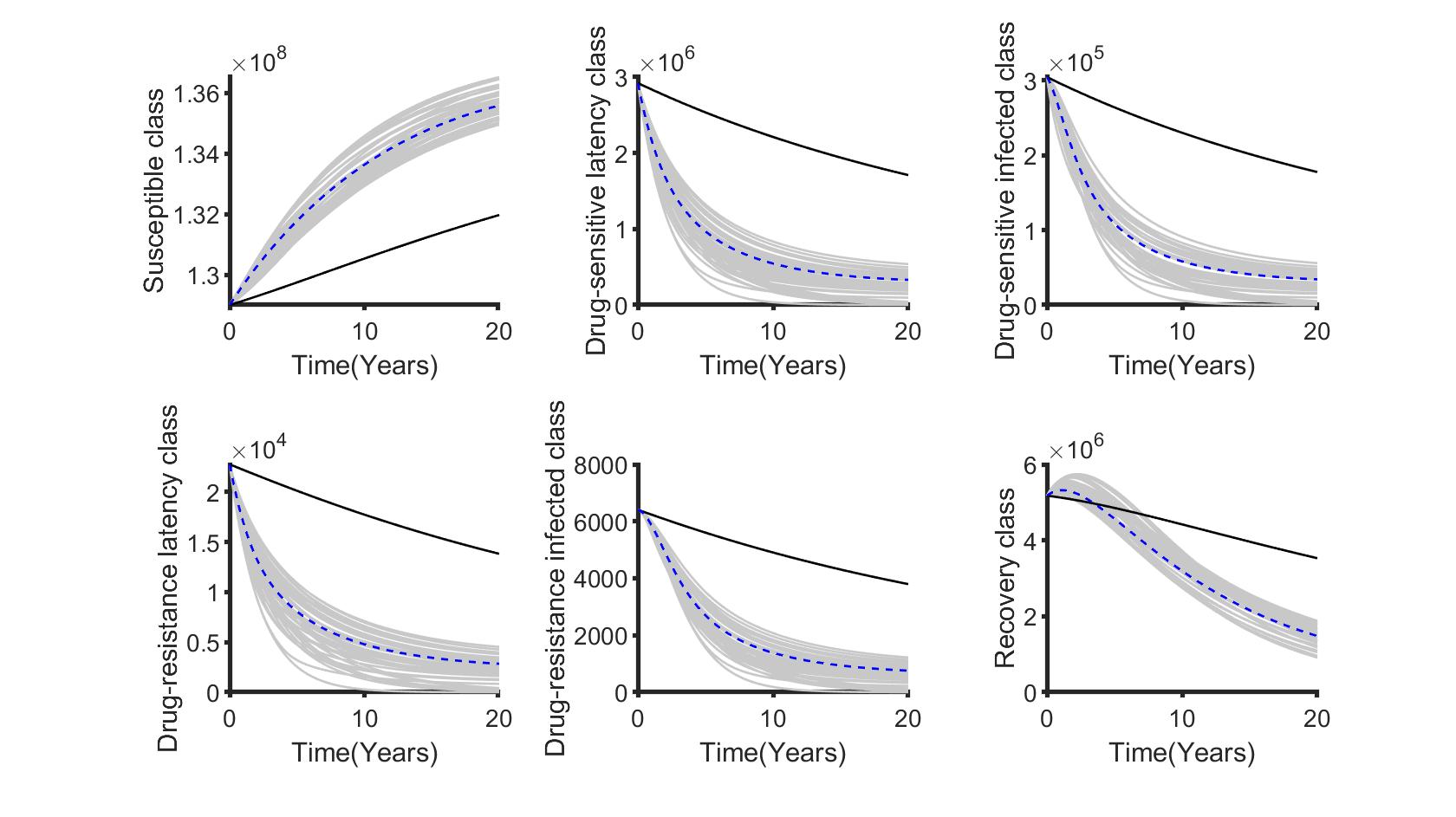
S12 Fig (E).** The corresponding state variables of the combination of distancing control$(u_{1})$, latent case finding $(u_{2})$, case holding $(u_{3})$ and active case finding $(u_{4})$ control strategy when the weighted cost $B_{3}$ is varied and $B_{1}{=B}_{2}{=B}_{4}=10^{5}=10^{6}=10^{7}$. The state variables with and without controls are plotted by grays and black lines respectively.

**
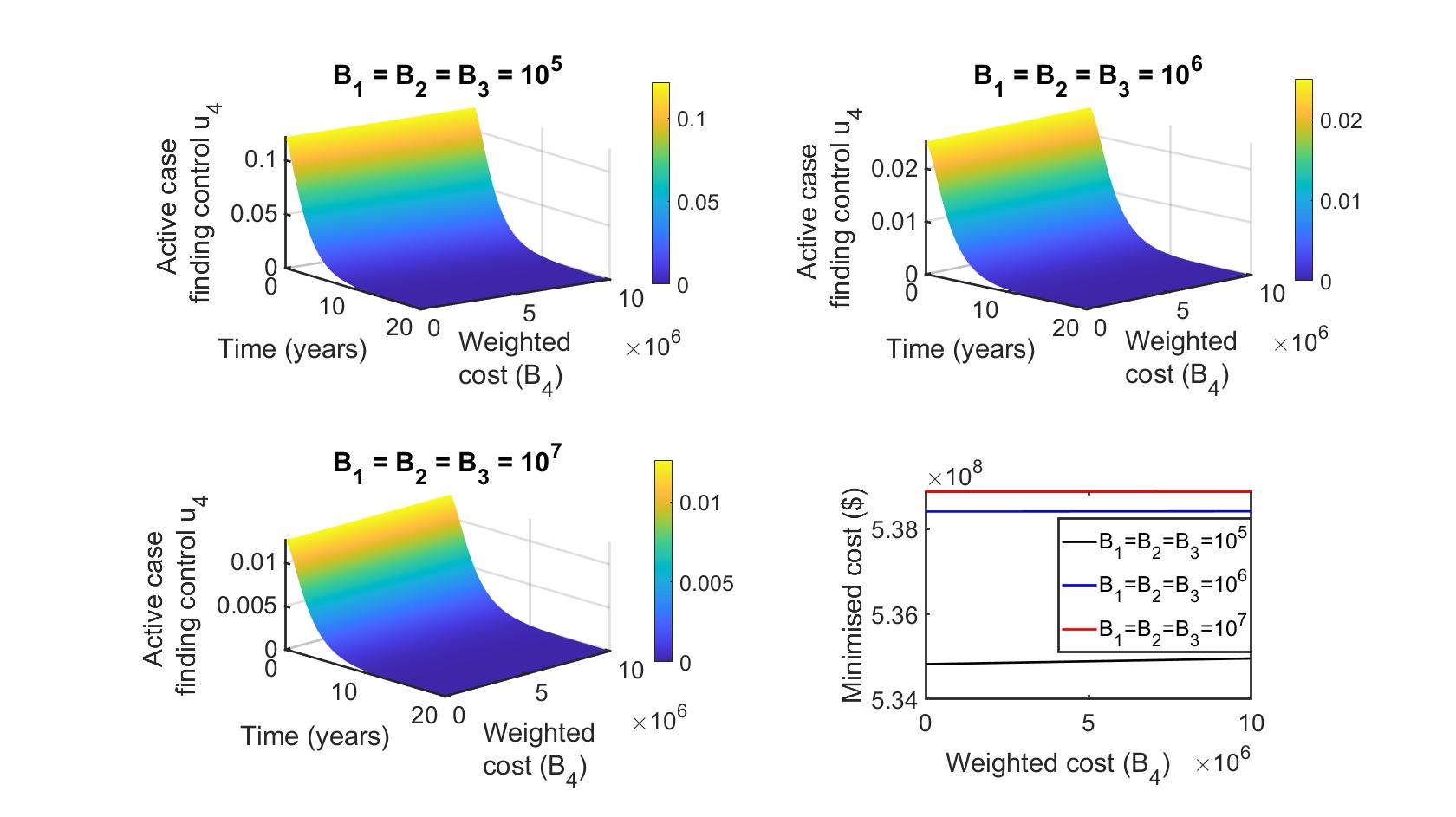
**

**S13 Fig (A).** Combination of distancing$\left( u_{1} \right)$, latent case finding$(u_{2})$, case holding $(u_{3})$ and active case finding control strategy, and considering active case finding control $\left( u_{1} \right)$ strategy as a function of time and weighted cost$(B_{4})$. The weighted costs ${(B}_{1}, B_{2}\mathrm{and}B_{3})$ determined by three threshold values$B_{1}{=B}_{2}{=B}_{3}=10^{5}=10^{6}=10^{7}$.

**
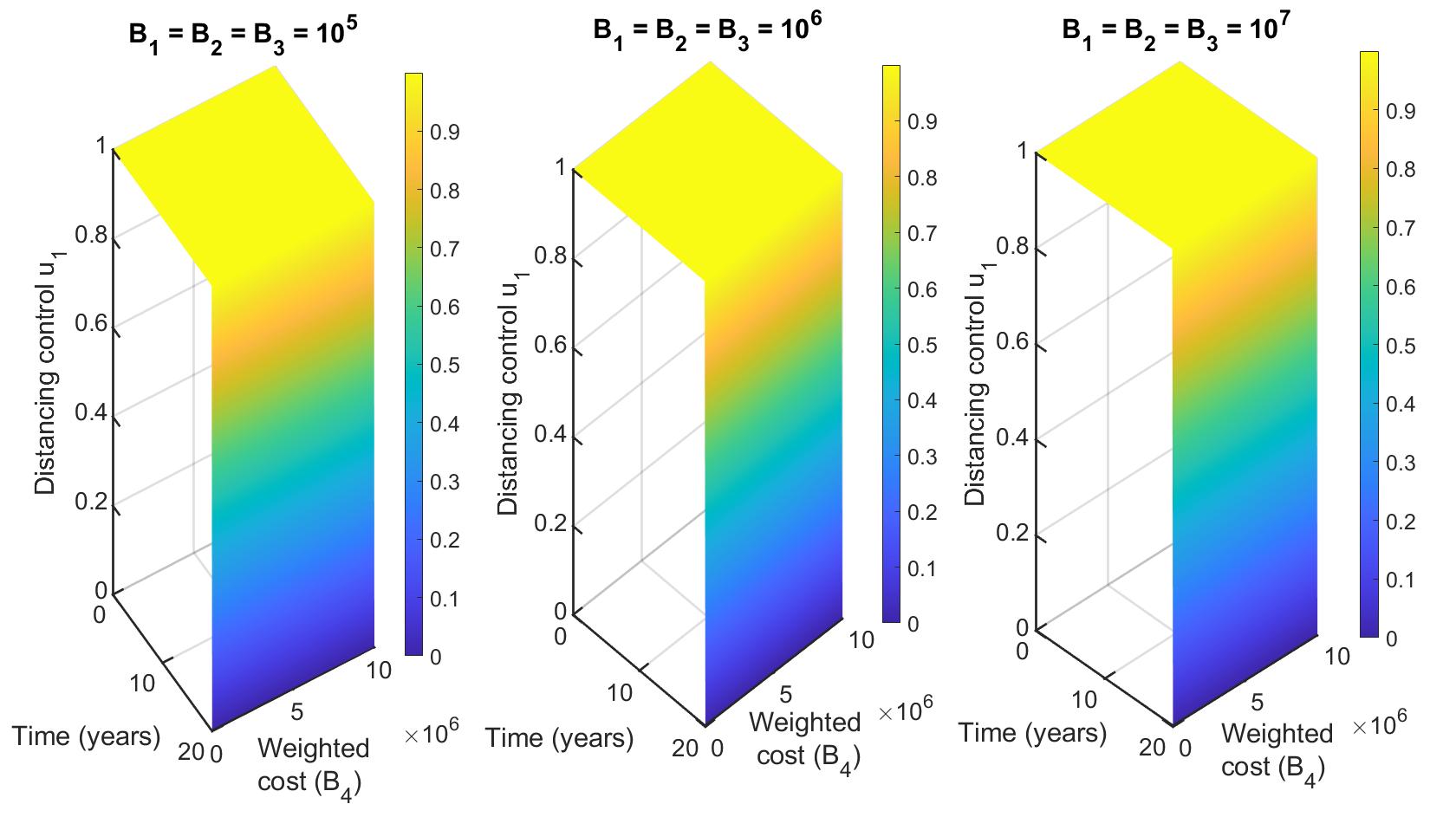
**

**S13 Fig (B).** Combination of distancing$\left( u_{1} \right)$, latent case finding$(u_{2})$, case holding $(u_{3})$ and active case finding control strategy, and considering distancing control $\left( u_{1} \right)$ strategy as a function of time and weighted cost$(B_{4})$. The weighted costs ${(B}_{1}, B_{2}\mathrm{and}B_{3})$ determined by three threshold values$B_{1}{=B}_{2}{=B}_{3}=10^{5}=10^{6}=10^{7}$.

**
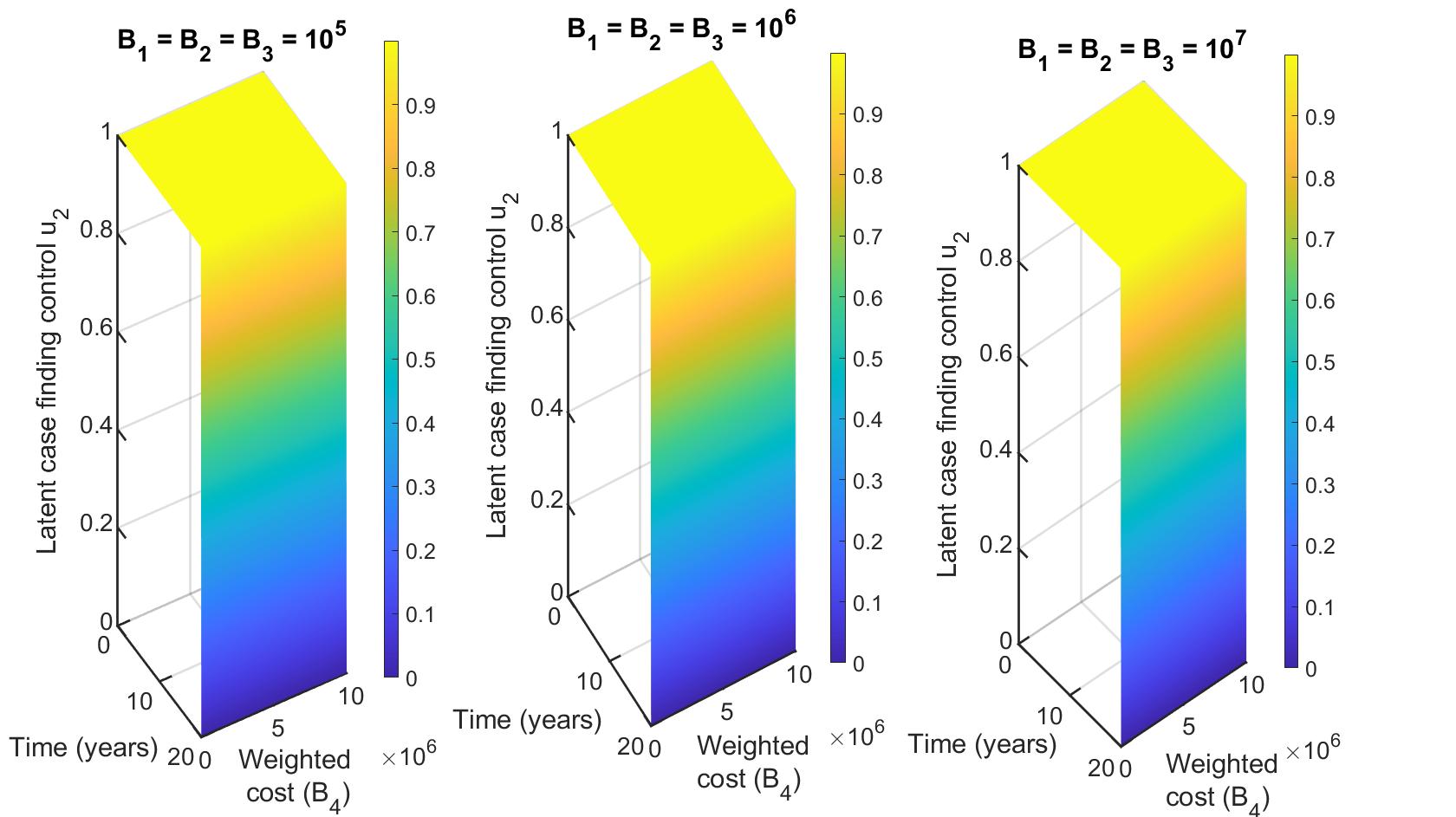
**

**S13 Fig (C).** Combination of distancing$\left( u_{1} \right)$, latent case finding$(u_{2})$, case holding $(u_{3})$ and active case finding control strategy, and considering latent case finding control $\left( u_{2} \right)$ strategy as a function of time and weighted cost$(B_{4})$. The weighted costs ${(B}_{1}, B_{2}\mathrm{and}B_{3})$ determined by three threshold values$B_{1}{=B}_{2}{=B}_{3}=10^{5}=10^{6}=10^{7}$.

**
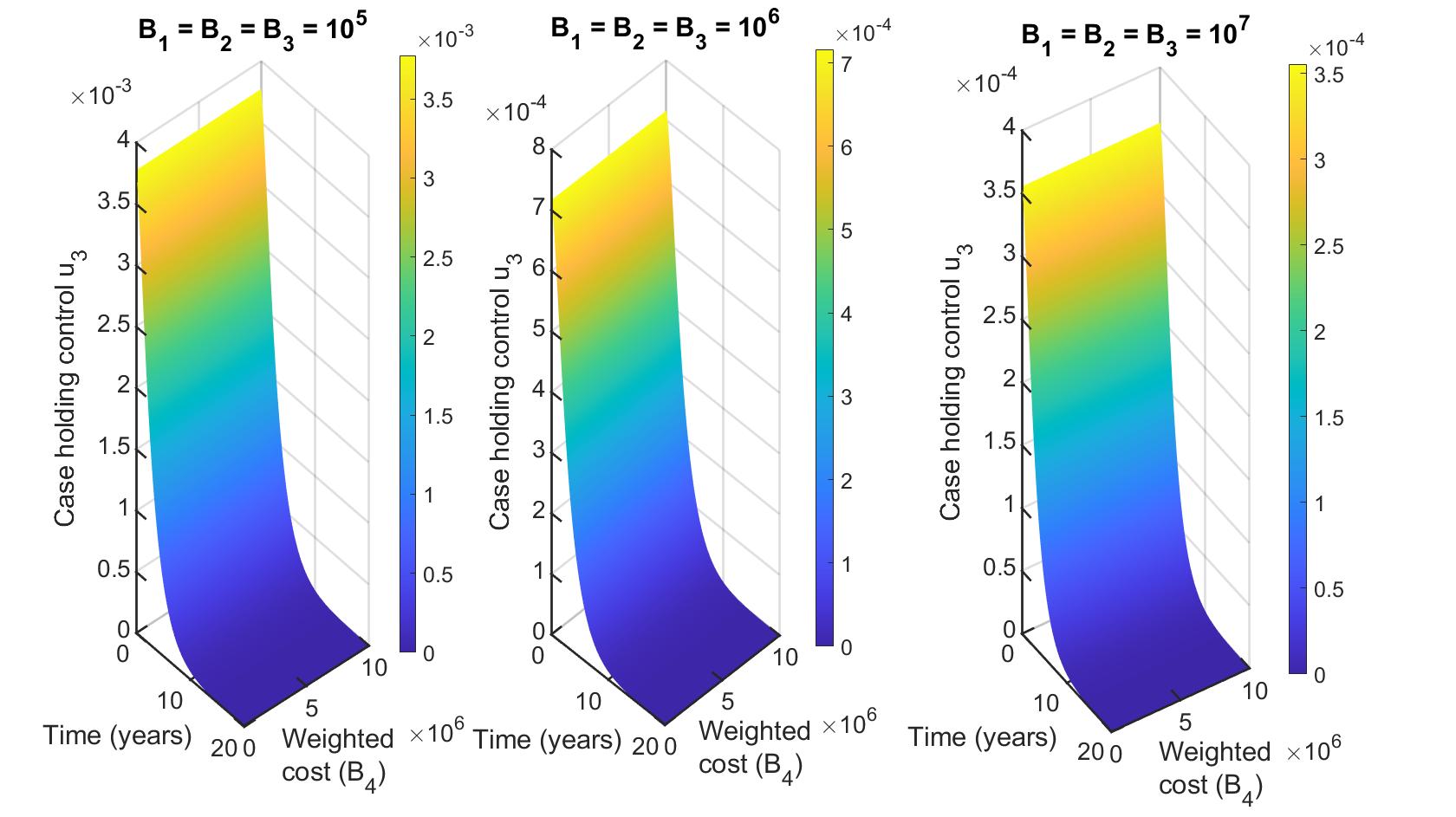
**

**S13 Fig (D).** Combination of distancing$\left( u_{1} \right)$, latent case finding$(u_{2})$, case holding $(u_{3})$ and active case finding control strategy, and considering case holding control $\left( u_{3} \right)$ strategy as a function of time and weighted cost$(B_{4})$. The weighted costs ${(B}_{1}, B_{2}\mathrm{and}B_{3})$ determined by three threshold values$B_{1}{=B}_{2}{=B}_{3}=10^{5}=10^{6}=10^{7}$.

**
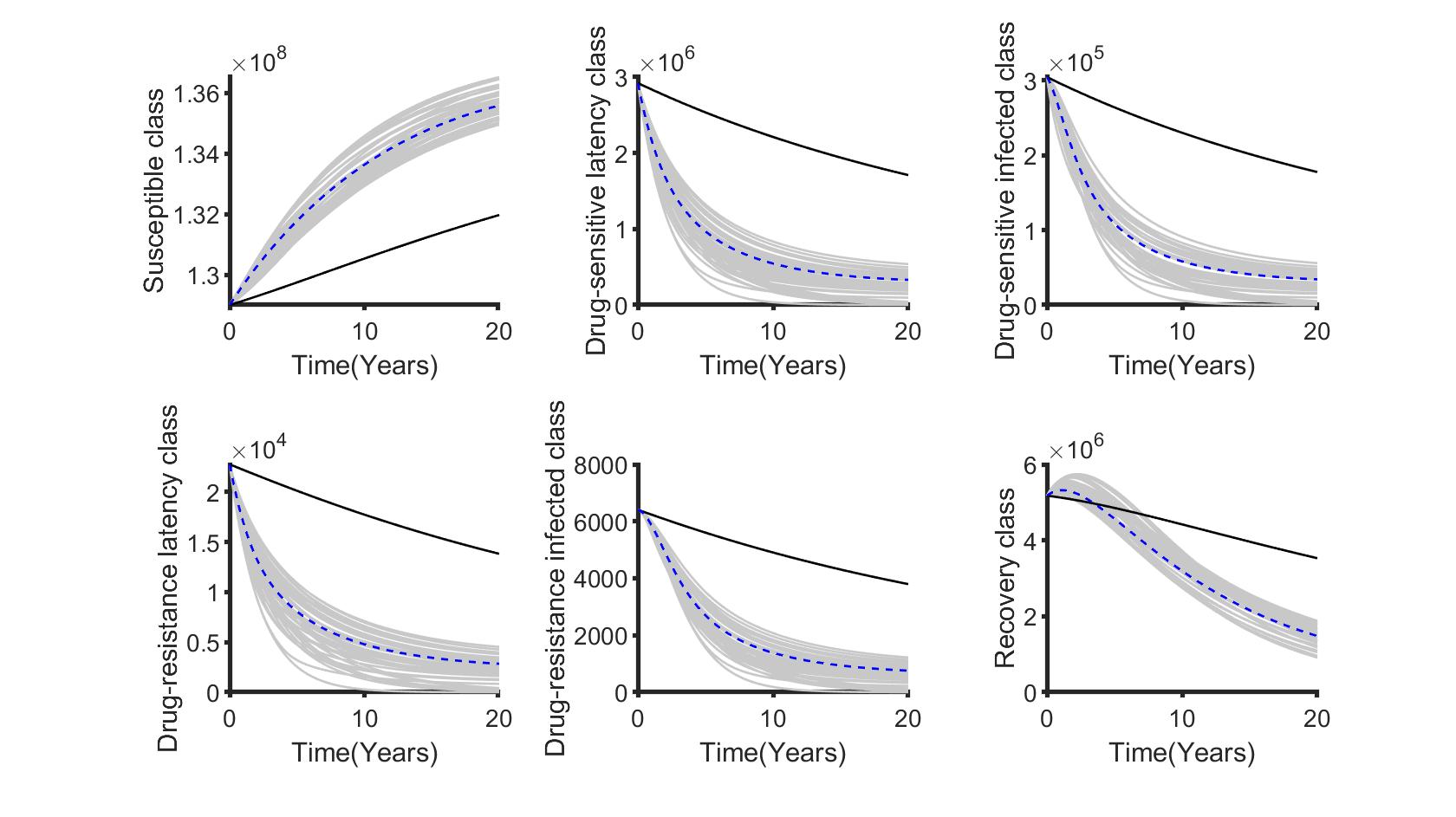
**

**S13 Fig (E).** The corresponding state variables of the combination of distancing control$(u_{1})$, latent case finding $(u_{2})$, case holding $(u_{3})$ and active case finding $(u_{4})$ control strategy when the weighted cost $B_{4}$ is varied and $B_{1}{=B}_{2}{=B}_{3}=10^{5}=10^{6}=10^{7}$. The state variables with and without controls are plotted by grays and black lines respectively.

**References**

1. Pontryagin LS. Mathematical theory of optimal processes: Routledge; 2018.
